# Unraveling the Potential of MXenes in Electrochemical Sensing: Mechanistic Insight, Design Principles, and Analytical Applications

**DOI:** 10.1002/smsc.202600005

**Published:** 2026-05-21

**Authors:** Hichem Moulahoum, Faezeh Ghorbanizamani, Tomáš Kovářík, Kalim Deshmukh

**Affiliations:** ^1^ Department of Biochemistry Faculty of Science Ege University Bornova Izmir Türkiye; ^2^ New Technologies Research Centre University of West Bohemia in Pilsen Pilsen Czech Republic; ^3^ Department of Material Science and Metallurgy Faculty of Mechanical Engineering University of West Bohemia in Pilsen Pilsen Czech Republic

**Keywords:** 2D nanomaterials, biosensing interfaces, electrochemical detection, flexible diagnostic devices, surface functionalization

## Abstract

MXenes, a rapidly expanding class of 2D transition metal carbides, nitrides, and carbonitrides, have emerged as promising materials for electrochemical sensing due to their metallic conductivity, hydrophilic surfaces, and tunable surface terminations. These properties enable efficient electron transfer, strong interfacial interactions, and versatile functionalization. However, despite extensive research, a clear mechanistic understanding of how MXene properties govern sensing performance remains limited. This review presents a mechanistic perspective on MXene‐based electrochemical sensors, emphasizing the relationships between composition, surface chemistry, structural organization, and analytical performance. Key material characteristics—including surface terminations, interlayer interactions, and synthesis strategies—are examined in terms of their roles in charge transfer, catalytic activity, and analyte adsorption. A unified framework is proposed in which MXenes function as conductive scaffolds, electrocatalytic interfaces, and adsorption‐driven platforms. Recent advances in major application areas, including small molecules, environmental pollutants, biomolecules, and pharmaceuticals are discussed, highlighting common design strategies and synergistic effects in hybrid systems. Finally, current challenges related to stability, reproducibility, and standardization are discussed. This perspective provides design guidelines for the development of next‐generation MXene‐based electrochemical sensors and supports their translation into practical applications.

## Graphical abstract

This review comprehensively examines recent progress in MXene‐based electrochemical sensors for the detection of glucose, hydrogen peroxide, heavy metals, pesticides, nitrites, phenolic pollutants, neurotransmitters, biomarkers, and pharmaceutical compounds. It offers a distinctive synthesis–property–application perspective by critically correlating preparation methods, surface functionalization strategies, and intrinsic material characteristics with sensing performance.

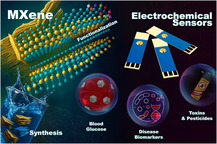

**Abbreviations**
2D: Two‐dimensional
3D: Three‐dimensional
AA: Ascorbic acid
AFP: Alpha‐fetoprotein
AI: Artificial intelligence
AMP: Amperometric
APS: Ammonium persulfate
AChE: Acetylcholinesterase
BPA: Bisphenol A
CEA: Carcinoembryonic antigen
CNTs: Carbon nanotubes
CV: Cyclic voltammetry
CVD: Chemical vapor deposition
DA: Dopamine
DFT: Density functional theory
DMSO: Dimethyl sulfoxide
DPV: Differential pulse voltammetry
EIS: Electrochemical impedance spectroscopy
ELISA: Enzyme‐linked immunosorbent assay
FET: Field‐effect transistor
GOx: Glucose oxidase
HF: Hydrofluoric acid
HCl: Hydrochloric acid
H_2_O_2_
: Hydrogen peroxide
LiF: Lithium fluoride
LOD: Limit of detection
LOQ: Limit of quantification
MAX: Layered carbides/nitrides
MOFs: Metal‐organic frameworks
MXene 2D transition metal xarbide, nitride: 2D transition metal xarbide, nitride, or carbonitride with surface terminations
NIR: Near‐infrared
NPs: Nanoparticles
PBS: Phosphate‐buffered saline
PCR: Polymerase chain reaction
PDDA: Poly(diallyldimethylammonium chloride)
POC: Point‐of‐care
SEM: Scanning electron microscopy
SPR: Surface plasmon resonance
TBA^+^
: Tetrabutylammonium ion
WHO: World Health Organization

## 1 Introduction

Electrochemical sensing has become an indispensable analytical strategy for the detection of chemical and biological species across diverse domains, including environmental monitoring, food safety, industrial process control, and clinical diagnostics, owing to its intrinsic sensitivity, rapid response, cost‐effectiveness, and compatibility with miniaturized and portable devices [1, 2, 3]. The growing demand for real‐time, on‐site, and point‐of‐care analysis has driven significant efforts toward the development of advanced electrode materials that enhance electron transfer kinetics, improve selectivity, and ensure reliable operation in complex matrices. In this context, nanostructured materials have played a transformative role by providing increased active surface area, tunable physicochemical properties, and enhanced interfacial interactions with target analytes [4]. Among these, 2D materials have attracted considerable attention due to their structural anisotropy, high surface‐to‐volume ratio, and exceptional electronic properties [5]. Graphene and its derivatives, along with transition metal dichalcogenides (TMDs), have been extensively explored for electrochemical sensing applications. However, despite their promising attributes, these materials exhibit inherent limitations that hinder broader applicability. Graphene‐based systems often suffer from restacking, limited surface functionalization, and hydrophobicity, which restrict analyte accessibility and dispersion in aqueous media [6]. Similarly, many TMDs exhibit semiconducting behavior with relatively low intrinsic conductivity, limiting charge transfer efficiency and sensor responsiveness unless further modified [6]. These limitations have prompted the exploration of alternative 2D material platforms capable of simultaneously delivering high electrical conductivity, chemical versatility, and strong interfacial affinity toward analytes.

**FIGURE 1:**
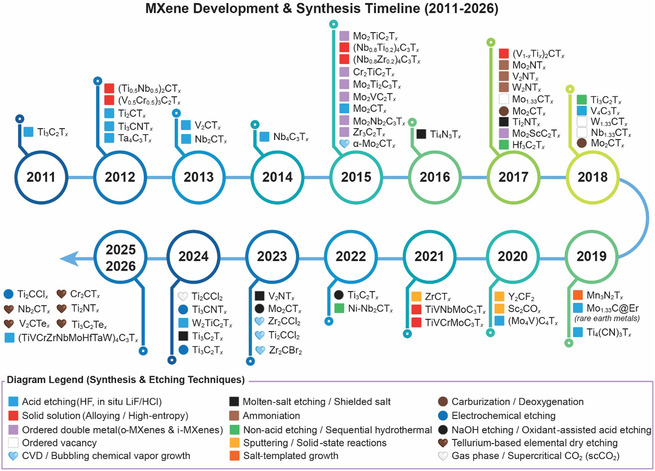
Timeline of major milestones in the development of MXene from 2011 to 2026. Figure designed based on the information collected from the literature [7, 10, 13, 14, 15, 16, 17, 18, 19, 20, 21, 22, 23, 24, 25, 26, 27, 28, 29].

**FIGURE 2:**
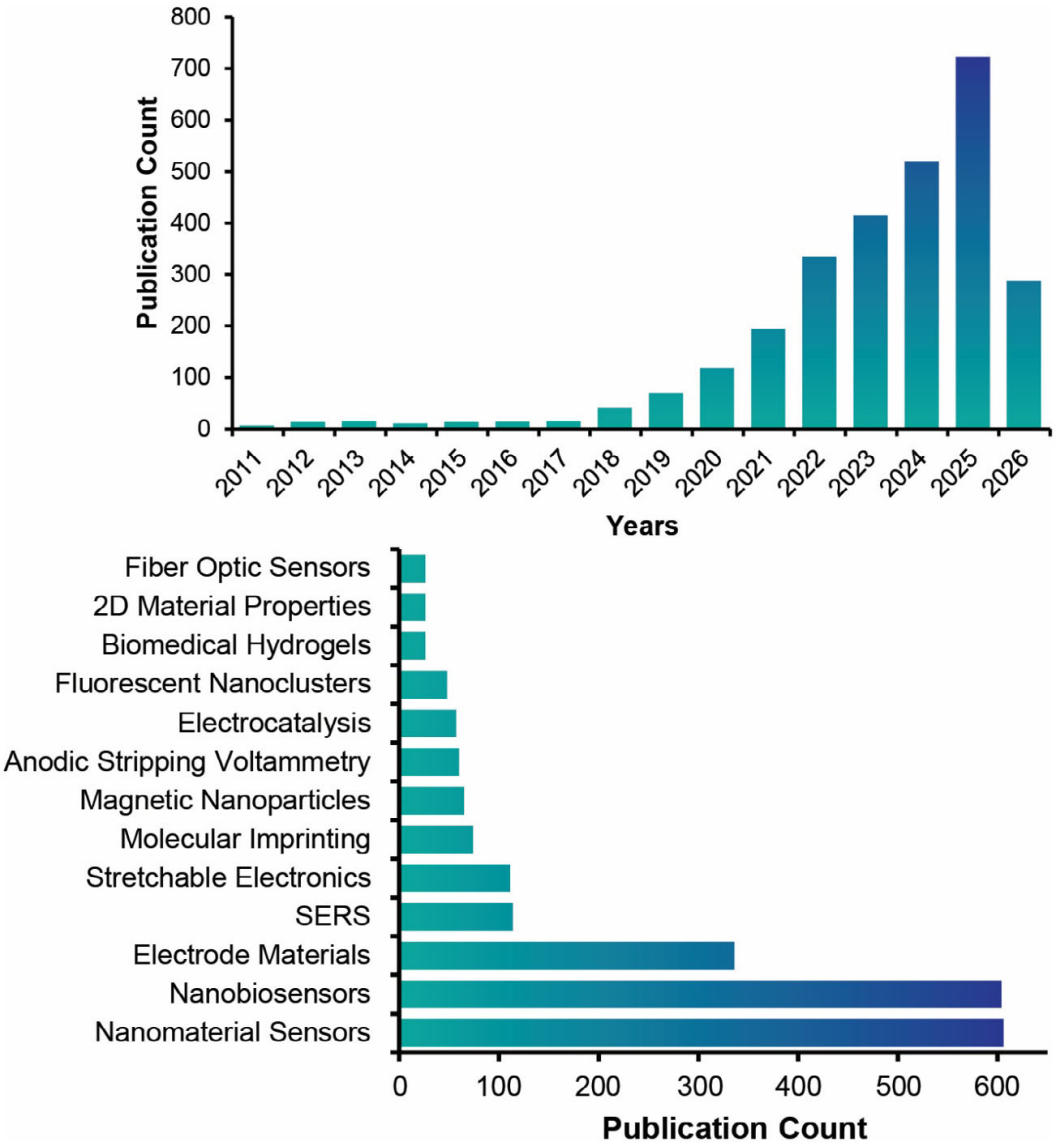
Web of Science query of publications about MXene‐based electrochemical sensors through the years (update as of March 30, 2026) showing both year‐based publication count (top) and topic distribution (bottom).

In this regard, MXenes—a rapidly expanding class of 2D transition metal carbides, nitrides, and carbonitrides—have emerged as highly promising candidates for electrochemical sensing applications since their first introduction in 2011 [7, 8]. MXenes are typically synthesized via selective etching of the A‐layer from MAX phases (M_*n* + 1_AX_*n*_, where M is an early transition metal, A is a group 13–16 element, and X is carbon and/or nitrogen), yielding layered structures with the general formula M_*n* + 1_X_*n*_T_*x*_, where T_*x*_ denotes surface terminations such as –O, –OH, and –F [7, 8, 9]. This unique structural motif endows MXenes with a rare combination of metallic conductivity, hydrophilicity, and chemically active surfaces, which is seldom observed simultaneously in other 2D materials [10, 11, 12]. Since their discovery, the number of experimentally realized MXene compositions has steadily expanded (Figure 1) [7, 10, 13, 14, 15, 16, 17, 18, 19, 20, 21, 22, 23, 24, 25, 26, 27, 28, 29], accompanied by rapid growth in publications related to electrochemical sensing applications (Figure 2). Early studies (2011–2014) focused primarily on synthesis and fundamental characterization, while the first sensing demonstrations emerged in 2015–2016 through protein‐ and enzyme‐modified Ti_3_C_2_T_*x*_ biosensors [11, 12]. Research between 2017 and 2018 expanded toward composite designs and a broader range of electroactive targets. From 2019 to 2021, applications advanced into environmental monitoring and biomedical diagnostics, with increasing emphasis on real‐sample analysis. Since 2022, the field has shifted toward device‐oriented formats—including flexible sensors, miniaturized systems, and point‐of‐care platforms—reflecting a transition from material development to application‐driven design. This evolution underscores the need for a structured and mechanistic understanding of MXene‐based sensing strategies and their associated challenges.

One of the defining features of MXenes is their high electrical conductivity, which can reach values on the order of 10^3^–10^4 ^S cm^−1^ [30] and, in some cases (e.g., Ti_3_C_2_T_*x*_), exceed 20,000 S cm^−1^ depending on composition and synthesis conditions [31, 32]. This metallic‐like behavior facilitates rapid electron transfer at the electrode interface, a critical requirement for high‐performance electrochemical sensing. In addition, surface terminations provide abundant active sites for adsorption, catalytic interactions, and immobilization of functional species, thereby enhancing sensitivity and selectivity [33, 34]. Unlike hydrophobic graphene, MXenes exhibit intrinsic hydrophilicity due to their surface functional groups, enabling stable dispersion in aqueous media and improved interaction with polar analytes and biomolecules [35, 36]. These combined properties make MXenes particularly well suited for applications involving biological fluids and environmental samples. Beyond their intrinsic characteristics, MXenes offer exceptional tunability through compositional engineering, surface chemistry modulation, interlayer spacing control, and hybridization with other functional materials [37, 38]. Variations in transition metal composition (e.g., Ti‐, Nb‐, V‐, and Mo‐based MXenes) [38, 39], incorporation of nitrogen (carbonitrides), and the emergence of multimetal and high‐entropy MXenes have significantly expanded the accessible property space [8, 40]. Concurrently, surface termination engineering and postsynthesis functionalization enable precise control over interfacial interactions, allowing MXenes to function as conductive scaffolds, electrocatalytic platforms, or immobilization matrices for enzymes, antibodies, aptamers, and molecularly imprinted polymers (MIPs) [41, 42]. This versatility enables the rational design of sensing interfaces tailored to specific detection mechanisms and analyte classes. The structural characteristics of MXenes—particularly their layered morphology and tunable chemistry—play a critical role in electrochemical sensing. This tunability operates across multiple levels: surface terminations (–O, –OH, –F, etc.) can be adjusted to regulate hydrophilicity, charge density, and catalytic activity; compositional flexibility in the choice of transition metals and X elements (carbon and/or nitrogen) allows fine control over electronic structure and intrinsic reactivity; and interlayer engineering, via delamination or ion intercalation, increases accessible surface area, optimizes spacing, and facilitates mass transport of analytes and electrolytes within the electrode architecture. Furthermore, MXenes readily form functional interfaces with polymers, biomolecules, and electrolytes, enabling tailored charge–transfer dynamics and enhanced selectivity. These structural and chemical degrees of freedom make MXenes uniquely versatile platforms for high‐performance electrochemical sensing [43, 44]. However, these advantages are accompanied by challenges such as restacking, oxidation, and variability in surface terminations, which can compromise reproducibility and long‐term stability [45, 46]. Addressing these limitations is essential for translating MXene‐based materials into reliable sensing platforms.

Despite the rapid expansion of MXene‐based electrochemical sensors, the field remains fragmented in terms of design strategies, mechanistic understanding, and performance evaluation. Existing reviews have largely focused on general material properties, synthesis methods, or specific application areas, often providing descriptive summaries without establishing clear correlations between material design, interfacial chemistry, and sensing performance [10, 47, 48, 49, 50]. Moreover, recent advances—including fluoride‐free synthesis routes, environmentally benign processing strategies, and increasingly complex hybrid architectures—have yet to be systematically integrated into a unified framework for rational sensor design [27, 28, 51, 52, 53, 54, 55]. In addition, inconsistencies in reporting key electrochemical performance metrics, such as limits of detection, sensitivity, and stability, hinder direct comparison across studies. Variations in experimental conditions, electrode fabrication protocols, and analyte matrices further exacerbate this lack of standardization, underscoring the need for a more critical and structured analysis of the field.

**FIGURE 3:**
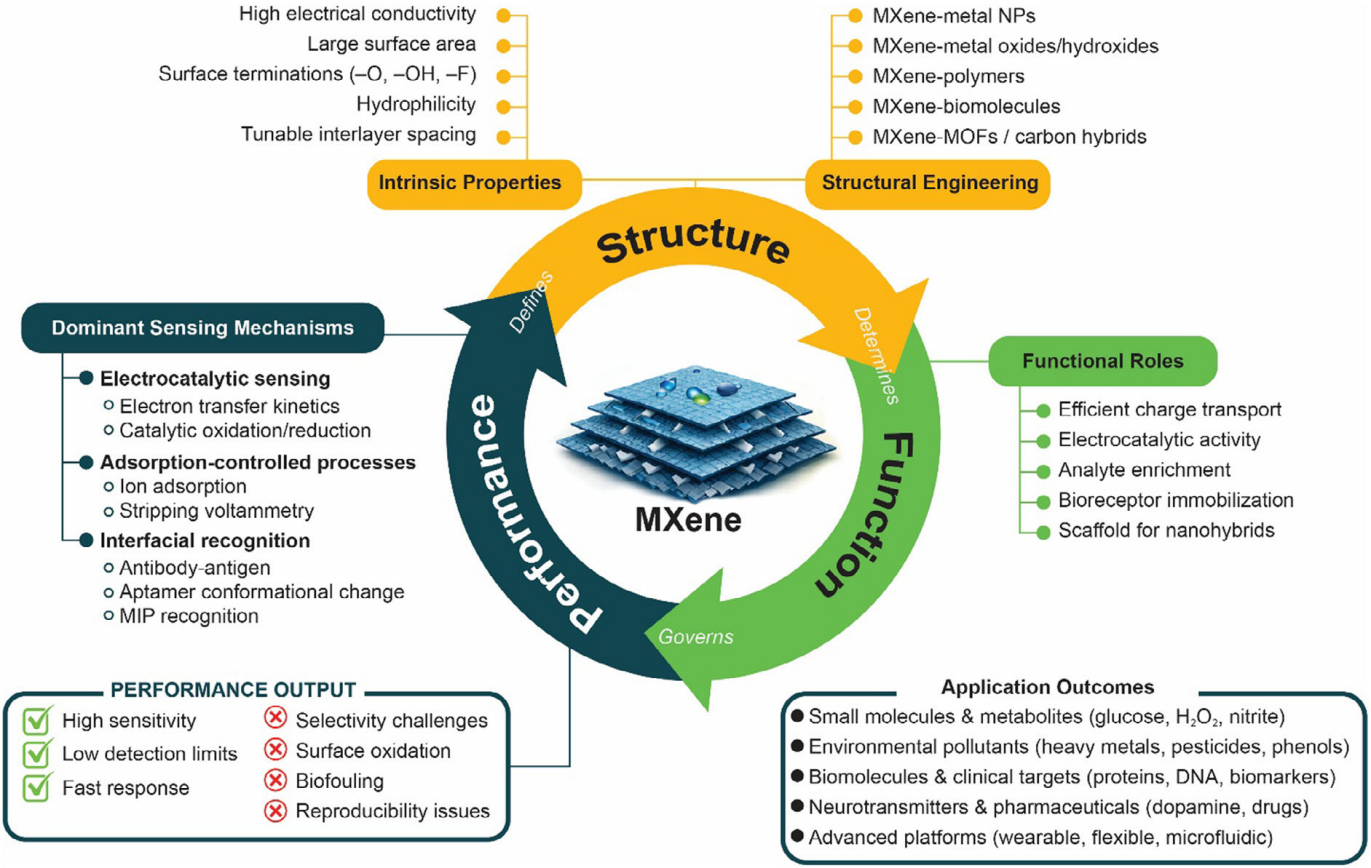
Structure–function–performance framework of MXene‐based electrochemical sensors showing how intrinsic properties and hybrid architectures define functional roles at the interface, including charge transport, electrocatalysis, analyte enrichment, and bioreceptor immobilization. These functions govern key sensing mechanisms and ultimately determine analytical performance such as sensitivity, detection limits, response time, and selectivity, across diverse application domains, while highlighting challenges including oxidation, biofouling, and reproducibility.

This review provides a comprehensive and critically structured examination of MXene‐based electrochemical sensors, with particular emphasis on elucidating the relationships between material composition, surface chemistry, structural engineering, and analytical performance (Figure 3). Key features relevant to sensing are first discussed in the context of synthesis and functionalization, establishing a performance‐oriented basis for understanding electrochemical transduction. Within this context, MXenes are shown to function as active components in signal generation and amplification, enabling diverse sensing interfaces across analyte classes ranging from small molecules and environmental contaminants to biomolecules and pharmaceuticals. A comparative analysis of recent advances identifies prevailing design strategies while highlighting persistent challenges related to stability, reproducibility, and scalability. Finally, emerging opportunities—including advanced MXene chemistries, sustainable synthesis approaches, and integration into flexible, wearable, and point‐of‐care devices—are outlined, positioning MXenes as a promising platform for next‐generation electrochemical sensing technologies.

## 2 MXene Features Relevant to Electrochemical Sensing

MXenes have attracted considerable attention in electrochemical sensing due to their unique combination of metallic conductivity, intrinsic hydrophilicity, tunable surface chemistry, and layered morphology. Although extensive research has focused on their synthesis and general physicochemical properties, only specific features directly govern their performance in electrochemical sensing platforms. In this section, key characteristics of MXenes are examined from a sensing‐oriented perspective, with emphasis on how composition, surface terminations, structural organization, and environmental stability collectively regulate interfacial electron‐transfer kinetics, analyte accessibility, and overall sensor performance.

### 2.1 Composition and Electronic Structure

The electronic properties of MXenes are primarily dictated by their transition metal composition and crystal structure, which together determine their conductivity and catalytic activity. Unlike many other 2D materials, several MXenes exhibit metallic or near‐metallic conductivity, with reported values reaching 20,000–24,000 S cm^−1^ for Ti_3_C_2_T_*x*_ films, depending on composition, synthesis conditions, and degree of delamination [31, 32]. This high electrical conductivity facilitates rapid charge transfer at the electrode–electrolyte interface, a key requirement for achieving high sensitivity and low detection limits in electrochemical sensing. The general formula M_*n* + 1_X_*n*_T_*x*_ enables significant compositional diversity, where M represents early transition metals such as Ti, Nb, V, or Mo and X corresponds to carbon and/or nitrogen [44]. Variations in the transition metal layers directly influence the density of states near the Fermi level, thereby modulating electron transport and catalytic behavior. For example, Ti_3_C_2_T_*x*_, the most extensively studied MXene, exhibits excellent conductivity and chemical stability, making it widely used in sensor fabrication [38]. In contrast, Nb‐ and Mo‐based MXenes (e.g., Nb_2_CT_*x*_, Nb_4_C_3_T_*x*_, Mo_2_CT_*x*_, Mo_2_TiC_2_F_*x*_) often display enhanced catalytic activity toward specific redox reactions, which is advantageous for detecting analytes such as hydrogen peroxide, nitrite, and biomolecules [56, 57, 58, 59]. Importantly, the metallic nature of MXenes distinguishes them from semiconducting 2D materials such as many transition metal dichalcogenides, which typically require additional modification to achieve sufficient conductivity for electrochemical applications [6]. Compared with graphene, MXenes offer not only comparable or higher conductivity in certain cases but also more accessible and chemically versatile surfaces, enabling stronger interactions with analytes and functional species [9, 37]. This combination of high conductivity and surface tunability underpins their effectiveness as electrochemical sensing platforms.

**FIGURE 4:**
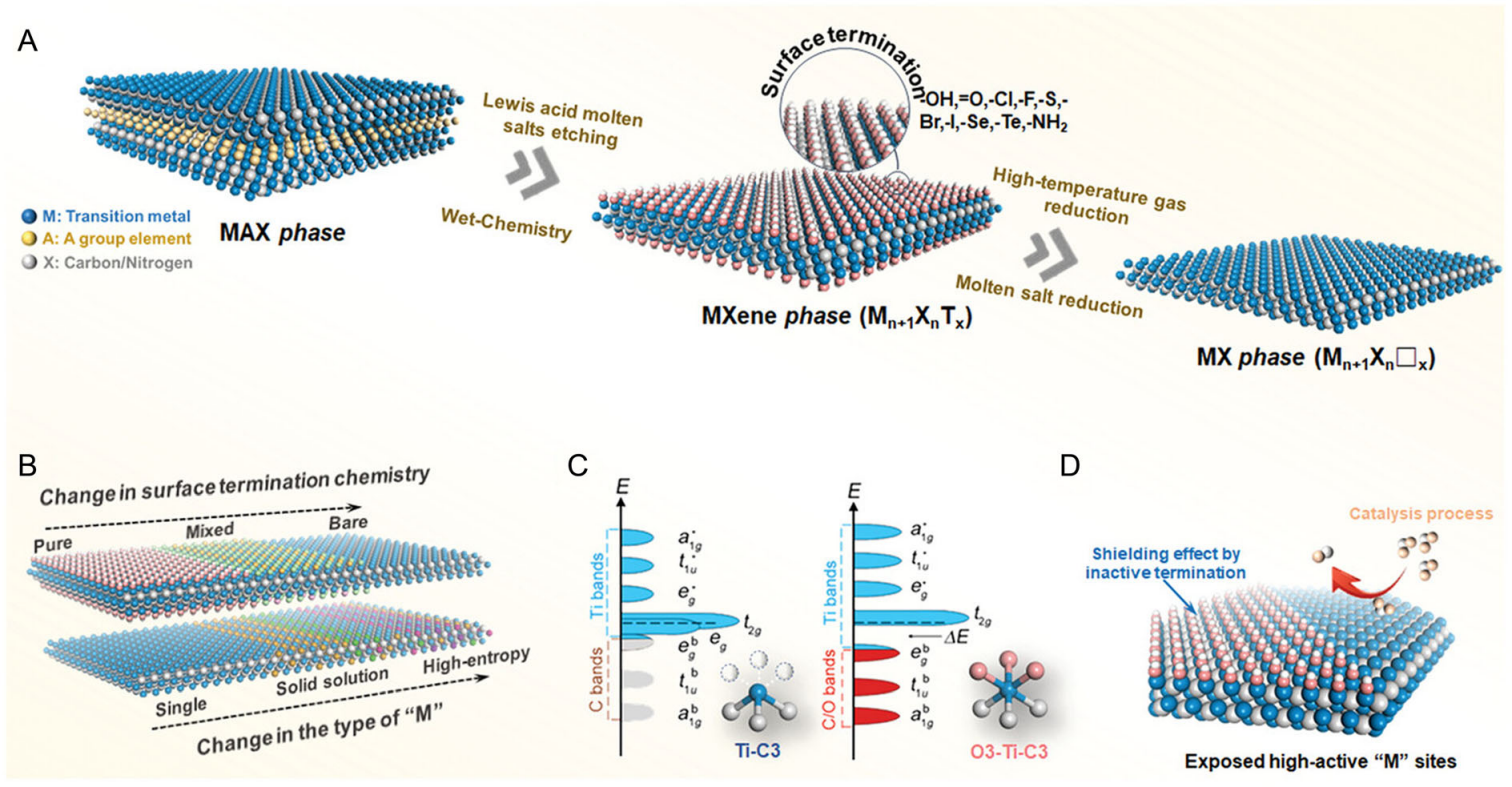
(A) Conceptual diagram illustrating the transformation from MAX phases to MXenes and subsequently to MX structures [63]. Copyright 2023. Reproduced with permission from John Wiley & Sons (B) Overview of emerging research directions in MXene science, emphasizing how compositional and structural variations influence their intrinsic properties [63]. Copyright 2023. Reproduced with permission from John Wiley & Sons. (C) Atomic‐scale depiction of surface titanium coordination and orbital configurations in pristine Ti_3_C_2_ and oxygen‐functionalized Ti_3_C_2_O_2_, with the Fermi level indicated by a dashed line at 0 eV [64]. Copyright 2017. Reproduced with permission from the American Chemical Society. (D) Comparative schematic of active surface sites in functionalized versus unmodified MXenes [63]. Copyright 2023. Reproduced with permission from John Wiley & Sons. These structural and termination‐dependent variations are directly relevant to electrochemical sensing, as they govern charge–transfer kinetics, adsorption of analytes, and exposure of catalytically active sites that ultimately determine sensitivity and selectivity.

**FIGURE 5:**
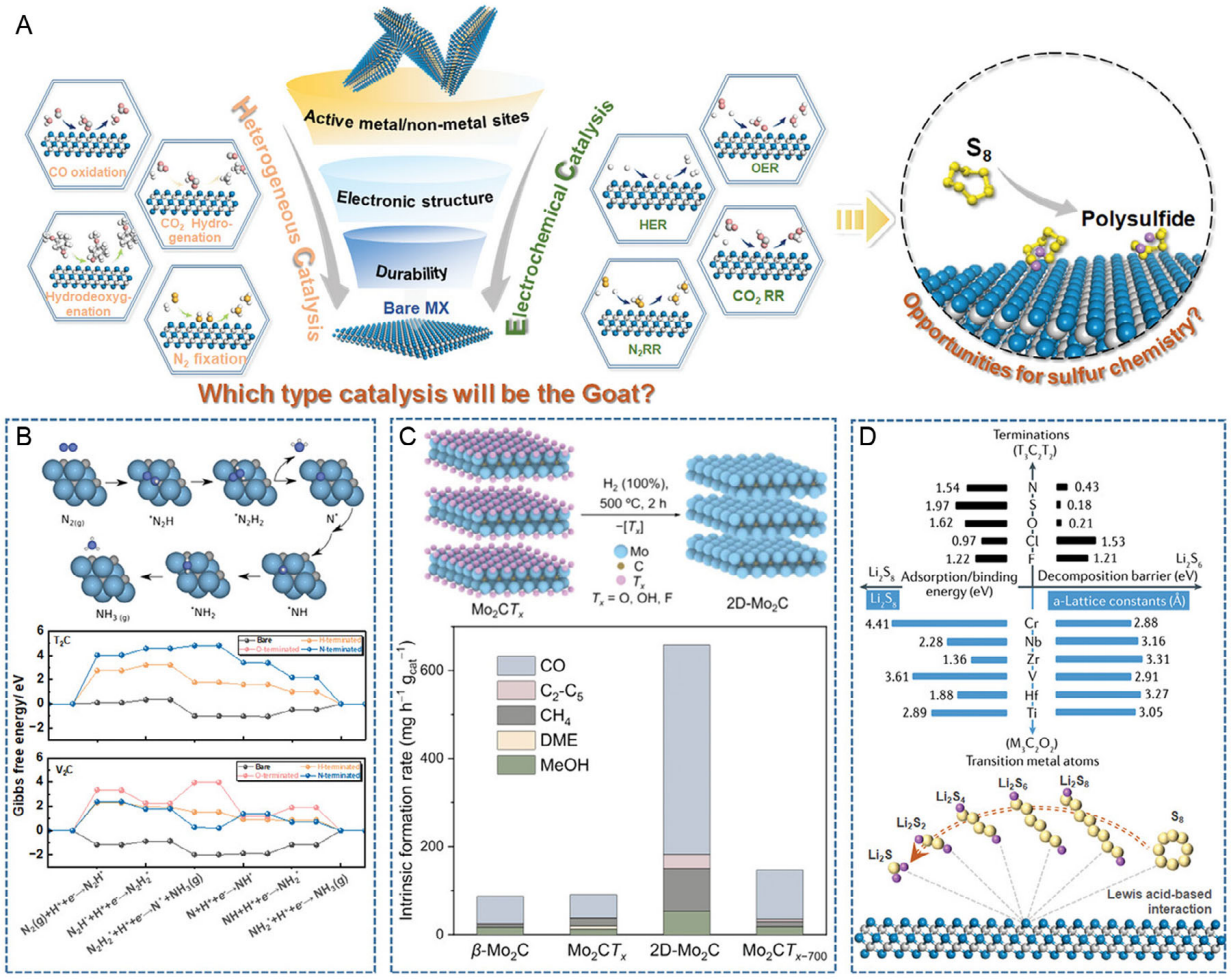
(A) Overview of core performance indicators of MX materials and their promising roles in heterogeneous and electrochemical catalysis, extending to redox processes [63]. Copyright 2023. Reproduced with permission from John Wiley & Sons. (B) Diagram of the reaction mechanism and computed free energy profiles for adsorbed species during electrochemical nitrogen reduction to ammonia, highlighting the influence of various surface terminations [66]. Copyright 2019. Reproduced with permission from the American Chemical Society. (C) Schematic representation of the conversion from Mo_2_CT_*x*_ to bare Mo_2_C, along with a comparative analysis of intermediate formation rates [65]. Copyright 2021. Adapted from Springer Nature. (D) Illustration of MXene‐based catalysts facilitating sulfur redox reactions by stabilizing polysulfides via Lewis acid–base interactions, with a comparative summary of how metal centers (M) and surface terminations (T_*x*_) affect polysulfide adsorption [67]. Copyright 2022. Reproduced with permission from Springer Nature.

Recently, termination‐free MX (hereafter MX) has emerged as a new subclass of 2D carbides obtained by removing surface groups from MXenes via high‐temperature treatment or chemical reduction [60, 61, 62]. Unlike conventional MXenes stabilized by surface terminations (T_*x*_), MX consists of pristine M_*n* + 1_X_*n*_ layers, exhibiting clean metallic surfaces and distinct physicochemical behavior (Figure 4) [63, 64]. This structural evolution from MAX → MXene → MX illustrates how compositional and surface engineering governs electronic and catalytic properties. In particular, the removal of terminations restores metallic character and exposes additional active sites, often leading to enhanced intrinsic activity compared with terminated MXenes [63, 64]. MX phases such as Ti_3_C_2_ and Mo_2_C can be obtained via hydrogen thermal treatment or chemical reduction routes, enabling partial or complete removal of surface terminations [63]. This modification significantly alters the electronic structure and can enhance catalytic reactivity, as supported by density functional theory (DFT) studies. For instance, O‐terminated Mo_2_C exhibits good hydrogen evolution reaction (HER) activity, whereas bare Mo_2_C shows superior CO_2_ hydrogenation performance due to the presence of unsaturated metallic sites [65]. The catalytic versatility of MX extends across heterogeneous and electrochemical reactions, including CO oxidation, hydrodeoxygenation, oxygen evolution reaction (OER), HER, and nitrogen reduction reaction (NRR), as well as sulfur‐related chemistry (Figure 5) [63, 65, 66, 67].

Notably, termination‐free MX materials are particularly advantageous for heterogeneous catalysis due to exposed metal active sites, favorable electronic structure, and high thermal stability, whereas surface‐terminated MXenes are generally more suitable for aqueous electrochemical systems. In sulfur‐based chemistry, strong interactions between MXene surfaces and sulfur species (e.g., polysulfides) can significantly influence reaction pathways; however, these systems are primarily relevant to battery applications and are beyond the scope of this review [68]. Despite these advances, bare MX phases suffer from poor stability in aqueous environments and challenges in achieving uniform termination removal, limiting their applicability mainly to non‐aqueous or high‐temperature conditions [63]. Nevertheless, the MAX → MXene → MX transformation highlights how surface engineering can tune the balance between stability, activity, and electronic structure. Future strategies such as post‐treatment modification, encapsulation, and hybrid material design may help overcome these limitations, enabling multifunctional MX‐based materials for catalysis, energy storage, and sensing applications.

### 2.2 Surface Terminations and Interfacial Chemistry

**FIGURE 6:**
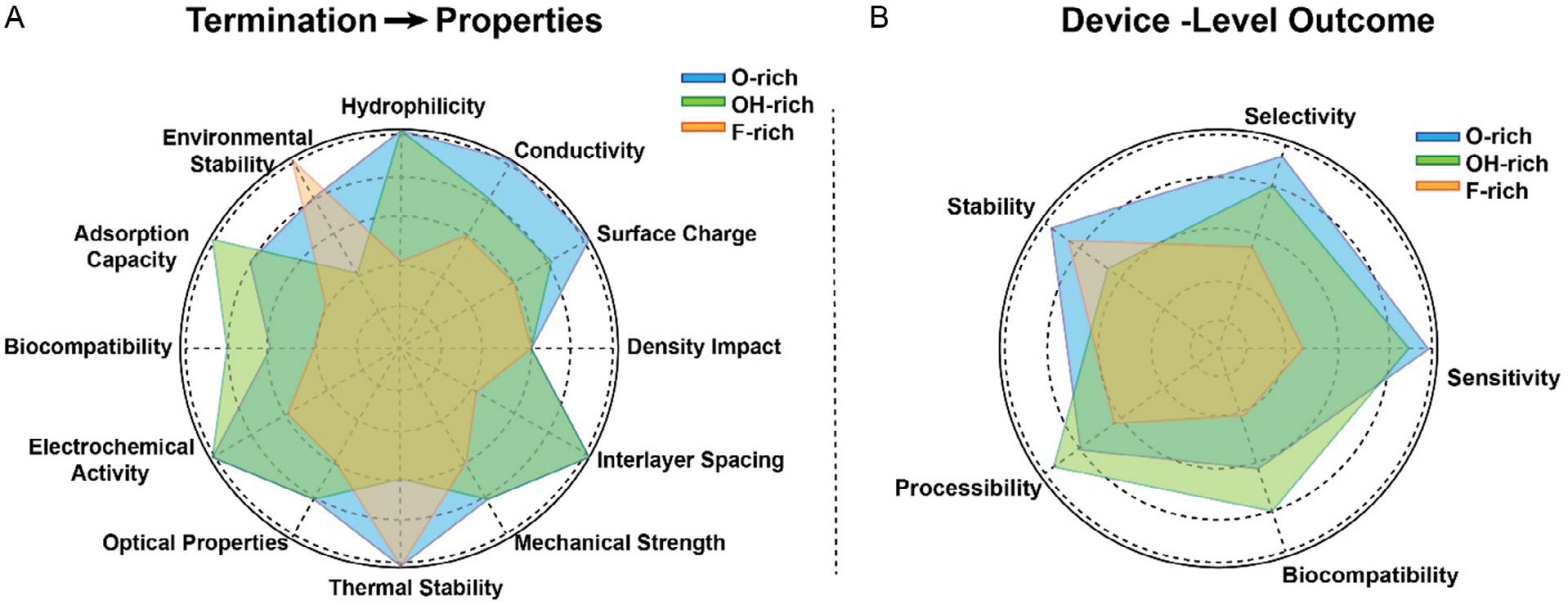
Comparative influence of MXene surface terminations on material and device properties. (A) Radar plot showing how O‐rich, OH‐rich, and F‐rich terminations affect intrinsic characteristics. (B) Device‐level outcomes highlighting differences in sensitivity, selectivity, stability, processability, and biocompatibility. Scores are not absolute but are relative performance trends across the literature.

One of the defining characteristics of MXenes is the presence of surface terminations (T_*x*_), typically consisting of –O, –OH, and –F groups introduced during the etching process. These functional groups play a crucial role in governing the interfacial properties of MXenes, influencing hydrophilicity, adsorption behavior, catalytic activity, and interactions with biomolecules [44, 69]. From a mechanistic perspective, the nature and distribution of surface terminations determine the types of intermolecular interactions that MXene nanosheets can establish with surrounding species, thereby directly influencing sensing performance (Figure 6). Hydroxyl (–OH) and oxygen (–O) terminations enable hydrogen bonding with polar molecules and biomacromolecules, which is particularly important for immobilizing enzymes, nucleic acids, and other recognition elements on electrode surfaces [70, 71]. In parallel, negatively charged surface groups facilitate electrostatic interactions with cationic species, promoting adsorption and preconcentration of analytes such as metal ions, neurotransmitters, and positively charged biomolecules [43, 72].

Surface terminations also strongly influence inter‐sheet interactions within MXene assemblies, thereby governing restacking behavior. The layered structure is stabilized through a combination of van der Waals interactions between basal planes, hydrogen bonding between surface groups, and, in some cases, cation‐mediated bridging via intercalated ions such as Li^+^, Na^+^, or multivalent species [9, 37, 43]. While hydrogen bonding between –OH groups can promote aggregation, van der Waals forces further drive restacking during drying or electrode fabrication. In addition, intercalated cations can reduce electrostatic repulsion and act as interlayer linkers, modulating nanosheet spacing. Although these interactions contribute to structural stability, excessive restacking significantly reduces accessible surface area, limits active site exposure, and impedes analyte diffusion, ultimately diminishing electrochemical performance [73, 74]. This limitation is particularly pronounced in drop‐cast or thick‐film electrodes, where solvent evaporation induces capillary‐driven compaction. Accordingly, various strategies have been developed to mitigate restacking, including intercalation of organic molecules, incorporation of spacer materials (e.g., nanoparticles (NPs), polymers, or carbon nanostructures), and controlled delamination processes, all aimed at preserving accessible electroactive surfaces [44, 75, 76, 77].

In addition to structural effects, surface terminations modulate the electronic structure and catalytic behavior of MXenes. Oxygen‐terminated surfaces are generally associated with enhanced electron‐transfer kinetics and improved catalytic activity toward redox‐active analytes, whereas high concentrations of fluorine terminations can introduce electronic localization and reduce conductivity [71, 78, 79]. Thus, the relative distribution of termination groups plays a decisive role in determining overall sensor performance. Importantly, the dynamic and often heterogeneous nature of surface terminations introduces sample‐to‐sample variability in interfacial behavior. Subtle changes in synthesis conditions can significantly alter termination composition, thereby affecting adsorption affinity, electron‐transfer kinetics, and long‐term stability [69, 80]. This sensitivity highlights the necessity of precise control and rigorous characterization of MXene surface chemistry to achieve reproducible and high‐performance electrochemical sensing platforms.

### 2.3 Interlayer Structure and Mass Transport

MXenes possess a layered structure analogous to other 2D materials, but with distinct interlayer chemistry that plays a critical role in electrochemical sensing and their interlayer spacing can be tuned through intercalation of ions, molecules, or organic species, as well as through delamination processes that yield few‐layer or single‐layer nanosheets [43, 44]. These structural modifications directly influence the accessibility of active sites and the diffusion of analytes and electrolytes within the electrode matrix. Delaminated MXene nanosheets provide high specific surface area and a large number of exposed active sites, which are beneficial for enhancing sensitivity and facilitating rapid electron transfer [81, 82]. In parallel, controlled interlayer expansion can generate nanochannels that promote efficient mass transport, leading to faster response times and improved detection performance. These features are particularly important for sensing large biomolecules or complex real‐world samples, where diffusion limitations can significantly affect sensor efficiency. However, MXenes are prone to restacking due to van der Waals interactions between adjacent layers, which reduces accessible surface area and impedes mass transport [73, 74]. This restacking not only decreases sensitivity but can also compromise reproducibility by altering the effective electroactive surface. To address this limitation, various strategies have been developed, including the incorporation of spacer materials such as NPs, polymers, or carbon‐based nanostructures, which help maintain interlayer separation and improve structural stability [73, 74, 75, 76, 77]. Overall, balancing high surface accessibility with structural integrity remains a key design consideration in MXene‐based electrochemical sensors.

### 2.4 Stability, Oxidation, and Reproducibility

Despite their promising properties, MXenes face significant challenges related to chemical stability, particularly under ambient and aqueous conditions. MXenes, especially Ti_3_C_2_T_*x*_, are susceptible to oxidation, leading to the formation of metal oxides such as TiO_2_, which can alter electrical conductivity and surface chemistry [83]. This degradation process results in diminished sensor performance over time, affecting both sensitivity and reproducibility.

Oxidation is strongly influenced by factors such as temperature, pH, dissolved oxygen, and storage conditions, with aqueous dispersions being particularly prone to degradation [46, 84]. While controlled oxidation can, in some cases, introduce additional catalytic sites, uncontrolled processes generally lead to performance deterioration and increased variability in electrochemical response.

Reproducibility is further compromised by variations in synthesis methods, surface terminations, and electrode fabrication protocols. Differences in etching conditions, delamination procedures, and post‐synthetic functionalization can result in significant batch‐to‐batch variability, limiting direct comparison across studies [69, 85]. This lack of standardization remains a major barrier to the widespread adoption of MXene‐based sensors and highlights the need for reproducible, scalable, and well‐controlled fabrication strategies.

### 2.5 Mechanical Properties and Device Integration

In addition to their electrical and chemical properties, MXenes exhibit excellent mechanical flexibility and processability, which are highly advantageous for the development of next‐generation electrochemical sensors. MXene films can be fabricated using solution‐based techniques such as drop‐casting, spin‐coating, and inkjet printing, enabling their integration into flexible, wearable, and microfluidic devices [35, 36]. The combination of high conductivity and mechanical robustness allows MXenes to maintain stable electrochemical performance under deformation, making them suitable for wearable sensing and real‐time monitoring applications [86, 87, 88]. Furthermore, their compatibility with polymer matrices and composite systems facilitates the design of hybrid architectures with enhanced mechanical stability and multifunctional performance [89]. Collectively, these attributes position MXenes as strong candidates for portable and user‐friendly sensing platforms, particularly in continuous monitoring and on‐site analytical applications. To contextualize MXenes within the broader family of 2D materials, Table 1 compares their physicochemical and sensing‐relevant properties with those of transition metal dichalcogenides, graphene derivatives, and conventional carbon‐based electrodes [31, 32, 90, 91, 92, 93, 94, 95, 96, 97, 98, 99, 100, 101, 102, 103, 104, 105, 106, 107, 108, 109, 110, 111, 112, 113, 114, 115, 116, 117, 118, 119, 120, 121, 122, 123]. MXenes uniquely combine high electrical conductivity, tunable surface chemistry, and stable aqueous dispersibility, which together underpin their strong electrochemical performance. In contrast, graphene offers excellent conductivity but limited intrinsic surface functionality for biomolecular modification, while transition metal dichalcogenides provide tunable semiconducting properties at the expense of charge transport efficiency. Conventional carbon materials, although chemically stable and widely used, generally exhibit lower electrochemical activity. This comparison highlights the distinctive position of MXenes as materials that integrate conductivity, chemical versatility, and interfacial tunability, explaining their superior performance in electrochemical sensing platforms [124, 125, 126].

**TABLE 1:** Comparison of key properties of MXenes and widely used 2D materials relevant to electrochemical sensing.

Property	**MXenes (Ti**_**3**_ **C**_**2**_ **T**_***x***_ **)**	**TMDs (e.g., MoS**_**2**_ **)**	Graphene	Activated carbon
Electrical conductivity, S/cm	9,880 (Ti_3_C_2_T_*x*_) [90]; 1,000–10,000 [91, 92]; 20,000 [32]; 21,000 [97], 24,000 [31]	5.0 (MoS_2_) [93]	10^6^ (pristine graphene) [94]; 1,000–10,000 [95]	<100 [96]
Specific capacitance, F/g	400–700 [91, 92]	—	200–350 [95]	80–150 [96]
Bandgap, eV	Metallic; tunable by terminations/intercalation [98, 99, 100]	1.8 (monolayer MoS_2_); 1.45 (WS_2_) [101]	0 (bilayer graphene) [101]	—
Surface chemistry/functionalization	Abundant ‐O, ‐OH, ‐F; easy biofunctionalization [102, 103]	Lacking dangling bonds; limited covalent functionalization [104, 105]	Lacking surface terminations for biofunctionalization [106, 107]	Limited surface chemistry
Dispersity in water	Stable aqueous dispersions due to negatively charged surface terminations (‐O^−^/‐OH^−^), yielding high negative zeta potential (typically −30 to −50 mV) and electrostatic stabilization [108]	Tendency to agglomerate [105]	Strong aggregation in water [109]	Poor intrinsic dispersibility
Stability (ambient/aqueous)	Vulnerable in humid/oxidative conditions [110]	Degrades under oxygen/moisture [111]	Relatively stable in ambient conditions [112, 113]	Excellent chemical stability
Biosafety	Good biocompatibility, negligible cytotoxicity [114, 115]	Few cytotoxic responses reported [116, 117]	Low cytotoxicity, good biocompatibility [99, 118]	Generally biocompatible
Key advantages in sensing	High conductivity, tunable chemistry, strong adsorption and chelation with biomolecules [119, 120]	Au‐S chemistry enables integration with noble metals [121, 122]	Fast charge transfer, good mechanical properties [123]	Low cost, stability
Main limitations	Oxidation sensitivity, synthesis variability [91, 92]	Low conductivity, environmental instability	Limited functionalization, aggregation	Poor conductivity, low electroactivity

## 3 Synthesis and Functionalization of MXenes: Implications for Electrochemical Sensing

The synthesis and post‐synthesis modification of MXenes play a decisive role in determining their physicochemical properties and, consequently, their electrochemical sensing performance. While numerous studies have reported various preparation routes, the relevance of these methods to sensing applications lies primarily in their influence on surface terminations, defect density, interlayer structure, and dispersion stability. Therefore, rather than providing an exhaustive overview of synthetic procedures, this section focuses on key synthesis and functionalization strategies that directly govern electrochemical behavior.

### 3.1 Etching Strategies and Their Consequences

MXenes are primarily synthesized through selective removal of the A‐layer from MAX phases (M_*n* + 1_AX_*n*_), most commonly using fluoride‐containing etchants such as hydrofluoric acid (HF) or in situ generated HF from mixtures of fluoride salts (e.g., LiF) and strong acids [8, 9, 53]. These approaches remain the most widely used for producing MXenes in electrochemical sensing, as they provide relatively high yields and well‐defined layered structures with surface terminations including –F, –O, and –OH [127, 128]. The choice of etching route has a direct and critical influence on surface chemistry and defect formation. HF‐based etching typically yields MXenes with a higher proportion of fluorine terminations, which can enhance structural stability but may reduce electrical conductivity and block active sites when present in excess [129, 130]. In contrast, milder in situ HF methods often produce MXenes enriched in oxygen‐ and hydroxyl‐terminated surfaces, which are more favorable for electrochemical sensing due to improved hydrophilicity, enhanced electron‐transfer kinetics, and stronger analyte interactions [85, 130].

**FIGURE 7:**
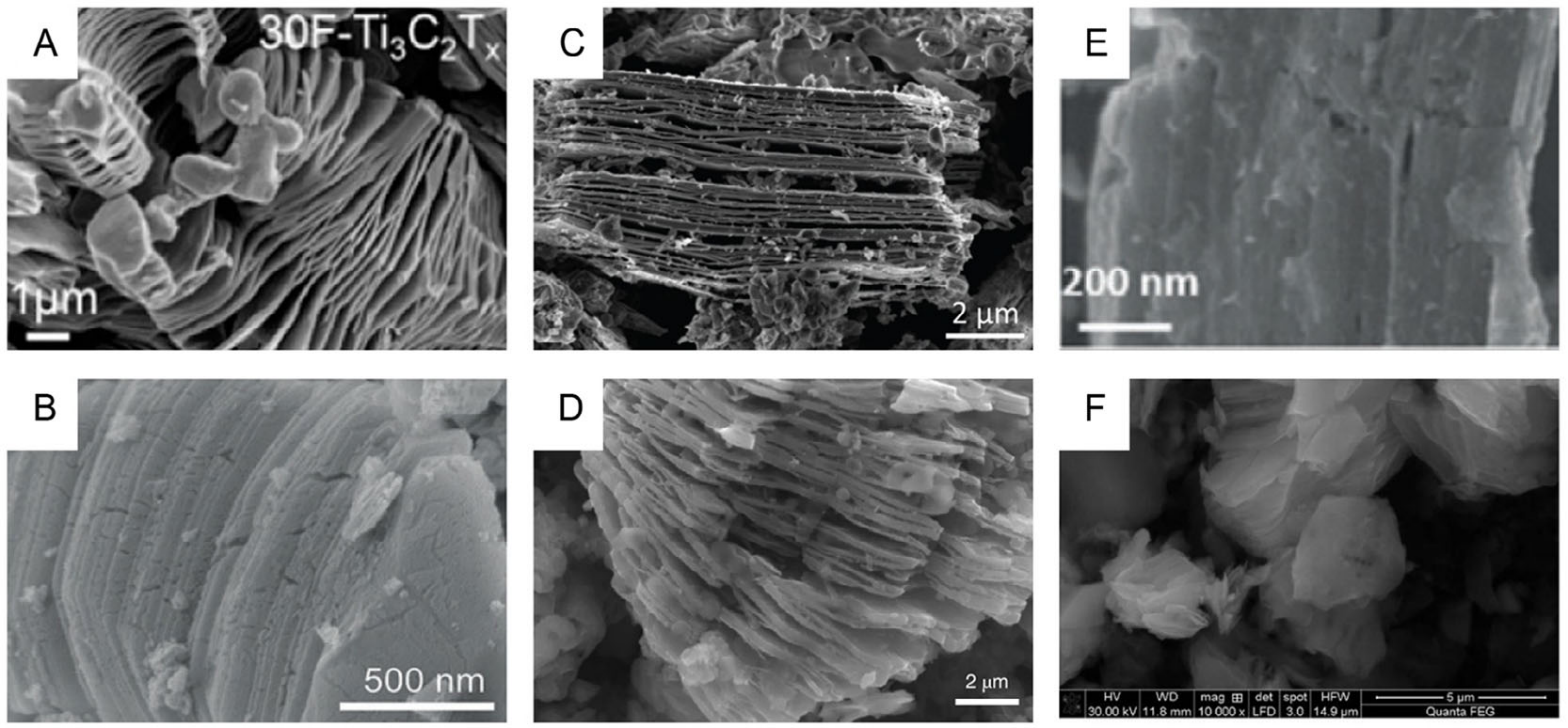
SEM images of MXene prepared using different etching approaches. (A) HF etching [51]. Copyright 2017. Reproduced with permission from the American Chemical Society. (B) Alkali Etching [27]. Copyright 2016. Reproduced with permission from the Royal Society of Chemistry. (C) Molten Salt Etching [53]. Copyright 2018. Reproduced with permission from John Wiley & Sons. (D) Lewis Molten Salt Ion Exchange Etching [28]. Copyright 2018. Reproduced with permission from John Wiley & Sons. (E) Electrochemical Etching [54]. Copyright 2020. Reproduced with permission from The Author(s) with exclusive license to Springer Nature. (F) CVD method [52]. Copyright 2021. Reproduced with permission from Elsevier.

Beyond conventional approaches, several alternative synthesis strategies have been explored, including alkali‐assisted etching, electrochemical etching, molten salt (Lewis acid) etching, and gas‐phase treatments, all of which yield variations in structure and morphology, as observed by scanning electron microscopy (SEM) (Figure 7) [27, 28, 51, 52, 53, 54]. For example, Lewis acid etching using molten salts (e.g., ZnCl_2_) enables MXenes with reduced fluorine content and modified surface terminations, while electrochemical etching provides improved control over reaction conditions and more uniform structures [69, 131, 132, 133]. Gas‐phase and chemical vapor‐based methods have also been investigated for synthesizing MXene‐like structures or modifying existing MXenes [29, 134, 135, 136]. Post‐synthesis ultrasonication is frequently used to promote delamination; however, excessive treatment can introduce structural defects and reduce flake size, thereby affecting electron transport pathways and electrochemical performance [137, 138]. Despite these advances, alternative synthesis routes remain less widely applied in electrochemical sensing compared to HF‐based and in situ HF methods, primarily due to challenges in scalability, reproducibility, and precise control over surface terminations—factors that are critical for sensor performance. Besides, etching conditions also govern defect density and flake morphology. Aggressive reaction conditions or prolonged etching can generate vacancies and structural distortions, which may enhance catalytic activity by creating additional active sites but can also degrade conductivity and structural stability if not properly controlled [139]. Therefore, careful optimization of synthesis parameters is essential for tailoring MXene properties for electrochemical sensing applications.

### 3.2 Delamination and Intercalation

Following etching, multilayer MXenes are often subjected to delamination processes to obtain few‐layer or single‐layer nanosheets with higher accessible surface area. This is typically achieved through intercalation of organic molecules or ions (e.g., dimethyl sulfoxide (DMSO), urea, or alkali metal ions), which expand the interlayer spacing and weaken inter‐sheet interactions [44, 75, 76, 77]. Delamination significantly enhances electrochemical performance by exposing a larger number of active sites and facilitating faster electron transfer. The resulting few‐layer MXenes also exhibit improved dispersion stability in aqueous media due to electrostatic repulsion arising from negatively charged surface terminations [81, 82], which is particularly beneficial for forming uniform electrode coatings and reproducible sensing interfaces. However, despite improved exfoliation, interlayer interactions such as van der Waals forces and hydrogen bonding can still induce restacking during solvent evaporation or electrode fabrication [9, 37]. In addition, intercalated cations may act as bridging species between adjacent layers, partially screening electrostatic repulsion and promoting aggregation [43]. As a result, the effectiveness of delamination must be considered alongside strategies aimed at preserving interlayer separation in practical device architectures. Importantly, controlled intercalation can also be exploited to tune interlayer spacing and engineer nanochannels that facilitate analyte diffusion and ion transport. This is particularly advantageous for sensing large biomolecules or in systems where mass transport limitations govern the overall response. Overall, the interplay between delamination, intercalation, and restacking represents a key design parameter in MXene‐based electrochemical sensors.

### 3.3 Functionalization Strategies and Hybrid Architectures

Beyond intrinsic MXene properties, functionalization and hybridization strategies are widely employed to enhance electrochemical sensing performance. These approaches address key limitations such as restacking, limited selectivity, and insufficient catalytic activity, while introducing functionalities tailored to specific analytes [71, 140, 141, 142, 143, 144].

#### 3.3.1 Nanoparticle Decoration

The incorporation of metal or metal oxide NPs (e.g., Au, Pt, Pd, TiO_2_) onto MXene surfaces is a widely used strategy to enhance catalytic activity and sensitivity [145, 146, 147]. In these hybrid systems, MXenes act as highly conductive scaffolds that facilitate rapid electron transfer, while NPs provide abundant active sites for redox reactions. Strong interactions between MXene surface terminations and NPs enable stable anchoring and uniform dispersion, which are essential for reproducible sensor performance. Such composites have been extensively applied in the detection of hydrogen peroxide, glucose, and other small molecules, where electrocatalytic processes dominate signal generation.

#### 3.3.2 Polymer and Molecularly Imprinted Systems

The integration of MXenes with conductive polymers or MIPs enables the development of highly selective sensing platforms [89, 148, 149, 150, 151, 152]. In these systems, MXenes act as conductive backbones that enhance signal transduction, while the polymer matrix provides molecular recognition sites specific to the target analyte. For example, electropolymerization of monomers such as o‐phenylenediamine or aniline in the presence of MXenes facilitates the formation of imprinted cavities with high affinity toward target molecules [148, 149, 150]. The synergy between MXene conductivity and polymer‐based molecular recognition leads to improved sensitivity, selectivity, and operational stability compared with conventional sensing platforms.

#### 3.3.3 Biomolecule Immobilization

MXenes have also been widely used as platforms for immobilizing biomolecules such as enzymes, antibodies, and aptamers, owing to their hydrophilicity and surface functional groups [153, 154, 155]. The presence of –OH and –O terminations facilitates both covalent and noncovalent attachment of biomolecules, preserving their biological activity while ensuring efficient electron transfer [153, 156, 157]. This capability has been widely exploited in biosensor development for glucose, neurotransmitters, and various disease‐related biomarkers, where the combination of biological recognition and MXene‐mediated signal amplification enables high‐performance detection. Representative examples of these applications are discussed in detail in Section 5.

#### 3.3.4 Carbon‐Based and Composite Structures

To mitigate restacking and enhance structural stability, MXenes are often combined with carbon‐based materials such as graphene, carbon nanotubes, or porous carbon frameworks [73, 74]. These materials act as spacers that prevent layer aggregation, preserve accessible surface area, and improve mechanical robustness. Such composite structures also enhance electron transport pathways and introduce hierarchical porosity, thereby facilitating mass transport and improving sensor response times. The synergistic interaction between MXenes and carbon materials is widely exploited in electrochemical sensing applications [73, 74, 158].

### 3.4 Synthesis–Structure–Performance Relationships

A key aspect of MXene‐based sensor design lies in understanding the relationship between synthesis conditions, resulting material properties, and electrochemical performance. Etching methods determine surface terminations and defect density; delamination and intercalation govern surface accessibility and mass transport; and functionalization strategies define selectivity and catalytic activity [44, 69, 75, 76, 77, 140, 141, 142, 143, 144]. These interdependent factors collectively control the behavior of MXene‐based electrodes and must be carefully optimized to achieve high‐performance sensing platforms. Importantly, the lack of standardized synthesis protocols and reporting practices often complicates direct comparison across studies, highlighting the need for more systematic investigation and rigorous characterization [46, 69].

## 4 Conceptual Framework for MXene‐Based Electrochemical Sensing

The analytical performance of electrochemical sensors is governed by the interplay between interfacial electron‐transfer kinetics, analyte transport, and surface interactions at the electrode–electrolyte interface. In MXene‐based systems, these processes are strongly influenced by the material's metallic conductivity, surface terminations, defect structure, and layered morphology [9, 33, 34, 37, 39, 159, 160]. Establishing a mechanistic framework that links these intrinsic properties to signal generation is therefore essential for understanding sensor behavior and enabling the rational design of high‐performance sensing platforms.

### 4.1 Electrochemical Transduction and Interfacial Charge Transfer

Electrochemical sensing relies on the conversion of chemical interactions into measurable electrical signals through faradaic and non‐faradaic processes [161, 162, 163]. In faradaic systems, the analytical signal arises from oxidation or reduction reactions at the electrode surface, where the measured current is governed by heterogeneous electron‐transfer kinetics and analyte mass transport to the interface. MXenes contribute to this process primarily through their high electrical conductivity, which originates from the high density of electronic states near the Fermi level [39]. This property facilitates rapid charge transfer and reduces charge–transfer resistance (*R*_ct_), thereby increasing the heterogeneous electron‐transfer rate constant (*k*_0_) [164]. As a result, MXene‐modified electrodes typically exhibit enhanced peak currents, lower overpotentials, and improved signal resolution. The layered structure of MXenes further provides interconnected conductive pathways that support electron percolation across nanosheets. However, this behavior strongly depends on interlayer organization. Delaminated or well‐dispersed MXene sheets enable efficient charge transport, whereas restacked structures introduce resistive barriers that hinder electron flow [73, 74]. Consequently, the electrochemical response is determined not only by intrinsic conductivity but also by the structural architecture of the material within the electrode. In non‐faradaic sensing approaches, such as electrochemical impedance spectroscopy, signal generation arises from changes in interfacial properties rather than redox reactions. In this case, MXenes modulate the electrical double layer and interfacial capacitance through their high surface area and chemically active interfaces [165, 166]. Adsorption of analytes or binding of recognition elements alters surface charge distribution and dielectric properties, producing measurable impedance changes. The sensitivity of such systems is therefore strongly dependent on the accessibility and chemical nature of surface terminations.

### 4.2 Mechanistic Roles of MXenes in Signal Generation

Beyond facilitating charge transfer, MXenes actively participate in electrochemical sensing through multiple mechanistic pathways arising from their surface chemistry and structural characteristics. The presence of transition metal atoms and functional surface terminations creates a heterogeneous interface capable of interacting with analytes via adsorption and catalytic processes. Oxygen‐ and hydroxyl‐terminated surfaces, in particular, promote adsorption through hydrogen bonding and electrostatic interactions, while also serving as catalytic sites that lower activation energy barriers for redox reactions [71, 78, 79, 167, 168]. Adsorption‐driven preconcentration represents a key signal amplification mechanism in MXene‐based sensors. Negatively charged surface terminations attract cationic species, increasing their local concentration at the electrode interface relative to the bulk solution [169, 170]. This enrichment effect is especially critical for trace detection, where analyte concentrations are inherently low. In addition to electrostatic interactions, coordination between analytes and exposed metal sites, along with hydrogen bonding with surface functional groups, further strengthens interfacial binding and enhances signal response.

MXenes also play an important role in establishing efficient electron‐transfer pathways within composite systems. When integrated with polymers, biomolecules, or other nanomaterials, they form conductive networks that enable charge transport across otherwise insulating or weakly conductive layers [89, 140, 141, 142, 143, 144]. This percolation effect is particularly important in sensing platforms employing molecular recognition elements, where direct electron transfer is often limited. In such architectures, MXenes function as electron mediators, bridging the recognition interface and the underlying electrode. In addition, MXenes frequently exhibit synergistic effects when combined with catalytic NPs. In these hybrid systems, MXenes facilitate rapid electron transport, while NPs provide highly active catalytic sites, resulting in enhanced overall electrochemical performance [145, 146, 147]. This synergy typically leads to improved sensitivity and lower detection limits compared with individual components, underscoring the importance of interfacial engineering in sensor design.

### 4.3 Mass Transport and Structural Effects

The layered architecture of MXenes introduces unique mass‐transport characteristics that significantly influence sensor performance. In delaminated or intercalated systems, expanded interlayer spacing creates accessible pathways that facilitate diffusion of analytes and electrolytes toward active sites [43, 44, 75, 76, 77]. This enhances response kinetics by promoting efficient interaction between analytes and the electrode surface, thereby improving sensitivity. However, the intrinsic tendency of MXenes to restack due to van der Waals interactions and hydrogen bonding between surface terminations can limit this advantage [9, 37, 73, 74]. Restacking reduces accessible surface area and introduces diffusion barriers, which hinder analyte transport and attenuate the electrochemical response. In addition, intercalated cations can modulate this behavior by promoting interlayer bridging and reducing electrostatic repulsion between nanosheets [43]. Consequently, the structural organization of MXenes within the electrode critically determines whether the sensing response is governed by electron‐transfer kinetics or mass‐transport limitations. The balance between these regimes depends on the degree of delamination, interlayer spacing, and overall electrode architecture. Careful control of this balance is therefore essential for achieving high‐performance MXene‐based electrochemical sensors.

### 4.4 Interpretation of Performance Metrics

Performance metrics such as sensitivity, limit of detection (LOD), linear dynamic range, and stability are commonly used to evaluate electrochemical sensors [171]; however, their interpretation in MXene‐based systems requires careful consideration of underlying mechanisms. Enhanced sensitivity may arise from a combination of increased active surface area, improved electron‐transfer kinetics, catalytic activity, and analyte preconcentration, making it difficult to attribute performance gains to a single dominant factor. Similarly, reported LOD values are strongly affected by experimental conditions, including baseline noise, electrode preparation, and analyte matrix effects, which complicates direct comparison across studies [171]. Stability is further governed by MXene oxidation, structural degradation, and the robustness of surface functionalization strategies [46, 83]. In the absence of standardized reporting practices, these metrics may therefore provide an incomplete or potentially misleading representation of sensor performance.

### 4.5 Challenges in Mechanistic Interpretation and Standardization

A major challenge in the field of MXene‐based electrochemical sensing is the lack of standardized methodologies for material preparation, characterization, and performance evaluation. Variations in synthesis conditions lead to differences in surface terminations, flake size, degree of delamination, and oxidation state, all of which can significantly influence electrochemical behavior [46, 69]. These inconsistencies hinder the establishment of clear structure–property–performance relationships. Furthermore, many studies provide limited mechanistic insight into observed performance, often lacking detailed analysis of electron‐transfer kinetics, interfacial processes, or mass‐transport effects. This limits the generalizability of reported findings and impedes the development of rational design principles. Addressing these challenges will require more systematic studies, advanced and standardized characterization approaches, and harmonized reporting practices to enable meaningful cross‐study comparison.

## 5 Advances in MXene‐Based Electrochemical Sensors

The rapid expansion of MXene research has led to a wide range of electrochemical sensing platforms targeting analytes of environmental, biomedical, and industrial relevance. While numerous studies have demonstrated the potential of MXenes for improving sensor performance, the diversity of reported systems often results in a fragmented understanding, where individual works are presented in isolation without clear links to underlying design principles. In light of the mechanistic framework established in the previous section (Section 4), it is therefore essential to reinterpret these applications by identifying common material strategies, dominant sensing mechanisms, and recurring performance limitations. In this section, recent advances in MXene‐based electrochemical sensors are discussed by grouping analytes into broader functional categories rather than treating each system independently. This approach enables a more coherent analysis of how MXene properties—particularly conductivity, surface chemistry, and structural organization—govern sensing behavior across different applications. Particular emphasis is placed on correlating material design with electrochemical performance, highlighting the roles of MXenes as conductive scaffolds, catalytic interfaces, and adsorption platforms.

### 5.1 Small Molecules and Metabolites

Electrochemical detection of small molecules and metabolites, including glucose, hydrogen peroxide (H_2_O_2_), and nitrite (NO_2_^−^), represents one of the most extensively investigated application domains for MXene‐based sensors. These analytes are central to clinical diagnostics, environmental monitoring, and food safety, and their well‐defined redox behavior makes them ideal systems for probing structure–property–performance relationships in MXene materials. When reconsidered through the mechanistic framework outlined earlier, it becomes evident that MXenes do not merely act as passive conductive supports, but function as multifunctional interfaces that simultaneously regulate electron‐transfer kinetics, catalytic activity, and analyte adsorption. H_2_O_2_ sensing is a particularly illustrative case, as H_2_O_2_ serves both as a direct analyte and as an intermediate in enzymatic reactions. Pristine Ti_3_C_2_T_*x*_ MXene electrodes have demonstrated notable intrinsic electrocatalytic activity toward H_2_O_2_ reduction and oxidation, with reported detection limits reaching the nanomolar range, in some cases as low as 0.7 nM [172]. This performance is closely associated with surface terminations and exposed transition metal sites, which promote analyte adsorption and reduce activation barriers for redox reactions. However, the most significant enhancements are consistently observed in hybrid architectures. For example, Ti_3_C_2_T_*x*_/Pt nanoparticle composites have achieved detection limits of 448 nM, where Pt provides highly active catalytic centers, while MXenes ensure efficient electron transport across the interface [173]. Similarly, TiO_2_–Ti_3_C_2_ hybrids exhibit detection limits as low as 14 nM, illustrating how oxide integration can modulate catalytic pathways and improve stability [174]. More complex architectures, such as porphyrin‐MOFs@MXene systems, have enabled real‐time monitoring of H_2_O_2_ released from living cells, demonstrating the transition of MXene‐based sensors from model systems toward biologically relevant applications [175]. Collectively, these examples show that MXenes primarily function as conductive scaffolds that stabilize catalytic or recognition components while maintaining rapid charge transport, a recurring design principle in small‐molecule sensing. A comparative overview of MXene‐based H_2_O_2_ sensing platforms, including material composition, detection limits, and linear ranges, is summarized in Table 2, highlighting the performance gains enabled by hybridization strategies.

**TABLE 2:** Performance summary of representative MXene‐based H_2_O_2_ sensors.

Sensor configuration	Measurement type	Target molecule	LOD	Linear range	Response time	Matrix	Observations/remarks	Ref.
NiCo_2_O_4_/Ti_3_C_2_T_*x*_ heterointerface‐modified electrode	Electrochemical (amperometry, CV)	H_2_O_2_	0.21 μM	0.001–7.5 mM	∼2 s (fast amperometric response)	Real samples: milk and tap water	Heterointerface engineering enhances electron transfer; high sensitivity (≈326 μA/mM/cm^2^); excellent stability and reproducibility; recoveries 95%–104% in real samples	[176]
Ti_3_C_2_T_*x*_/PtNP/GCE	CV	H_2_O_2_	448 nM	490 μM–53.6 mM	—	—	PtNPs enhance sensitivity	[173]
Ti_3_C_2_T_*x*_/GCE	CV	H_2_O_2_	0.7 nM	—	—	—	Bare MXene‐based detection	[172]
NiO/Ti_3_C_2_T_*x*_	CV	H_2_O_2_	0.34 μM	0.01–4.5 mM	—	—	Porous microsphere structure increases electroactive sites	[177]
Porphyrin‐MOFs@ Ti_3_C_2_ MXenes on ITO electrode	CV, SWV, CA	H_2_O_2_	3.1 µM	10 µM–3 mM (2 ranges)	Immediate	PBS buffer, HeLa cells	Coordination bond improves dispersion, electrocatalytic activity, and stability; enabled real‐time monitoring of cell‐secreted H_2_O_2_ with high selectivity and reproducibility	[175]
CuO‐CeO_2_/Ti_3_C_2_T_*x*_ MXene on GCE	CV, CA, EIS, i‐t	H_2_O_2_	1.67 μM	5.0–100 μM	Continuous, stable	PBS, human serum	CuO‐CeO_2_ provides high catalytic activity; MXene enhances conductivity; exhibits excellent stability, reproducibility, and selectivity; 97.3%–104% recovery in serum; chronoamperometric electrolysis rate = 0.61 × 10^3^ M^−1^s^−1^; theoretical mechanism supported by DFT.	[178]
Nafion/Hb/Ti_3_C_2_/GCE	CV	H_2_O_2_	20 nM	0.1–260 μM	—	—	Good electrocatalytic activity from Hb	[179]
PB/Ti_3_C_2_	CV	H_2_O_2_	0.2 µM	0.6 μM–63.6 μM and 63.6 μM–254 μM	—	Cell culture (fibroblast)	Intercalated PB NPs showed biocompatibility and catalysis for real‐time detection	[180]

**FIGURE 8:**
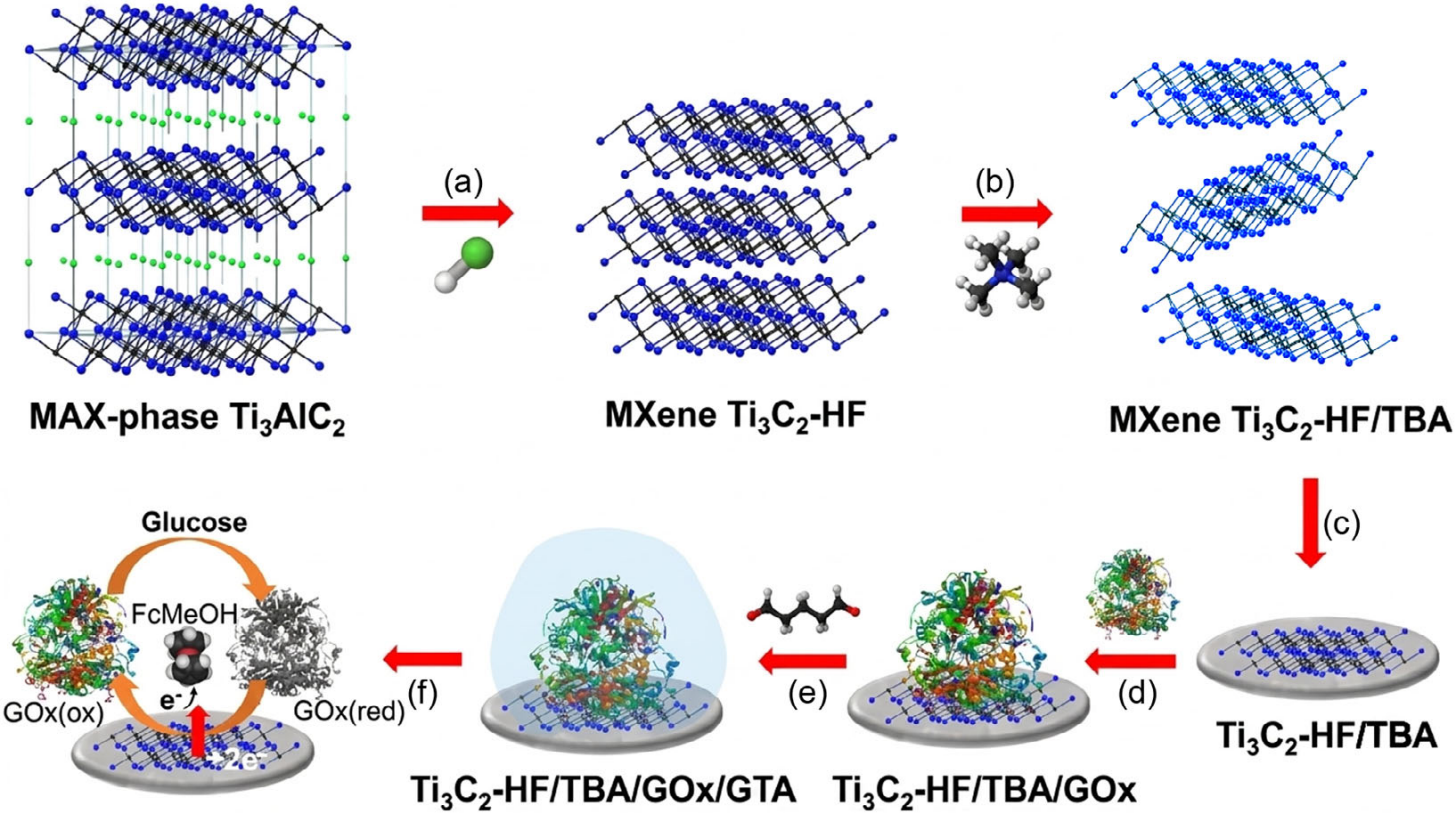
Stepwise demonstration of the fabrication and functionalization of a Ti_3_C_2_‐based glucose biosensor: (a) Exfoliation of Ti_3_AlC_2_ using HF acid to produce Ti_3_C_2_‐HF. (b) Delamination of Ti_3_C_2_‐HF with tetrabutylammonium hydroxide (TBAOH) to yield Ti_3_C_2_‐HF/TBA. (c) Modification of a glassy carbon electrode with Ti_3_C_2_‐HF/TBA. (d) Immobilization of glucose oxidase (GOx) onto the modified surface. (e) Cross‐linking of GOx with glutaraldehyde (GTA). (f) Mechanism of glucose detection via the constructed biosensing platform [181]. Copyright 2020. Reproduced with permission from the American Chemical Society.

Glucose sensing further highlights the dual functional role of MXenes, particularly when comparing enzymatic and non‐enzymatic approaches. In enzymatic systems, MXenes provide a hydrophilic and functionally active surface that supports dense and stable immobilization of glucose oxidase (GOx), while facilitating electron transfer between the enzyme active site and the electrode. A representative example is the Ti_3_C_2_‐HF/TBA/GOx/GTA biosensor, which exhibited a detection limit of 23 μM and a wide linear range extending beyond 27 mM (Figure 8) [181]. Electrochemical analysis showed that delaminated MXene structures significantly reduce peak‐to‐peak separation, indicating enhanced heterogeneous electron‐transfer kinetics, while glutaraldehyde crosslinking improves enzyme immobilization stability and increases signal intensity. The resulting biosensor also demonstrated excellent selectivity against common interferents such as ascorbic acid, dopamine, and uric acid, underscoring the importance of precise interfacial engineering in enzymatic MXene‐based sensing platforms [181]. At the same time, non‐enzymatic glucose sensors based on MXene composites have emerged as a robust alternative, addressing the intrinsic instability of enzyme‐based platforms. For example, MXene/NiCo‐LDH and NiO‐decorated MXene systems exhibit detection limits in the submicromolar range and strong resistance to interference, owing to their intrinsic electrocatalytic activity toward glucose oxidation [182, 183]. In another approach, Nb_2_CT_*x*_ decorated with selenium NPs enables detection over a wide concentration range (2–30 mM), demonstrating the adaptability of MXene‐based systems for physiologically relevant monitoring [184]. More complex configurations, such as paper‐based MXene sensors integrated with enzymatic recognition elements, have achieved extremely low detection limits while enabling dual‐analyte (glucose and uric acid) detection in biofluids [185]. Across these studies, a clear functional distinction emerges: Enzymatic systems primarily exploit MXene surface chemistry for biorecognition and enzyme immobilization, whereas non‐enzymatic systems rely on MXenes as conductive and electrocatalytic scaffolds, often in synergy with transition metal oxides or nanostructured catalysts. This distinction is further reflected in the performance metrics summarized in Table 3, where enzymatic and non‐enzymatic platforms exhibit different trade‐offs in sensitivity, linear range, and operational stability depending on their design strategy.

**TABLE 3:** Representative MXene‐based glucose electrochemical sensors.

Sensor configuration	Measurement type	Target molecule	LOD	Linear range	Response time	Matrix	Observations/Remarks	Ref.
Ti_3_C_2_T_*x*_ MXene sheets decorated with Pt NPs (nanozyme‐based dual‐mode platform)	Colorimetric (UV–vis absorbance with TMB oxidation) and electrochemical (chronoamperometry at 0.7 V vs Ag/AgCl)	Glucose	∼1.37 μM (colorimetric, model‐derived)	0.01–12 mM (simulated bulk concentration range)	∼80 s (transient H_2_O_2_ response delay in pseudo‐2D model; >40 min incubation noted experimentally for colorimetric assays)	Physiological glucose concentrations (1–10 mM range emphasized)	Pseudo‐2D modeling shows increased effective *K*_m_ (≈4.8 mM vs intrinsic 2.5 mM) and decreased *V*_max_ (≈20% lower); heterogeneity of Pt clusters thickens diffusion layer and causes premature saturation; excellent agreement with experimental UV–vis spectra	[186]
Ti_3_C_2_T_*x*_ MXene (etched from Ti_3_AlC_2_) decorated with AuNPs, PPy, chitosan, and GOx (MAPCG nanocomposite)	Electrochemical (DPV, CV, amperometry)	Glucose	4.23 μM	0.02–13 mM	1–2 s (steady‐state amperometric response)	Human serum (validated against commercial glucometer)	High sensitivity (110.6 μA/mM/cm^2^); excellent selectivity against UA, AA, DA, H_2_O_2_; reproducibility (RSD = 1.64%); long‐term stability (91% signal retention after 30 days); recovery rates 94%–98.7% in serum	[187]
Fe_3_O_4_‐ Ti_3_C_2_T_*x*_ modified electrode	Electrochemical (CV, DPV, amperometry)	Glucose	0.25 μM	0.001–10 mM	∼3 s (fast amperometric response)	Human serum samples	High sensitivity (≈178 μA/mM/cm^2^); strong anti‐interference against AA, UA, DA; reproducibility (RSD < 3%); stability (92% signal retention after 20 days); recovery rates 95%–102% in serum	[188]
Flexible Janus fabric patch with Ti_3_C_2_T_*x*_ MXene‐based electrode, enzyme immobilization (GOx for glucose, LOx for lactate)	Electrochemical (amperometry, chronoamperometry)	Glucose, lactate	Glucose: 0.16 mM; lactate: 0.12 mM	Glucose: 0.2–5.0 mM; Lactate: 0.2–5.0 mM	∼3–5 s (rapid amperometric response)	Human sweat (tested during exercise)	Janus fabric enables directional sweat transport, preventing evaporation and contamination; high selectivity against UA, AA, DA; stable performance under continuous sweating; recovery rates 95%–102% in sweat samples	[189]
Fe_3_O_4_‐ Ti_3_C_2_T_*x*_ modified electrode	Electrochemical (CV, DPV, amperometry)	Glucose	0.25 μM	0.001–10 mM	∼3 s (fast amperometric response)	Human serum samples	High sensitivity (≈178 μA/mM/cm^2^); strong anti‐interference against AA, UA, DA; reproducibility (RSD < 3%); stability (92% signal retention after 20 days); recovery rates 95%–102% in serum	[190]
AuTNs/ Ti_3_C_2_ MXene on GCE	CV, DPV, CA	Glucose	1.43 nM	0.1 nM–10 mM	1.0 s	0.1 M NaOH	Tapered Au nanostructures on MXene provide high surface area and enhanced charge transfer, showing high sensitivity and stability for glucose detection	[191]
PGOx polynanogel@ Ti_3_C_2_T_*x*_ MXene/chitosan on GCE	CV, DPV, EIS	Glucose	3.1 μM	0.03–16.5 mM	2.2 s (avg.)	Human serum (10× diluted)	High sensitivity (48.98 μA·mM^−1^·cm^−2^), superior repeatability (RSD 1.31%), reproducibility (RSD 1.83%), stability (85.8% signal retained after 200 cycles), and real‐time applicability in clinical samples	[192]
Ti_3_C_2_‐HF/TBA/GOx/GTA	CV	Glucose	23 μM	50–27 750 μM	—	—	GOx immobilized on MXene	[181]
Au@CuO/LIG (laser‐induced graphene) with V_2_CT_*x*_ MXene (unstable)	CA, CV, EIS	Glucose	1.8 μM	0.005–5.0 mM	1.5 s	NaOH solution, human urine	LIG enabled low‐cost, disposable sensor platform; V_2_CT_*x*_ MXene enhanced charge transfer but degraded quickly; best stability and performance obtained with Au@CuO only; recovery in urine: 96.8%–102.95%; sensitivity 1.124 × 10^6 ^μA/mM·cm^2^	[193]
Ti_3_C_2_T_*x*_ MXene/CoNiMn‐LDH modified GCE	CV, CA	Glucose	0.24 μM	10–900 μM	∼5–12 s	0.1 M NaOH	Hierarchical structure improves conductivity and charge transport. Excellent selectivity over DA, AA, UA, Fru, H_2_O_2_, NaCl. Shows good biocompatibility (~80% viability at 100 µM); suitable for wearable sensing applications	[194]
NiO NPs decorated PANI NS/Ti_3_C_2_T_*x*_ (NiOMP) on SPCE	CV, DPV	Glucose	0.019μM	5–500 μM	—	Artificial sweat, human sweat	Highly sensitive and selective enzyme‐free glucose sensor for non‐invasive sweat analysis; validated on human sweat samples over 5 days	[183]
MXene‐Ti_3_C_2_/GOD	CV	Glucose	1.96 μM	39.8 μM–1.319 mM	—	—	GOD used for enzymatic detection	[195]
Ti_3_C_2_ MXene/NiCo‐LDH/GCE	CV	Glucose	0.53 μM	2 μM–4.096 mM	—	—	NiCo‐LDH enhances electrocatalytic properties	[182]
Ti_3_C_2_/Cu_2_O	Photoelectrochemical	Glucose	0.17 nM	0.5 nM–0.5 mM	—	Buffer	Excellent PEC performance under light, first MXene PEC sensor	[196]
Ti_3_C_2_T_*x*_‐MXene/Co@NC nanocomposite on GCE	CV, chronoamperometry	Glucose	66.8 nM	0.5–100 µM	—	Human blood (spiked)	Co NPs confined in N‐doped carbon improve catalytic activity; MXene boosts electron transfer. Exhibits high sensitivity (370 µA/mM·cm^2^), reproducibility (RSD 5.6%), and stability (91.9% after 10 days). Recoveries: 107%, 92.7%, 94.2%. Outperforms many Co‐based sensors	[197]
Paper‐based Cu‐TCPP(Fe)/Ti_3_C_2_ MXene with GOD/UO	CV, EIS	Glucose, Uric Acid	1.88 aM /5.80 pM	0.001 nM–5 mM/0.025 nM–5 mM	—	Artificial sweat, urine, saliva	Transient paper‐based sensor with excellent electrocatalytic performance; real‐time dual‐channel detection; cost‐effective, non‐invasive; stable for 100 days; recovery > 94% with RSD < 10%; strong biocompatibility; superior to most existing glucose and UA sensors; highly applicable to POC and wearable diagnostics	[185]

**FIGURE 9:**
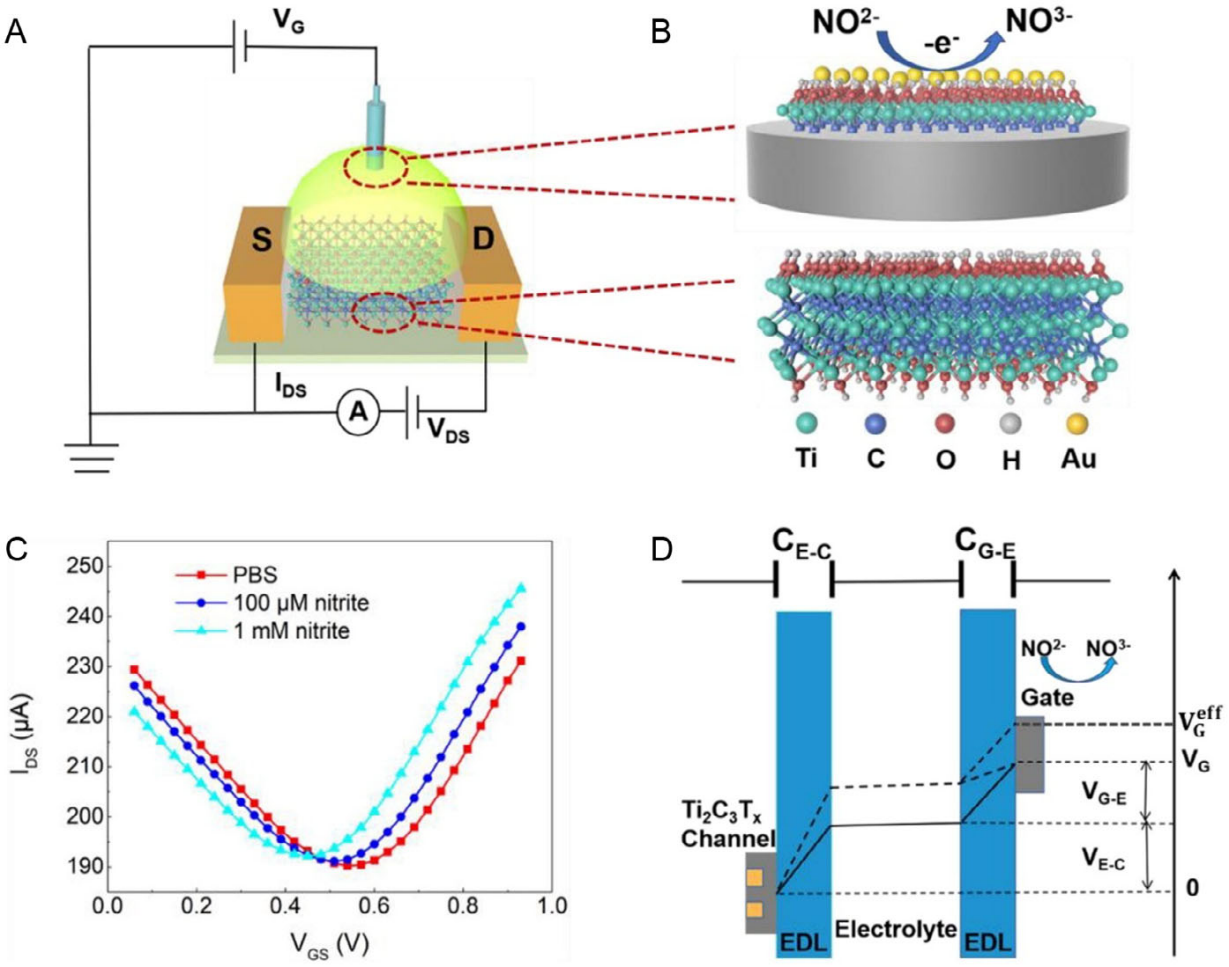
(A) Schematic illustration of the Ti_3_C_2_T_*x*_‐based electrochemical transistor. (B) Depiction of the chemical interaction occurring at the gate surface and the layered structure of Ti_3_C_2_T_*x*_ nanosheets. (C) Transfer characteristics (*I*_DS_ vs. *V*_GS_, *V*_DS_ = 0.05 V) under varying nitrite concentrations in PBS. (D) Simulated potential distribution across the transistor before and after nitrite exposure [199]. Copyright 2023. Reproduced with permission from Elsevier.

Nitrite detection provides a complementary perspective, emphasizing the importance of interfacial adsorption and electron transfer in systems dominated by direct electrochemical oxidation. MXene‐based composites such as Au@carbon quantum dot–MXene electrodes have achieved detection limits as low as 0.078 μM with wide linear ranges extending to the millimolar regime, benefiting from synergistic interactions between MXenes, gold NPs, and carbon nanostructures [198]. More significantly, device‐level innovations have further enhanced sensitivity. A Ti_3_C_2_T_*x*_‐based electrochemical transistor incorporating Au NPs demonstrated an exceptionally low detection limit of 10 pM, with dual linear ranges spanning nanomolar to millimolar concentrations (Figure 9) [199]. In this architecture, the MXene channel enables efficient modulation of charge transport through electrical double‐layer formation, while Au nanoparticle‐functionalized gate regions enhance analyte adsorption and signal amplification [199]. These results highlight that MXenes can function not only as catalytic or conductive materials, but also as active components in transistor‐based sensing architectures, enabling sensing modalities beyond conventional voltammetric approaches. A detailed comparison of MXene‐based nitrite sensors, including device configurations and analytical performance, is provided in Table 4, underscoring the advantages of device‐level integration for achieving ultra‐low detection limits.

**TABLE 4:** MXene‐based electrochemical sensors for nitrite detection.

Sensor configuration	Measurement type	Target molecule	LOD	Linear range	Response time	Matrix	Observations/Remarks	Ref.
Ti_3_C_2_T_*x*_ MXene decorated with Cu–Ag NPs (TCX‐AgCu) on SPCE and GCE	Electrochemical (CV, SWV, EIS)	Nitrate (NO_3_^−^), Nitrite (NO_2_^−^)	NO_3_^−^: 5.2 μM (low range), 124 μM (high range); NO_2_^−^: 0.031 μM (low range), 0.211 μM (high range)	NO_3_^−^: 0.1–10 mM and 10–100 mM; NO_2_^−^: 0.1–1 mM and 1–30 mM	∼30 s accumulation time; electrochemical response immediate	Soil water, lake water, tap water	Dual detection of NO_3_^−^ and NO_2_^−^; strong selectivity against interfering ions; reproducibility (RSD ≈ 1.9%–3.5%); stability over 100 CV cycles; recoveries 85.6%–94.8% in real samples	[200]
Ti_3_C_2_T_*x*_ MXene combined with MoS_2_ nanosheets (MoS_2_‐ Ti_3_C_2_T_*x*_ composite) on SPCE	Electrochemical (CV, DPV, amperometry)	Nitrite (NO_2_^−^), Caffeine	NO_2_^−^: 0.12 μM; Caffeine: 0.08 μM	NO_2_^−^: 0.5–120 μM; Caffeine: 0.2–100 μM	∼5 s (fast amperometric response)	Real samples: tap water, soft drinks, tea	Dual‐analyte detection possible without crosstalk; high selectivity against common interferents; reproducibility (RSD < 3%); recoveries 95%–104% in real samples; synergistic effect of MoS_2_ and MXene enhances conductivity and catalytic activity	[201]
Au@CQDs‐ Ti_3_C_2_T_*x*_ MXene nanocomposite on GCE	DPV, CV, EIS	Nitrite (NO_2_^−^)	0.078 µM	1–500 µM and 500–3200 µM	—	Tap water, salted vegetables	CQDs reduce AuNPs in a green synthesis. Composite provides enhanced electrocatalysis and wide‐range detection. Demonstrated stability, low detection limit, and good performance in real food matrices	[198]
Ti_3_C_2_T_*x*_ MXene transistor with AuNPs/Ti_3_C_2_T_*x*_ on gate	Transfer curves, CV, i‐t	Nitrite	0.01 nM	0.01 nM–0.5 μM and 0.5 μM–5 mM	Rapid; stable > 15 days	PBS, lake water	High sensitivity due to Ti_3_C_2_T_*x*_ channel and AuNPs‐enhanced gate; good selectivity, time stability (82% after 15 days); recoveries in lake water 97.8%–106.7%; outperforms traditional materials like graphene or MoS_2_	[199]

Collectively, these small‐molecule sensing systems reveal a consistent and unifying design strategy. MXenes operate as multifunctional platforms that integrate three key roles: (i) conductive pathways that accelerate electron transfer, (ii) catalytic or catalyst‐supporting interfaces that lower reaction barriers, and (iii) adsorption‐active surfaces that preconcentrate analytes at the electrode interface. Hybridization with metals, oxides, MOFs, or polymers further amplifies these functions, enabling high sensitivity and rapid response across a broad range of analytes. Despite these advances, several limitations remain. Enzymatic systems continue to suffer from instability due to enzyme denaturation and environmental sensitivity, which limits long‐term applicability. Non‐enzymatic systems, while more robust, often face selectivity challenges in complex matrices containing multiple electroactive species. In addition, MXene oxidation in aqueous environments can gradually reduce conductivity and alter surface chemistry, leading to signal drift over time. These challenges highlight the need for improved surface stabilization strategies, antifouling designs, and standardized fabrication protocols. Addressing these issues will be essential for translating MXene‐based small‐molecule sensors from highly sensitive laboratory demonstrations into reliable real‐world analytical platforms. Overall, the trends observed across glucose, H_2_O_2_, and nitrite sensing (summarized in the preceding tables) underscore the central role of MXene hybridization and interface engineering in achieving both high sensitivity and broad applicability.

### 5.2 Environmental Pollutants

The detection of environmental pollutants using MXene‐based electrochemical sensors reflects a shift from model analytes toward chemically complex and structurally diverse targets, including heavy metal ions, pesticides, and industrial contaminants. Unlike small metabolites, these analytes often exhibit weak or indirect electrochemical activity, requiring sensor architectures that emphasize preconcentration, selective binding, and signal amplification. Within this context, MXenes function not only as conductive substrates but also as chemically active interfaces capable of coordinating, adsorbing, or catalytically transforming target species. The resulting performance is therefore governed by the interplay between surface chemistry, hybrid material design, and device configuration.

**FIGURE 10:**
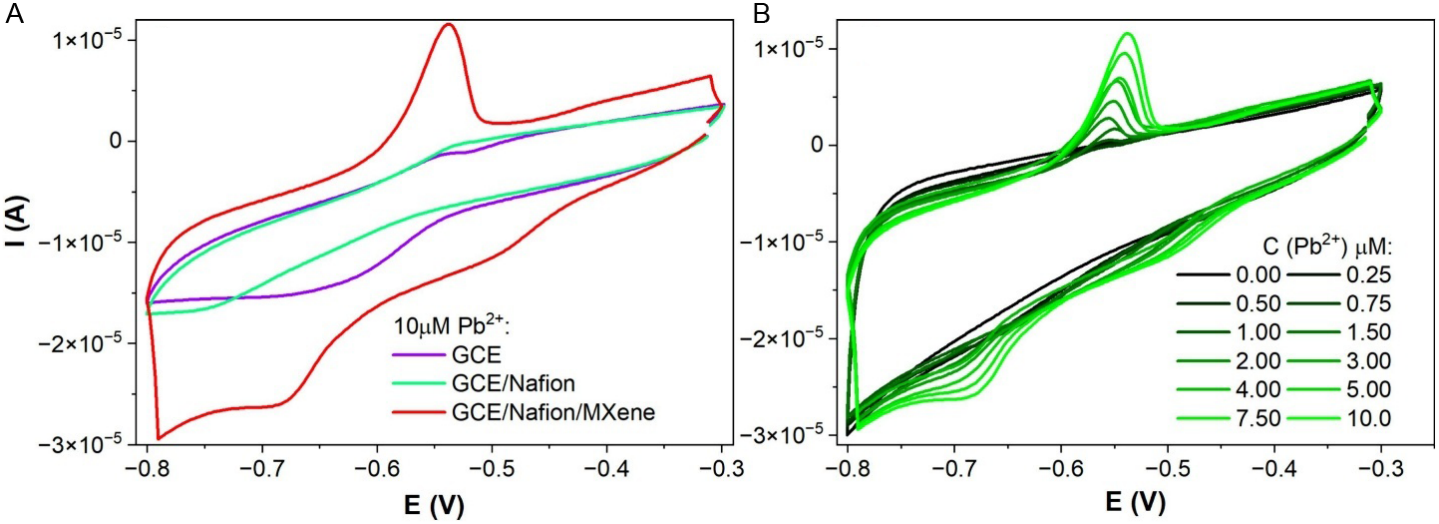
Electrochemical behavior of MXene‐modified electrodes for Pb^2+^ detection. (A) Cyclic voltammograms comparing bare GCE, Nafion‐coated GCE, and GCE/MXene/Nafion electrodes in 10 μM Pb^2+^ solution. The MXene‐modified system exhibits a pronounced anodic peak (~−550 mV) and enhanced current response, confirming its superior redox activity and ion adsorption capacity. (B) CV profiles of GCE/MXene/Nafion electrode at increasing Pb^2+^ concentrations (0–10 μM), showing progressive amplification of the anodic (~−550 mV) and cathodic (~−700 mV) peaks, while the oxygen‐related cathodic peak (~−500 mV) remains unaffected, highlighting selective Pb^2+^ responsiveness [202]. Copyright 2024. Reproduced with permission from Elsevier.

**FIGURE 11:**
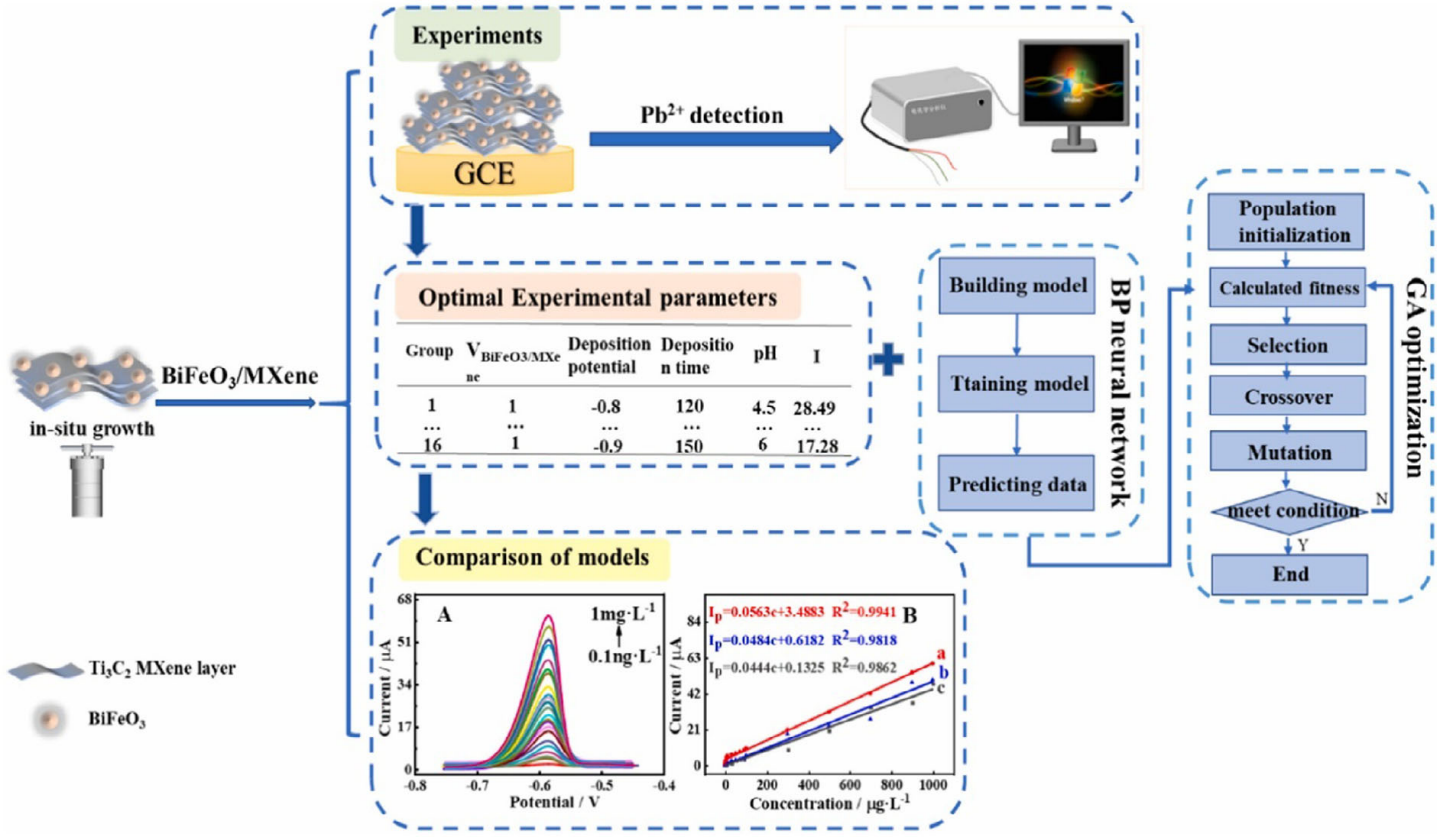
Electrochemical sensor integrating BiFeO_3_/Ti_3_C_2_ MXene and machine learning for fast, straightforward, and ultra‐sensitive lead ion detection [203]. Copyright 2023. Reproduced with permission from Elsevier.

Heavy metal ion detection represents one of the most mature application areas. The abundance of surface functional groups on MXenes, including hydroxyl, oxygen, and fluorine terminations, enables strong coordination with metal ions, facilitating their preconcentration at the electrode interface prior to electrochemical readout. This is particularly advantageous in stripping voltammetry‐based techniques, where sensitivity is directly linked to accumulation efficiency. For example, Ti_3_C_2_T_*x*_ MXene‐modified electrodes have demonstrated highly sensitive detection of Pb^2+^, with detection limits reaching the low nanomolar range due to enhanced adsorption and accelerated electron‐transfer kinetics (Figure 10) [202]. The integration of secondary nanostructures further amplifies this performance. Bismuth nanoparticle/MXene composite electrodes enable sensitive Pb^2+^ detection through synergistic adsorption and redox processes at the hybrid interface, achieving detection limits down to ~ 0.1 ng/L (Figure 11) [203]. Similarly, thiol‐functionalized bismuth metal–organic framework (Bi‐MOF–SH)/MXene sandwich architectures provide abundant binding sites and high conductivity, resulting in ultra‐sensitive Pb^2+^ detection with a LOD of 0.012 μg/L and excellent reproducibility [204]. In addition, gold (Au) nanoparticle‐decorated MXene systems exploit the affinity of Au toward multiple heavy metal ions (Cd^2+^, Pb^2+^, Cu^2+^, and Hg^2+^) while simultaneously enhancing electron‐transfer efficiency, thereby improving both sensitivity and selectivity [205]. Beyond noble metal‐based hybrids, alkaline‐intercalated MXene electrodes have been developed as mercury‐free alternatives, enabling reliable multi‐ion detection with wide linear ranges (0.1–1.5 μM) and low detection limits (32–130 nM) [206]. Collectively, these examples highlight a recurring design principle in heavy metal sensing: MXenes serve as both adsorption‐active platforms and conductive matrices that integrate or stabilize secondary catalytic and affinity‐enhancing components. The performance of these systems, including detection limits and multi‐ion capabilities, is summarized in Table 5, where the influence of surface functionalization and composite engineering on metal‐ion affinity becomes evident.

**TABLE 5:** Selected MXene‐based electrochemical sensors for heavy metal ion detection.

Sensor configuration	Measurement type	Target molecule	LOD	Linear range	Response time	Matrix	Observations/Remarks	Ref.
Ti_3_C_2_T_*x*_ MXene combined with calcinated MOF (Ti_3_C_2_T_*x*_ @CMOF composite) on GCE	Electrochemical (DPV, CV, EIS)	Pb^2+^ (lead ions)	0.12 μM	0.5–100 μM	Not explicitly stated	Tap water samples	Ti_3_C_2_T_*x*_@CMOF shows synergistic effect: high conductivity of MXene + porous structure of CMOF; excellent selectivity against Cd^2+^, Hg^2+^, Cu^2+^; recoveries 96%–103% in tap water	[207]
Ti_3_C_2_T_*x*_ MXene combined with VS_2_ nanosheets (Ti_3_C_2_T_*x*_‐VS_2_ nanocomposite) on GCE	Electrochemical (DPV, CV, EIS)	Pb^2+^ (lead ions)	0.017 μM	0.05–100 μM	∼2 s (fast amperometric response)	Tap water, river water	Synergistic effect of MXene conductivity and VS_2_ catalytic activity; high selectivity against Cd^2+^, Hg^2+^, Cu^2+^; reproducibility (RSD < 3%); stability (≈92% signal retention after 20 days); recoveries 95%–103% in real water samples	[208]
Glassy carbon electrode (GCE) modified with Au@ Ti_3_C_2_T_*x*_ MXene and dual aptamer probes; DNAzyme release strategy for signal amplification	Electrochemical (DPV, CV, EIS; dual‐signal readout)	Pb^2+^, Cd^2+^	Pb^2+^: 0.003 nM; Cd^2+^: 0.004 nM	Pb^2+^: 0.01–100 nM; Cd^2+^: 0.01–100 nM	∼5 s (rapid electrochemical response)	Tap water, lake water, fruit juice	Dual‐signal design improves accuracy and reduces false positives; DNAzyme release amplifies signal; excellent selectivity against other ions; reproducibility (RSD < 3%); recoveries 95%–104% in real samples	[209]
Glassy carbon electrode (GCE) modified with V_2_CT_*x*_ MXene nanosheets	Electrochemical (SWASV, CV, EIS)	Cd^2+^, Pb^2+^	Cd^2+^: 2.28 μg/L; Pb^2+^: 2.40 μg/L (individual); Cd^2+^: 2.71 μg/L; Pb^2+^: 2.83 μg/L (simultaneous)	5–50 μg/L (both ions)	Deposition time optimized at 6 min; stripping response immediate	Tap water, mineral water	Distinct peaks at −0.8 V (Cd^2+^) and −0.6 V (Pb^2+^) allow simultaneous detection without crosstalk; reproducibility RSD < 3.5%; recoveries 103%–108% in real samples; stability maintained for 2 weeks, slight oxidation after 4 weeks	[210]
Ti_3_C_2_T_*x*_ MXene integrated with ZIF‐67 MOF and 18‐crown‐6 ether (Crown@ZIF‐67/MXene composite) on SPCE	Electrochemical (SWASV, CV, EIS)	Tl^+^ (thallium ion)	0.036 μg/L	0.1–300 μg/L (two‐stage linearity)	Deposition time optimized at 120 s; stripping response immediate	Tap water, Yangtze River water, Minzhu Lake water, zinc‐oxide‐plant wastewater	Crown ether provides size‐specific Tl^+^ recognition; ZIF‐67 offers porous enrichment; MXene ensures conductivity and antifouling; excellent selectivity against Pb^2+^, Cd^2+^, Cu^2+^; reproducibility RSD ≈ 3%; stability after 500 CV cycles; recoveries 78%–115% depending on matrix; close agreement with ICP‐MS results	[211]
Delaminated Ti_3_C_2_T_*x*_ MXene	CV	Pb^2+^	48.7 nM	0.25–10 μM (linear up to ∼1 μM)	—	DI water, tap water, lake water	Sensor exhibited a reversible Pb^2+^/PbO redox mechanism at MXene surface; oxygen‐dependent response observed at −500 mV; showed strong selectivity over Co^2+^, Fe^2+^, Al^3+^, Mn^2+^, and Zn^2+^; usable in unfiltered water with adjusted LOD	[202]
Layered N‐doped carbon/Ti_3_C_2_T_*x*_ MXene on GCE	SWV, CV, EIS	Cu^2+^, Hg^2+^	Cu^2+^: 0.0196 µM; Hg^2+^: 0.056 µM	Cu^2+^: 0.5–10 µM; Hg^2+^: 1–10 µM	Deposition: 400 s	Water, milk	Nitrogen‐rich MXene‐based heterostructure synthesized using deep eutectic solvents; excellent selectivity, low LOD, enhanced conductivity; suitable for food and environmental analysis	[212]
Ti_3_C_2_@N‐doped carbon/GCE	SWASV	Cd^2+^ and Pb^2+^	Cd^2+^: 2.55 nM; Pb^2+^: 1.10 nM	Cd^2+^: 0.1–4.0 µM; Pb^2+^: 0.05–2.0 µM	Deposition: 360 s	Seawater, tap water	Nitrogen‐doped carbon improves conductivity and provides selective binding sites. The sensor shows excellent selectivity, stability, and reproducibility. Pb^2+^ has stronger response than Cd^2+^ due to competitive adsorption. The sensor performs well even in the presence of 10x interfering ions	[213]
3D Ti_3_C_2_T_*x*_ MXene‐AuNPs@MBA(EAMTD/TGA) on multi‐electrode ITO array	Individual & simultaneous	Pb^2+^, Cu^2+^, Hg^2+^	Pb^2+^: 0.07 μg/L; Cu^2+^: 0.13 μg/L; Hg^2+^: 0.21 μg/L	1–1300 μg/L	—	Grain (sorghum, maize, wheat, rice)	Sensor uses SH‐functionalized AuNPs on 3D MXene to enhance selectivity and conductivity. Excellent reproducibility (RSD < 5%), high anti‐interference, recoveries from 91.4 to 110.93% vs. ICP‐MS	[205]
BiFeO_3_/Ti_3_C_2_ MXene‐modified GCE	Current signal	Pb^2+^	0.1 ng/L	0. 1 ng/L–3 mg/L	—	Lake water	OED‐ML optimization (BPANN + GA) provided better accuracy and sensitivity; showed excellent repeatability, stability (91.05% after 15 d), and selectivity	[203]
Alkalized Ti_3_C_2_ MXene on pencil graphite electrode (PGE)	SWASV, CV, EIS	Cd^2+^ and Pb^2+^ (simultaneous)	Cd^2+^: 8.10 × 10^−6 ^mg/L; Pb^2+^: 9.12 × 10^−6 ^mg/L	Cd^2+^: 0.0005–0.07 mg/L; Pb^2+^: 0.0002–0.08 mg/L	250 s deposition	Spiked scallops (digested)	Cost‐effective, reusable PGE; excellent selectivity and stability (90% retention after 7 days); RSD < 5%; dual metal detection with no interference; good match with ICP‐MS results; improved interfacial charge transfer via ‐OH rich MXene surface; wide range and low LODs	[214]
DL‐Ti_3_C_2_ MXene	DPV + NIR photothermal	Hg^2+^	0.43 nM (photo); 17.2 pM (DPV)	1 nM ‐ 50 µM (photo); 50 pM–500 nM (DPV)	2 hr reaction + 5 min irradiation	Tap water (spiked)	Hg^2+^ specifically binds Ti atoms at MXene edges as HgClOH. Dual signal decreases after etching. High selectivity vs. 15 metal ions, high reproducibility (RSD ∼4.75%–6.4%), good stability. Detection below WHO limits. Real sample recovery 96.9%–105%. Self‐validation improves reliability	[215]
3D porous AuNPs/ Ti_3_C_2_T_*x*_ MXene/GCE with aptamer and Nile Blue	DPV, CV, Fluorescence	Hg^2+^	2.69 fM (EC), 1.60 fM (FL)	10 fM‐0.1 nM (EC), 5–100 fM (FL)	20 min incubation	Environmental water samples (Nanhu Lake, Clear Lake)	Dual‐signal aptasensor using “turn‐on” EC and “turn‐off” FL modes; high sensitivity, excellent specificity to Hg^2+^, recoveries 98.2%–105.0% (EC), 97.0–106.0% (FL); aptamer bound to AuNPs via Au‐S; 3D MXene templated with PMMA improved surface area and performance	[216]
Fe‐MOF/Ti_3_C_2_T_*x*_ MXene modified GCE	SWASV	As(III)	0.58 ng/L	1–10 and 10–100 ng/L	Deposition: 600 s	Tap water, lake water, river water	MXene enhances conductivity and Fe‐MOF provides As(III) coordination. Dual synergistic effects offer high surface area and bonding affinity. Strong peak current, excellent selectivity (minimal interference from Pb^2+^, Cd^2+^, Cu^2+^, etc.), and good repeatability (RSD ∼2%). High recovery (97.4%–98.4%) in real samples	[217]
Bi‐MOF‐SH/Ti_3_C_2_T_*x*_ MXene/Bi‐MOF‐SH multilayer on GCE	DPASV, CV, EIS	Pb^2+^	0.012 μg/L	0.03–20.0 μg/L	—	Tap water	Excellent conductivity and sensitivity from thiol‐MOF and MXene synergy; durable and selective; reproducibility (RSD < 3%); real water recovery 97%–103%. Strong anti‐interference with minimal cross‐talk from other cations	[204]
Ti_3_C_2_T_*x*_/MWCNTs	SWASV	Cu^2+^, Zn^2+^	Zn^2+^: 1.5 ppb, Cu^2+^: 0.1 ppb	Zn^2+^: 350–830 ppb, Cu^2+^: 10–600 ppb	—	Human biofluids (urine, sweat)	Flexible, wearable sensor with LbL assembly for noninvasive detection	[218]
Ti_3_C_2_/MWCNTs on Au/PET	SWASV	Cu^2+^, Zn^2+^	Cu 0.1 ppb; Zn 1.5 ppb	Cu 10–500 ppb; Zn 200–600 ppb	—	Buffer	Flexible sensor showed low bending effect and suitable for portable devices	[218]
Nb_4_C_3_T_*x*_ MXene on GCE	SWASV, EIS	Pb^2+^	12 nM	0.025–0.5 mM	—	Tap and bottled water	Large interlayer spacing improves selectivity and recovery, good reproducibility	[219]
Ti_3_C_2_T_*x*_/alkaline intercalated MXene (Alk‐Ti_3_C_2_)	SWASV	Cu^2+^, Hg^2+^, Cd^2+^, Pb^2+^	Cu^2+^: 32 nM, Hg^2+^: 130 nM, Cd^2+^: 98 nM, Pb^2+^: 41 nM	For all: 0.1–1.5 μM	—	—	Simultaneous detection with well‐separated peaks, good selectivity, reproducibility over 3 weeks	[206]
3D MGMA (melamine‐doped rGO/Ti_3_C_2_T_*x*_ MXene) on SPCE	DPASV, CV, EIS	Zn^2+^, Cd^2+^, Pb^2+^	0.44 μg/L (Zn), 0.33 μg/L (Cd), 0.28 μg/L (Pb)	1–1000 μg/L (individually), 3–900 μg/L (simultaneously)	∼200 s deposition	Grain (corn, wheat, rice, sorghum), Water (tap, river)	High sensitivity and selectivity; synergistic effects from melamine's amino groups and rGO/MXene conductivity; excellent reproducibility and interference resistance; results validated against ICP‐MS. Porous aerogel structure enhances adsorption and electron transfer. Stability RSD < 1% over 10 cycles	[220]
Ti_3_C_2_T_*x*_ MXene aerogel‐CuO/carbon cloth (MXA‐CuO/CC)	DPASV	Cd^2+^ and Pb^2+^	Cd^2+^: 0.3 μg/LPb^2+^: 0.2 μg/L	Cd^2+^: 4–800 μg/LPb^2+^: 4–1200 μg/L	—	Grain, water (tap, lake)	CuO and Bi^3+^ synergize with MXene to enhance adsorption and stripping signals. High selectivity and reproducibility (RSD < 5.3%), excellent recovery (93%–111.5%). Comparable with ICP‐MS and AAS results; stable and reusable electrode design	[221]
UiO‐66‐NH_2_‐ Ti_3_C_2_T_*x*_ MXene@rGO on GCE	DPASV	Cd^2+^ and Pb^2+^	Cd^2+^: 0.46 ppb; Pb^2+^: 0.40 ppb	Cd^2+^: 2–800 ppb; Pb^2+^: 1–600 ppb	—	Grain samples (rice, corn, wheat, sorghum), water	UiO‐66‐NH_2_ adds amino‐binding sites; MXene@rGO increases conductivity and surface area. Excellent stability (RSD < 5%), recovery (92.4%–109.8%), and anti‐interference. Signal unaffected by mutual ion presence	[222]
Ti_3_C_2_T_*x*_ MXene‐NH_2_@CeFe‐MOF‐NH_2_ on GCE	CV, DPV, EIS	Cd^2+^, Pb^2+^, Hg^2+^	0.47, 0.32, 0.39 nM	0.5–300 nM (all ions)	—	River water, tap water	High sensitivity due to amino‐functionalized MXene‐MOFs; strong binding affinity and signal amplification; capable of simultaneous detection; real sample recovery rates between 96.7% and 104.7% with RSD < 4.2%	[223]

**FIGURE 12:**
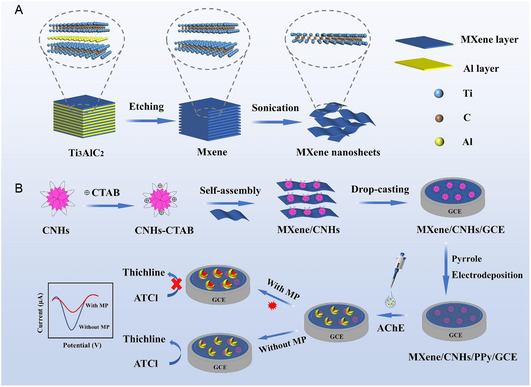
(A) Synthesis steps of MXene nanosheets. (B) Sequential formation of the MXene/CNHs/PPy nanocomposite and the construction workflow of the AChE‐functionalized electrochemical sensor on a glassy carbon electrode for methyl parathion sensing [225]. Copyright 2024. Reproduced with permission from Elsevier.

Pesticide detection introduces additional complexity, as many pesticide molecules are not inherently electroactive and therefore require indirect detection strategies. In such systems, MXenes are frequently integrated with enzymes, MIPs, or nanozymes to enable selective recognition and signal transduction. A widely studied approach is based on acetylcholinesterase (AChE)‐functionalized MXene sensors for organophosphate pesticide detection, where enzymatic inhibition is converted into an electrochemical response. In a representative system, MXene–chitosan composites were used to immobilize AChE, leading to improved enzyme stability and enhanced signal response due to the high surface area and hydrophilic nature of the MXene matrix [224]. Beyond enzymatic platforms, MXenes have also been incorporated into nanozyme‐based architectures. For example, MXene integrated with bimetallic nanozyme systems enables indirect pesticide detection through enzyme‐mimicking catalytic reactions, where catalytic activity replaces biological recognition elements [225]. This configuration provides a high‐surface‐area conductive platform that facilitates efficient electron transfer while enabling selective detection of non‐electroactive targets via catalytic signal amplification (Figure 12) [225]. Across these systems, the sensing performance is primarily governed by the efficiency of interfacial immobilization and the accessibility of active sites, both of which are strongly enhanced by the layered morphology and surface chemistry of MXenes. As summarized in Table 6, enzymatic and non‐enzymatic MXene‐based pesticide sensors exhibit distinct performance profiles: Enzyme‐based systems typically offer superior selectivity, whereas MIP‐ and nanozyme‐based architectures provide improved operational stability and environmental robustness.

**TABLE 6:** MXene‐based electrochemical sensors for pesticide detection.

Sensor configuration	Measurement type	Target molecule	LOD	Linear Range	Response time	Matrix	Observations/Remarks	Ref.
Glassy carbon electrode (GCE) modified with Nd_2_WO_6_‐ Ti_3_C_2_T_*x*_ composite	Electrochemical (DPV, CV, EIS)	Carbendazim (fungicide)	0.014 μM	0.05–100 μM	∼3 s (fast amperometric response)	Real samples: apple, grape, orange, tap water	High sensitivity and selectivity; strong anti‐interference against other pesticides; reproducibility (RSD < 3%); recoveries 95%–104% in fruits and water; stability (≈90% signal retention after 20 days)	[226]
Ti_3_C_2_T_*x*_ MXene decorated with Au NPs (MXene‐Au) combined with Au‐Ce‐COF with antibody immobilization (MXene‐Au‐MB‐PTM probe) on GCE immunosensor	Electrochemical (CV, DPV, EIS)	Methyl parathion (PTM)	1.58 ng/mL	0.00475–9.5 μg/mL	Incubation ∼120 min; electrochemical response immediate	Real water samples (river, tap water)	Dual signal amplification (MXene‐Au probe + Au‐Ce‐COF substrate); high selectivity against other pesticides and metal ions; stability > 80% after 7 days at 4°C; recoveries 92.7%–100.2% (river water), 105.8%–110.9% (tap water)	[227]
Ti_3_C_2_T_*x*_ MXene combined with graphitic carbon nitride (g‐C_3_N_4_/MXene composite) on GCE	Electrochemical (DPV, CV, EIS)	Carbaryl	0.009 μM	0.05–100 μM	∼3 s (fast amperometric response)	Real food samples: apple, grape, tomato	High sensitivity and selectivity; strong anti‐interference against other pesticides; reproducibility (RSD < 3%); stability (≈90% signal retention after 20 days); recoveries 94%–105% in fruit samples	[228]
Ti_3_C_2_T_*x*_ MXene/CNHs/PPy/AChE on GCE	DPV, CV, EIS	Methyl parathion	0.00021 ng/mL	0.002–346 ng/mL	15 min	Cabbage, soil, water	Multidimensional nanocomposite enhances conductivity, enzyme immobilization, and sensitivity; exhibits high stability and specificity. Linear relation with inhibition rate; low Michaelis–Menten constant (*K*_m_ = 1.68 mM); validated with GC–MS in real samples. RSD < 5%.	[225]
Ti_3_C_2_T_*x*_ MXene + AuPt NPs + AChE/chitosan on GCE	DPV, CV, EIS	Chlorpyrifos	1.55 pg/mL	10^−8^–10^−3^ mg/mL	15 min incubation	Apple and cabbage extracts	High stability (83% after 30 days), good anti‐interference, low RSD (3.9%–5.1%), real sample recovery 95%–103%, wide range, biocompatible platform. Excellent synergy between MXene and AuPt for enhanced conductivity and enzyme activity retention.	[224]
Ag@TiO_2_/ Ti_3_C_2_T_*x*_ MXene on SGGT (graphene FET)	DPV, CV, EIS + SGGT	Paraquat	0.02 nM	0.1 nM–10 μM	10 min total	PBS (pH 8), spring and groundwater	Excellent anti‐interference against ions and organophosphates. Strong catalytic activity and PQ selectivity due to Ag and TiO_2_ synergy on MXene. High accuracy in real samples (recovery 97.5%–105.1%). SGGT provided superior sensitivity vs. traditional DPV	[229]
InP/ZnSeS quantum dots/ Ti_3_C_2_T_*x*_ MXene on CP	Oxidation peak current (µA)	Carbendazim (CBZ)	14.6 nM	0.019–527.26 µM	—	Industrial wastewater, orange juice	Biocompatible composite showed high selectivity, reproducibility (±4.17%), and stability (97.4% after 3 weeks); in vitro and in vivo safe up to 200 nM	[230]
Ti_3_C_2_T_*x*_ MXene@MB/PtNPs on GCE (MB = methylene blue)	DPV, CV, EIS	Carbendazim (CBZ)	4.2 ng/mL	10 ng/mL–30 µg/mL	∼30 min prep/sample	Lettuce, grape, tomato	MB serves as internal reference; PtNPs enhance conductivity; excellent selectivity, reproducibility (RSD < 4.6%), stable for 7 days; successful vegetable sample recovery	[231]
Ti_3_Al_(1−x)_C_2_–OH_(x)_(TAC‐1) ‐Sm_2_Sn_2_O_7_ nanocomposites on SPCE	DPV	Diquat (DQ)	2.73 μM	0.5–1.5 μM	—	Tap water, river water, bell pepper	MXene functionalized with Sm_2_Sn_2_O_7_ enhanced surface area and charge transfer; sensor showed high selectivity and reproducibility in detecting DQ in real food and water samples	[232]
Colorimetric: QCT‐AgNPs; electrochemical: AgNPs/Ti_3_C_2_(OH)_0.8_F_1.2_ MXene on SPGE	DPV, CV (electrochemistry), ImageJ (colorimetric)	Carbaryl (CBR)	1.79 µM (colorimetric), 0.01 µM (electrochemical)	5–50 µM (colorimetric), 0–60 µM (electrochemical)	∼30 min (colorimetry incubation)	Orange fruit extract	Smartphone‐compatible μPAD platform combining colorimetric and electrochemical zones; effective real‐sample testing; excellent selectivity, stability; recovery 104%–117%, RSD < 10%	[233]
Ti_2_C_2_/CNHs	CV	Carbendazim	1.0 nM	3.0 nM to 10.0 μM	—	Buffer	CNHs acted as spacer to prevent restacking of MXene and enhance conductivity	[234]

**FIGURE 13:**
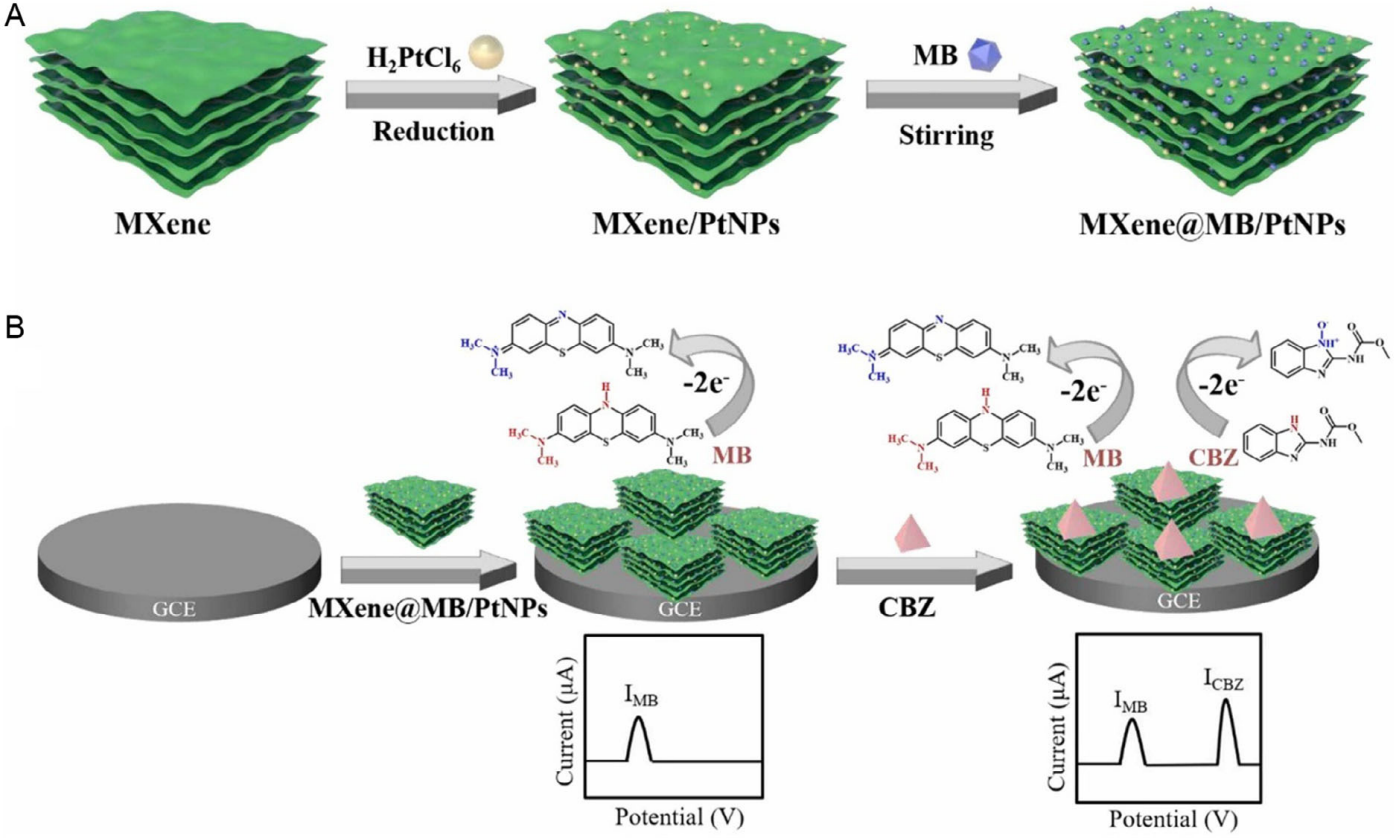
(A) Stepwise synthesis of Ti_3_C_2_T_*x*_ MXene@MB/PtNPs nanocomposites. (B) Their integration into a ratiometric electrochemical platform for carbendazim detection [231]. Copyright 2025. Reproduced with permission from Elsevier.

Ratiometric sensing strategies have also been applied to carbendazim detection, where MXene composites are engineered to incorporate internal reference probes or quantum dots. In one system, methylene blue‐functionalized Ti_3_C_2_T_*x*_ MXene/Pt NPs provided both enhanced conductivity and a stable reference signal (Figure 13) [231], while another approach employed MXene–quantum dot hybrids to generate dual‐signal outputs, thereby improving accuracy, reproducibility, and sensitivity in pesticide residue analysis [230]. In parallel, MXene‐based nanozyme composites, including those incorporating transition metal oxides or metal–organic frameworks, enable catalytic detection of pesticide residues through redox‐mediated signal amplification (Table 6). These systems generally offer improved stability compared to enzymatic sensors; however, achieving high selectivity and minimizing matrix effects in complex environmental samples remains a persistent challenge.

**FIGURE 14:**
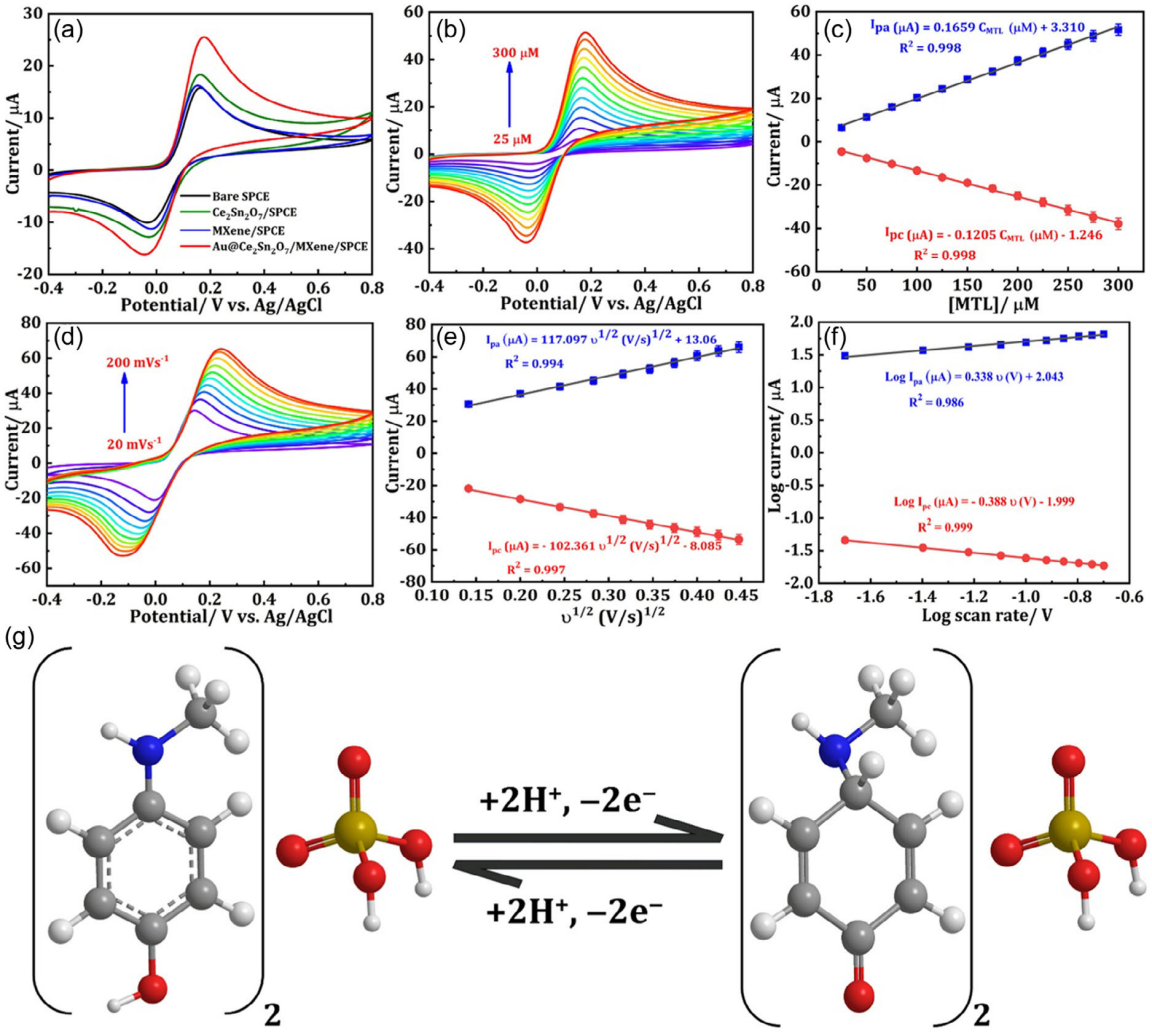
Electrochemical characterization of Au@Ce_2_Sn_2_O_7_/MXene‐modified SPCE for MTL detection. (a) CV spectra comparing bare SPCE, Ce_2_Sn_2_O_7_/SPCE, MXene/SPCE, and Au@Ce_2_Sn_2_O_7_/MXene/SPCE in 75 μM MTL, showing enhanced peak current with the hybrid composite. (b) CV responses of Au@Ce_2_Sn_2_O_7_/MXene/SPCE at increasing MTL concentrations (25–300 μM), indicating concentration‐dependent signal amplification. (c) Calibration plots of MTL concentration versus anodic and cathodic peak currents, demonstrating excellent linearity. (d) CV curves at varying scan rates (20–200 mV/s) in 75 μM MTL, showing increased peak currents with scan rate. (e) Linear plots of √scan rate versus peak current, confirming diffusion‐controlled behavior. (f) Log–log plots of scan rate versus peak current, with slopes near 0.5, supporting reversible electron transfer. (g) Proposed redox mechanism of MTL involving two‐electron, two‐proton exchange [237]. Copyright 2024. Reproduced with permission from Elsevier.

Beyond heavy metals and pesticides, MXene‐based sensors have also been applied to the detection of industrial pollutants such as phenolic compounds and nitroaromatic species. These analytes typically undergo direct electrochemical oxidation or reduction; however, their detection is often hindered by electrode fouling, surface passivation, and interference from coexisting species. To address these limitations, MXene composites incorporating carbon nanostructures or conductive polymers have been widely employed to enhance surface stability, suppress fouling, and facilitate rapid electron transfer. A comparison of MXene‐based sensors for phenolic compounds is summarized in Table 7, highlighting the role of hybrid conductive architectures in improving both sensitivity and operational stability. For example, a MXene‐based composite sensor for phenolic pollutants was developed through the incorporation of conductive carbon nanostructures, which stabilized MXene layers and mitigated fouling during repeated measurements. The system exhibited well‐resolved voltammetric peaks for hydroquinone and catechol, demonstrating improved selectivity and signal stability [245]. Further advances have been achieved using hierarchical hybrid architectures. A bifunctional MXene/carbon black/ZIF‐8 “honeycomb” sandwich structure enabled simultaneous detection of hydroquinone (HQ) and catechol (CC), where MXene provided conductivity, carbon black regulated interlayer spacing, and ZIF‐8 contributed abundant adsorption sites. This synergistic configuration resulted in wide linear ranges (HQ: 0.3–160 μM; CC: 0.5–165 μM) and low detection limits (HQ: 12.6 μM; CC: 82 nM). The sensing mechanism was attributed to a two‐electron/two‐proton redox process governing HQ and CC oxidation [246]. In addition, bio‐derived carbonized composite systems have emerged as sustainable alternatives. A microbial‐derived antimony sulfide/carbonized electrode for catechol and hydroquinone detection demonstrated that pyrolytic conversion of biogenic Sb_2_S_3_ into a porous, defect‐rich carbon‐supported electrocatalyst significantly enhances adsorption‐controlled redox processes [237]. Chronoamperometric analysis further confirmed increased electroactive surface area and improved adsorption capacity compared to bare electrodes (Figure 14), highlighting the role of defect engineering and porous carbon frameworks in improving pollutant sensing performance [237].

**TABLE 7:** MXene‐based electrochemical sensors for phenolic compound detection.

Sensor configuration	Measurement type	Target molecule	LOD	Linear range	Response time	Matrix	Observations/remarks	Ref.
Ti_3_C_2_T_*x*_ MXene decorated with Au NPs (AuNPs/MXene composite) on GCE	Electrochemical (DPV, CV, EIS)	Luteolin	0.002 μM	0.01–100 μM	∼3 s (fast amperometric response)	Real samples: herbal extracts, food matrices	AuNPs enhance catalytic activity and electron transfer; MXene provides conductivity and large surface area; high selectivity against other flavonoids; reproducibility (RSD < 3%); stability (≈90% signal retention after 20 days); recoveries 95%–104% in real samples	[235]
Ti_3_C_2_T_*x*_ MXene combined with CuCoSe_2_ (p–n heterojunction composite) on GCE	Electrochemical (CV, DPV, EIS) with ML‐assisted signal processing	Rutin (flavonoid phenolic)	0.0012 μM	0.005–100 μM	∼2.5 s (fast amperometric response)	Real samples: herbal extracts, pharmaceutical formulations	p–n heterojunction enhances charge separation and electron transfer; machine learning improves signal discrimination and accuracy; high selectivity against other flavonoids and phenolics; reproducibility (RSD < 3%); recoveries 94%–106% in real samples	[236]
D‐FeCu‐MOF/N, S‐ Ti_3_C_2_ QD@rGO on GCE	DPV, CV, EIS	Catechol	0.0014 µM	0.004–520 µM	∼12 min total	Tap water, river water	High stability (89.7% retention after 28 days), low RSD (<1%), strong anti‐interference, reproducibility, large electroactive area (34.47 cm^2^), dual‐electron/proton redox, synergistic enhancement from defect‐MOF, rGO, and N, S‐doped MXene QDs. Recovery: 98.23%–102.67%	[82]
Au@Ce_2_Sn_2_O_7_/ Ti_3_C_2_ MXene on SPCE	CV, DPV, EIS	Metol	0.00563 µM	0.00125–1021.96 µM	—	Tap, lake, river, pond water; human serum, urine	Highly sensitive and selective detection of metol. Sensor shows excellent stability (96.7%), reproducibility (RSD 1.01%), and storage retention (89.1% after 9 days). Excellent recovery in real samples. Synergistic combination of Ce_2_Sn_2_O_7_, MXene, and AuNPs provides superior electrocatalytic performance	[237]
Ti_3_C_2_T_*x*_ MXene/GCE	CV	H_2_S	16 nM	0.1–300 μM	—	—	High sensitivity toward H_2_S	[238]
AuNPs@N‐Ti_3_C_2_ MXene on GCE	CV, DPV	Sudan I	2.57 nM	0.01–10 µM	∼6 min incubation	Sugus candy extract (pH 7 PBS, 30% acetone)	High surface area from wrinkled morphology; improved hydrophilicity and electrocatalytic activity due to N‐doping and AuNPs. Good selectivity vs. colorants (ascorbic acid, sunset yellow, etc.). Recoveries 94.3%–96.2%, comparable to UPLC‐MS. Stable and highly reproducible	[239]
MIP/ Ti_3_C_2_T_*x*_ MXene/ZIF‐67‐modified GCE	DPV, CV, EIS	Ethyl carbamate	0.405 µg/L	8.9–44,500 µg/L	10 min	Baijiu	MIP provides specificity, MXene improves conductivity, ZIF‐67 catalyzes H_2_O_2_; excellent selectivity and anti‐interference; results match GC–MS analysis	[240]
Ti_3_C_2_ MXene‐tyrosinase biosensor	CV, DPV	Phenol	12 nM	0.05 to 15.5 μM	Fast	Real water samples	High sensitivity, mediator‐free system exploiting MXene's high surface area and conductivity	[103]
MIP/pTHi/ Ti_3_C_2_T_*x*_ MXene/Fe@Ti‐MOF‐NH_2_ on GCE	SWV, CV, EIS	Catechol	0.54 µM	1–300 µM and 300–4000 µM	3 min elution, 10 min incubation	Tap water, human urine	Dual‐signal ratiometric MIP sensor; pTHi used as internal reference; strong anti‐interference; high reproducibility (RSD < 2%), long‐term stability (>96% after 4 weeks); recoveries of 96.5%–101.5% in real samples; imprinting factor = 8.45; superior to most previous CC sensors	[241]
FeCu‐MOF‐919/Ti_3_C_2_T_*x*_ MXene on GCE	DPV, CV, EIS	Resorcinol	0.08 µM	0.5–152.5 µM	5 min incubation (total < 10 min)	Tap water (spiked)	Excellent sensitivity (0.23 µA µM^−1^ cm^−2^), reproducibility (RSD 1.47%), high stability (71.5% after 7 days), strong anti‐interference vs. CC, HQ, BPA. Superior to previously reported RS sensors. Mechanism involves 2H^+^/2e^−^ redox. Recovery in tap water: 96.25%–103.37%	[242]
MXene (Ti_3_C_2_T_*x*_) + V_2_O_5_ on GCE + Nafion	CV, DPV, EIS	Bisphenol A	87 nM	411 nM–31.2 µM	—	0.1 M phosphate buffer (pH ‐)	Good repeatability and antifouling effect due to V_2_O_5_. Linear current increase with concentration. Stability over 28 days confirmed. Less resistivity than Ti_3_AlC_2_ or bare Ti_3_C_2_T_*x*_. Suitable for environmental monitoring	[243]
TiO_2_‐ Ti_3_C_2_T_*x*_ MXene/MWCNTs/PDA on GCE	CV	Xanthin	0.018 µM	0.1–100 0.018 µM	30 hr incubation	HFF‐1 cell culture	Evaluated cytotoxicity by detecting purine metabolism (X/G, A/HX) in HFF‐1 cells. Stronger sensitivity than MTT assay; detects IC50 for 9,10‐PQ (4.44 µM), 1,2‐NQ (67.72 µM), 1,4‐BQ (291.88 µM). Sensor integrates multiple active materials for enhanced electron transfer and purine capture. Good selectivity, reproducibility, and signal stability over 16 days (96% retention)	[244]
Ti_3_C_2_ MXene‐AgBiS_2_ nanocomposite on GCE	CV, DPV	4‐Nitrophenol	0.00254 µM	0.02–1869 µM	—	Tap water, PBS	Composite shows strong synergy, enhanced charge transfer, high selectivity vs. similar nitrophenols and ions, good reproducibility (RSD 2.21%), recovery 95%–101.3% in tap water, optimal pH 7.0. Sensor stable, reliable for real‐world monitoring	[245]
Ti_3_C_2_T_*x*_ MXene/CB/ZIF‐8 modified GCE	DPV, CV, EIS	Hydroquinone (HQ), catechol (CC)	0.0126 µM (HQ)0.0082 µM (CC)	HQ: 0.3–160.0 µMCC: 0.5–165.0 µM	—	Environmental water (Dongdagou river)	Excellent simultaneous detection; honeycomb sandwich morphology enhances conductivity and sensitivity	[246]
VSe_2_@V_2_O_3_@V_2_CT_*x*_ MXene on GCE	DPV, CV, EIS	Hydroquinone (HQ), catechol (CC)	HQ: 0.144 µM, CC: 0.131 µM	0.5–600 µM (both HQ and CC)	—	Drinking water, beverages	Multiphase MXene nanocomposite with CQD intercalation improves conductivity, active sites, and selectivity; successful simultaneous detection in real samples	[247]
alk‐Ti_3_C_2_/N‐PC	CV	Hydroquinone, catechol	4.8 nM, 3.1 nM	0.5 µM–150 µM	—	Industrial wastewater	Alkalization enhanced hydrogen bonding interaction for improved sensing	[248]
Anti‐AFB1/NH_2_‐Mo‐MOF/Ti_3_C_2_ on SPE	CV, EIS	Aflatoxin B1	8.0 pg/mL	0.06–50 ng/mL	10 min	Water, pistachio extract	2D‐2D nanocomposite (MOF/MXene) with covalent antibody conjugation showed excellent sensitivity, specificity (RSD 2.32%), reproducibility, 14‐week stability, and selectivity even in complex matrices; validated against HPLC and LC‐MS with recovery rates > 97%	[249]
Nafion/RLM/Au@ Ti_3_C_2_T_*x*_ MXene on GCE	I‐t (amperometry), CV, EIS	Aflatoxin B1	2.8 nM	0.01–50 µM	∼80 s	PBS (pH 7.4), corn samples	Uses rat liver microsomes for metabolic conversion of AFB1 to AFM1; stable (96.1% current after 24 h); selective vs. OTA, ZEN, AFM1;recovery in corn samples 71.4%–87.3%; first MXene‐liver microsome biosensor reported; outperforms UPLC‐MS/MS in some metrics	[250]
SL‐Ti_3_C_2_T_*x*_ MXene/GCE (calcined at 700°C)	Amperometric	H_2_S	5.77 nM	0.1–500 µM	Immediate	PBS (0.1 M)	Calcination at 700°C removed ‐F and ‐OH groups, enhanced TiO_2_ formation, improved conductivity. Sensor shows high selectivity, repeatability, and > 90% stability over 28 days	[251]

A broader perspective across these pollutant classes reveals a consistent trend toward hybridization and functional integration. MXenes provide a versatile platform that can be tailored through compositional and structural modifications to address the specific challenges associated with different analyte types. For heavy metals, the dominant mechanism is adsorption and preconcentration; for pesticides, selective recognition through enzymatic, imprinted, or nanozyme‐based interfaces; and for industrial pollutants, catalytic activity combined with antifouling performance. In each case, the role of MXenes extends beyond simple conductivity enhancement to encompass interfacial chemistry and surface engineering that directly govern sensing behavior. Nevertheless, the translation of these systems from laboratory demonstrations to practical environmental monitoring remains limited by several factors. Matrix effects in real samples, including competing ions and natural organic matter, can significantly degrade sensitivity and selectivity. Long‐term stability is another critical issue, particularly due to the susceptibility of MXenes to oxidation under ambient and aqueous conditions. In addition, challenges related to reproducibility and scalable fabrication hinder meaningful comparison across studies and slow progress toward standardized performance benchmarks. Addressing these limitations will require standardized testing protocols, robust surface stabilization strategies, and device architectures capable of maintaining reliable performance in complex real‐world environments.

### 5.3 Biomolecules and Clinical Targets

The extension of MXene‐based electrochemical sensing toward biomolecules and clinically relevant targets represents a critical step in translating material‐level advantages into biomedical applications. In contrast to small molecules and environmental analytes, biomolecular detection imposes significantly stricter requirements in terms of selectivity, biocompatibility, and operation in complex physiological matrices. Proteins, nucleic acids, and disease biomarkers often exist at ultralow concentrations in the presence of numerous interfering species, necessitating sensor designs that integrate highly specific recognition elements with efficient signal transduction. Within this context, MXenes serve as multifunctional platforms that not only facilitate rapid electron transfer but also provide chemically tunable surfaces for immobilizing biological recognition units.

**FIGURE 15:**
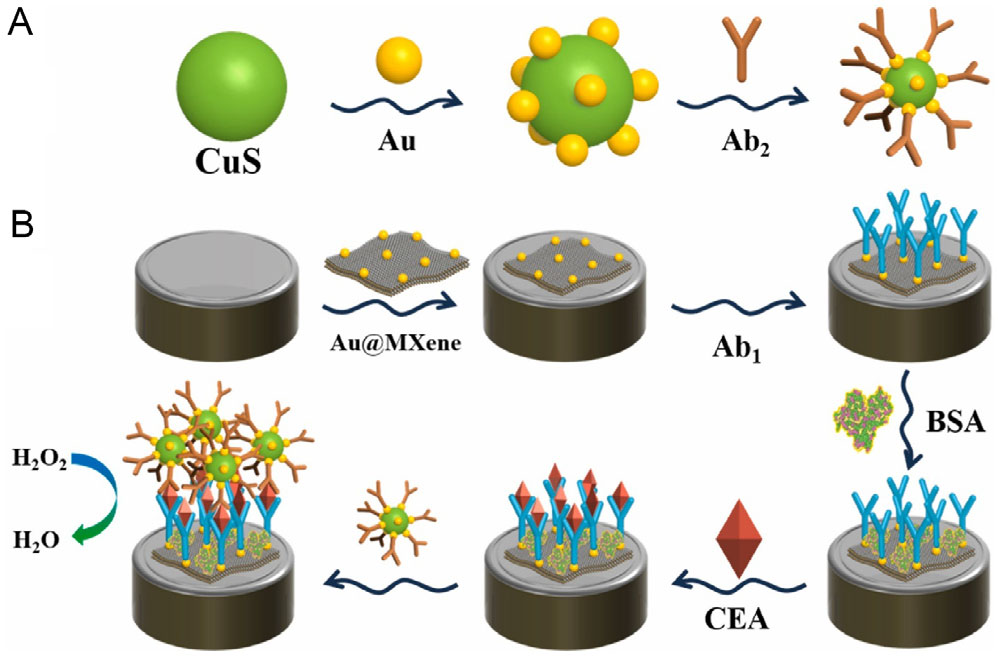
(A) Fabrication process of Au@CuS‐Ab_2_ nanocomposite: CuS NPs are synthesized and decorated with Au NPs via in situ reduction, followed by conjugation with secondary antibodies (Ab_2_). (B) Stepwise assembly of the sandwich‐type electrochemical immunosensor: Au@MXene is drop‐cast onto a glassy carbon electrode (GCE) and functionalized with primary antibodies (Ab_1_). After blocking with BSA, CEA and Au@CuS‐Ab_2_ are sequentially immobilized. The resulting sensor enables sensitive detection of CEA via H_2_O_2_‐mediated electrochemical signal amplification [252]. Copyright 2025. Reproduced with permission from Elsevier.

Protein detection has been widely explored using MXene‐based immunosensors and affinity platforms. The large surface area and hydrophilic nature of MXenes enable high antibody loading while preserving bioactivity, a key requirement for achieving high sensitivity. For example, Ti_3_C_2_T_*x*_‐based immunosensors have been developed for carcinoembryonic antigen (CEA) detection, achieving picomolar detection limits through the integration of MXenes with metal NPs and enzyme labels (Figure 15) [252]. In these systems, MXenes function as conductive scaffolds that accelerate electron transfer between the recognition layer and the electrode while simultaneously stabilizing immobilized antibodies via surface functional groups [252]. The incorporation of AuNPs further enhances performance by promoting biomolecule immobilization through Au–S interactions and providing catalytic signal amplification. The performance of MXene‐based biomarker detection systems, including sensitivity and assay configurations, is summarized in Table 8, underscoring the effectiveness of hybrid nanostructures in clinical sensing applications.

**TABLE 8:** MXene‐based electrochemical sensors for biomarker detection.

Sensor configuration	Measurement type	Target molecule	LOD	Linear range	Response time	Matrix	Observations/Remarks	Ref.
α‐Fe_2_O_3_/ Ti_3_C_2_T_*x*_ MXene heterostructure electrode with RAFT polymerization amplification	Electrochemical (DPV, EIS)	microRNA‐21	0.33 fM	1 fM–1 nM	Not explicitly stated	Human serum samples	Photo‐Fenton catalysis enhances RAFT polymerization, yielding ultrasensitive detection; strong stability and reproducibility; recovery rates in serum 95%–104%	[253]
Ti_3_C_2_T_*x*_ MXene decorated with AgNPs (Ti_3_C_2_‐AgNPs nanocomposite) SPE integrated into dual‐chamber 3D‐printed PDMS microfluidics	Electrochemical (CV, SWV, EIS)	PSA and PSMA (prostate cancer biomarkers)	PSA: 0.045 ng/mL; PSMA: 0.041 ng/mL	0.1–1000 ng/mL (both biomarkers)	∼30 min incubation at 37°C	Human serum samples (clinical validation)	Dual‐channel design eliminates signal crosstalk; high sensitivity and specificity; repeatability (RSD < 2%); stability (≈84%–90% signal retention after 12 days); strong concordance with ELISA results; potential for point‐of‐care testing	[254]
Ti_3_C_2_T_*x*_ MXene decorated with Au nanoclusters (AuNCs/MXene composite) on GCE	Electrochemical (DPV, CV, EIS)	Guanine, adenine	Guanine: 0.002 μM; Adenine: 0.003 μM	Guanine: 0.01–100 μM; Adenine: 0.01–100 μM	∼3 s (fast amperometric response)	Real samples: fish DNA, human serum	High sensitivity and selectivity; strong anti‐interference against cytosine, thymine, uric acid, ascorbic acid; reproducibility (RSD < 3%); recoveries 95%–104% in DNA and serum samples	[255]
Glassy carbon electrode (GCE) modified with C@MXene‐Hemin	Electrochemical (DPV, CV, EIS)	Homocysteine (Hcy)	0.12 μM	0.5–200 μM	∼4 s (fast amperometric response)	Human serum samples	3D carbon coating prevents MXene restacking, increases surface area; hemin provides catalytic activity; high selectivity against cysteine, glutathione, methionine; reproducibility (RSD < 3%); stability (≈90% signal retention after 20 days); recoveries 95%–104% in serum	[256]
Crumpled Ti_3_C_2_T_*x*_ MXene nanosheets GCE	Electrochemical (CV, DPV, EIS, chronoamperometry)	Ascorbic acid (AA), dopamine (DA), uric acid (UA), acetaminophen (ACA)	AA: 0.8 µM; DA: 0.6 µM; UA: 0.7 µM; ACA: 0.9 µM	10–200 µM (all analytes)	1.47–2.38 s (steady‐state response, simulation + experiment)	Biological and food matrices (PBS buffer, simulated fluids)	Crumpled morphology increases surface area (150 m^2^/g) and exposes ‐OH/‐O groups; π‐π stacking (DA) and H‐bonding (AA, UA, ACA) drive selectivity; competitive adsorption causes only 5%–8% sensitivity loss; reproducibility confirmed; minimal interference (<2.5%) from glucose/citric acid	[257]
DCNC@AgNPs/ Ti_3_C_2_ MXene nanocomposite on GCE	DPV, CV	Apolipoprotein A‐1	2.20 fg/mL	0.01–1000 pg/mL	45 min incubation	Human serum (diluted)	Combines dialdehyde CNC‐stabilized AgNPs with MXene for enhanced signal; strong specificity vs. other proteins (Muc‐1, HSA, VEGF, etc.); 90% signal retained after 30 days at 4°C; RSD 1.2%; recovery in real serum: 97.87%–104.65%; 168% signal increase over bare GCE; optimized at pH 7.4 and 1:15 NC ratio.	[258]
Ti_3_C_2_ MXene‐derived Na‐titanate + CNT on SPCE	DPV, CV, EIS	BNP (B‐type natriuretic peptide)	1.75 pg/mL	31.3–8000 pg/mL	4 h incubation	Human serum (diluted 10×)	M‐NTO‐CNT nanocomposite improved conductivity and surface area. High selectivity and specificity. Outperformed ELISA. Suitable for early diagnosis of acute heart failure. Stable over 18 days (87% retention). Recovery rates ∼97%–99%. Excellent R^2^ of 0.996. Proof of personalized medicine potential	[259]
Ti_3_C_2_T_*x*_ MXene‐AuNCs on laser‐induced graphene (LIGE)	CV, DPV	NSE (neuron‐specific enolase)	0.13 pg/mL	0.01–100 ng/mL	20 min incubation	Spiked human serum, PBS	Simple low‐cost fabrication. Excellent sensitivity, reproducibility (RSD 1.23%), specificity (no interference from EGFR, UA, AA, CEA), and long‐term stability (97% signal after 28 days). ELISA‐comparable accuracy (96.6%–104.9% recovery). Promising for early lung cancer screening	[260]
GCE modified with Au@ Ti_3_C_2_T_*x*_ MXene (substrate) and Au@CuS‐Ab_2_ (signal tag)	CV, DPV, EIS	Carcinoembryonic antigen (CEA)	3.8 fg/mL	0.01–500 pg/mL	—	Human serum	A sandwich‐type immunosensor combining the conductivity of Au@MXene and catalytic activity of Au@CuS nanozymes. Exhibited excellent sensitivity and selectivity with high reproducibility (RSD 1.16%) and good recovery in spiked serum (99.81%–100.1%). Stable for 30 days (retained 85.2% signal). Strong peroxidase‐like signal amplification using H_2_O_2_	[252]
AuNPs/MXene Ti_3_C_2_ modified carbon electrode	SWV, CV, EIS	HPV‐18 DNA	1.95 pM	10 pM–500 nM	60 min	PBS, human urine	Highly stable, amplification‐free CRISPR biosensor with broad dynamic range; robust antifouling and storage stability (up to 2 months); retains > 70% performance	[261]
AuNPs‐APTES‐Nb_2_C‐MXene on SPCE with SH‐ssDNA aptamer	DPV, CV, EIS	Progesterone	14 pM (PBS), 17 pM (sweat)	50–160 pM	40 min	PBS, female sweat, serum	Ultra‐sensitive and selective detection using AuNP‐decorated Nb_2_C‐MXene; first aptasensor for progesterone in sweat; stable, reproducible, low‐cost SPCE platform	[262]
P_2_Mo_17_V/Cs‐Ti_3_C_2_T_*x*_ MXene nanocomposite on GCE	DPV, CV	L‐Tryptophan	0.08 µM	0.1–103 µM	—	Milk (diluted)	Layer‐by‐layer composite enhances conductivity and surface area; best performance with 2 layers. High selectivity, stability (92.2% over 10 days), and reproducibility (RSD ∼2%). Recovery in real milk samples ranged from 95.78%–104.31%	[263]
Ti_3_C_2_T_*x*_ MXene@PDA/MOF on GCE (in situ grown MOF on MXene@PDA)	DPV, CV, EIS	L‐cysteine (L‐Cys)	3.74 nM	0.01–5.0 µM	∼45 min (DPV)	Human serum (spiked)	Composite shows honeycomb‐like structure with high porosity, high electron mobility. MOF enhances signal via electrocatalysis while PDA stabilizes MXene and prevents restacking. Exhibits excellent selectivity, reproducibility (RSD < 3.9%), and recovery (~100%−101.7%). Retained 96.7% of signal after 15 days. Strong anti‐interference and real sample applicability demonstrated	[264]
Ti_3_C_2_ MXene‐derived potassium titanate nanoribbon‐modified glassy carbon electrode (KTNR/GCE)	Oxidation current (Ipa)	Enrofloxacin	LOD (CV): 2.79 nMLOQ: 8.44 nM	0.007–18.066 μM	—	River/marine water, soil, fertilizer, milk, honey, eggs	KTNR shows excellent surface area (108 m^2^/g), stability up to 200 cycles, and selectivity vs. structurally related fluoroquinolones; non‐enzymatic and non‐aptameric system	[265]
Ti_3_C_2_T_*x*_ MXene/AgNPs@pSC4/MB nanoprobe on Au electrode with aptamer	SWV, CV, EIS	Staphylococcal enterotoxin B (SEB)	1.76 × 10^−7 ^ng/mL	0.0001–100 ng/mL	30 min (SEB), 10 min (probe)	Milk	High‐performance nanoprobe leveraging synergy of MXene and AgNPs; para‐sulfonatocalix [4]arene (pSC4) enhances binding with SEB; stable, specific, anti‐interference; recoveries 94.2%–104% in milk; first report of pSC4‐mediated SEB biosensing	[266]
Ti_3_C_2_ MXene + MWCNT with MIP on GCE	DPV, CV	Histamine	0.83 mg/L	1–280 mg/L	4 min rebinding; 6 min extraction	Canned tuna (spiked), PBS	Fast and selective detection. Reproducibility (RSD < 5.3%), high specificity (≤5% signal variation with interferents), wide linear range, and short analysis time. MXene/MWCNT improved conductivity and active surface area. Sensor performance validated against HPLC	[267]
N, S‐CDs/Ti_3_C_2_T_*x*_‐modified GCE	Voltammetric	Dopamine	0.91 µM	1–1000 µM	—	Human urine	N, S‐CDs prevent Ti_3_C_2_T_*x*_ stacking, increase surface area 3×, boost conductivity; recoveries: 96.78%–104.85%	[268]
Ti_3_C_2_T_*x*_ MXene/CNTs substrate + MIL‐101 MOFs‐Ab2	DPV, CV	Caprinae collagen	83 pg/mL	0.1–1000 ng/mL	1 h incubation × 2 (Ig & Ab2)	Archaeological leather artifacts, soil	First use in archaeology. Outperformed ELISA (1373× lower LOD), high stability (85% signal retention after 7 days), ultra‐sensitive and specific to Caprinae collagen. Enabled micro‐trace detection and species differentiation (e.g., sheep vs. wool). Strong correlation with artifact preservation status	[269]
Ti_3_C_2_T_*x*_ MXene@Ni_3_(HITP)_2_@AuNPs + NiCo@Fc‐MWCNTs‐LDH on GCE	DPV, CV, EIS	Extracellular vesicles	13.79 particles/mL	1.6 × 10^2^–1.6 × 10^6 ^particles/mL	—	Human serum (MCF‐7 EVs)	Tetrahedral DNA (TDN) with CD63 aptamer anchored via AuNPs allows specific EV capture; NiCo@Fc‐MWCNTs‐LDH enhances signal via Fc redox reaction. Sensor shows excellent selectivity, reproducibility (RSD 0.45%), and stability (<4% drop in 20 days). Suitable for early clinical cancer diagnosis	[270]
Ti_3_C_2_T_*x*_ MXene@MOF‐modified GCE with antibody probe	EIS, CV	GPNMB	180.33 pg/mL	1–100 ng/mL	∼1.5 h (incubation)	Human serum (PD patients)	MOF‐coated MXene enhanced oxidation resistance, conductivity, and surface area; highly selective and stable GPNMB detection; AUC = 0.91 for PD discrimination	[271]
Ti_3_C_3_T_*x*_‐MXene + NH_2_‐pDNA on SPCE	EIS, CV, DPV	SARS‐CoV‐2 DNA	0.004 pM (buffer);0.003 pM (serum)	0.1 pM–1 µM (buffer);1 pM–1 µM (serum)	12 min incubation	Phosphate buffer; human serum (spiked)	Selective to SARS‐CoV‐2 gene (nsp2); excellent specificity; long shelf life (up to 40 days); low‐cost fabrication (~10 INR/electrode); rapid and label‐free; better than CRISPR and other nucleic acid biosensors in terms of time and ease of use	[272]
Ti_3_C_2_T_*x*_ MXene + AuNPs on screen‐printed electrode	DPV, CV, EIS	HBsAg, anti‐HIV, anti‐TP	0.01 ng/mL (HBsAg); 0.11 ng/mL(HIV); 0.10 ng/mL (TP)	HBsAg: 0.05–1000 ng/mL; HIV: 0.35–140 ng/mL; TP: 0.25–100 ng/mL	∼45 min total assay	Artificial blood, human serum	In situ AuNPs and MXene deposition improves conductivity and surface area (nanoflower structure). Multiplex detection from a single sample. High specificity, stability (28 days), reproducibility (<5% RSD), POC‐suitable. Perfect correlation with clinical diagnostic device results (kappa = 1.000).	[273]
Boron/phosphorus co‐doped mesoporous Ti_3_C_2_T_*x*_ MXene (BPMMX)	DPV, CV, EIS	Guanine	6.4 nM	9.43 nM–117 μM	∼6 min incubation	Human serum (diluted 100×), PBS	High conductivity, large surface area (214 m^2^/g), great reproducibility (RSD < 1.7%), long‐term stability (3.1% signal drop in 7 days), strong anti‐interference (UA, AA, DA, etc.), enhanced activity due to P/B co‐doping and mesoporous network. Suitable for real biosample detection	[274]
Ti_3_C_2_T_*x*_ MXene + Truncated Zika Aptamer on microelectrode	EIS, CV	Zika E protein	6.26 pM (DIW); 3.82 pM (serum)	10 pM–1 μM	10 min total	DIW, 10% human serum	Ultra‐fast and miniaturized POC device using ACEF, high specificity, good selectivity against flavivirus proteins. Blind tests show < 6% error. Excellent stability and low‐cost fabrication. Clear signal enhancement due to MXene/aptamer synergy. Promising for clinical diagnosis and field applications	[275]
Zr‐crosslinked Ti_3_C_2_ MXene on AuNP‐PNA network	Conductance, EIS	*M. tuberculosis* 16S rDNA	20 CFU/mL	10^2^–10^8 ^CFU/mL	2 h total	Simulated sputum (spiked)	High specificity (no interference from 5 strains), RSD ∼3.2%, stable (<6.4% signal fluctuation in 30 days). Direct MXene bridging enhances signal amplification. Superior to ELISA, SPR, PCR in cost and speed. McNemar test showed no significant difference vs. culture (*p* = 0.625)	[276]
CNT‐bridged Ti_3_C_2_T_*x*_ MXene arrays + MB‐aElisa	DPV, EIS	CEA (carcinoembryonic antigen)	1.6 pg/mL	0.005–1.0 pg/mL	1 h incubation	Human serum (CRC patients)	One‐step homogeneous electrochemical (HEC) sensor using CNT‐MXene conductive network; high selectivity, low background, good reproducibility (RSD 4%), stable over 15 days, recovery 93.4%–109.4%; useful for POC of tumor markers; signal generated from in situ enzymatic production of 1‐NP	[277]
Ti_3_C_2_T_*x*_ MXene‐CMCS‐Hemin/AuNPs/SPCE + LDL aptamer	DPV, CV, EIS	LDL	0.095 µM	0.1–4.0 µM	30 min incubation	Human serum samples	Label‐free aptasensor using redox‐active Hemin in MXene‐CMCS composite. High specificity via LDL aptamer; optimized for pH 6.9 and 35°C. Demonstrated excellent stability (73% signal retention at day 11), specificity (low interference), reproducibility (RSD < 8.1%), and accurate quantification in real serum samples	[278]
AuNP‐decorated Ti_3_C_2_ MXene + Au@COFs (COF‐based probe)	DPV	Aβ1‐42 oligomers	4.27 fg/mL	0.01–180 pg/mL	—	Human serum	Aptamer and TB modified COFs as signal labels; double amplification strategy (Au‐MXene and COFs); high sensitivity and selectivity	[279]
Ti_3_C_2_T_*x*_ MXene/Pt/C nanocomposite + DNA/RNA probe	DPV, CV, EIS	SARS‐CoV‐2 RNA	0.4 aM	1 aM–100 nM	30 min	PBS, nasopharyngeal swabs, saliva, serum	Ultra‐sensitive biosensor for COVID‐19 RNA; also works without extraction step; shows high accuracy (100%), specificity (97.87%) and LOQ 60 copies/mL	[280]
NH_2_‐ Ti_3_C_2_ MXene@COFTA‐DH on GCE	CV, DPV, EIS	Guanine (GA), adenine (AE)	0.07 µM/0.44 µM	GA: 0.6–230 µM; AE: 1–450 µM	—	Denatured herring sperm DNA	Sandwich structure prevents aggregation, enhances electrocatalytic activity; excellent selectivity, stability (2.5% and 3.8% drift in 7 days), reproducibility (RSD < 2.7%), anti‐interference verified with multiple ions/organics; GA/AE ratio consistent with theory in real sample	[281]
AuNPs@ Ti_3_C_2_ MXene‐modified SPGE with aptamer	ACV, SWV, chronoamperometry	VEGF (vascular endothelial growth factor)	16 fM	2.5–250 pM	10–40 min (equilibrium)	Artificial urine, PBS buffer	Demonstrated high sensitivity, signal‐to‐noise ratio, and stability under continuous interrogation; improved electrode surface area by ∼30× over bare AuE; successfully implemented real‐time detection with KDM correction; high specificity vs. DNA, RNA, IgG, BSA; aptamer density and SDS pretreatment optimized; compatible with both MB and Fc redox reporters; LOD determined at low aptamer coverage	[282]
PAMAM@ Ti_3_C_2_T_*x*_ MXene on SPCE	DPV, CV, EIS	Folate receptor	5.6 ng/mL	10–1000 ng/mL	40 min	PBS buffer	Uses folic acid‐functionalized PAMAM@MXene as sensing interface; highly selective and stable; reproducible with < 1.7% RSD; selective against IgG, BSA, thrombin; stable over 7 days; good potential for tumor biomarker monitoring applications	[283]
Ti_3_C_2_T_*x*_ nanoribbons on SPCE with Fc‐Au‐Ab_2_ amplification	DPV, EIS	p53 protein	1.0 pg/mL	5.0–200 pg/mL	12 min + 21 min incubation	Human serum	Sandwich‐type immunosensor using carboxylated Ti_3_C_2_T_*x*_ for Ab_1_ immobilization and Fc‐Au for Ab_2_ conjugation. High sensitivity, good selectivity vs. BSA, AFP, IgG, and CEA; stability up to 40 days, reproducibility RSD = 3.68%. Recovery from serum: 94.7%−97.0%. Detects stable mutant p53 isoform relevant to cancers	[284]
Chit/ChOx/Ti_3_C_2_T_*x*_/GCE	CV	Cholesterol	0.11 nM	0.3–4.5 nM	—	—	Very low LOD for cholesterol	[285]
PTA (polyaniline/N‐Ti_3_C_2_T_*x*_ MXene copolymer) on GCE	CV, EIS, DPV	Glutathione (GSH)	5.21 × 10^−14 ^M	1.0 × 10^−12^– 1.0 × 10^−8 ^M	—	Human serum	Sensor shows net‐like structure, excellent reproducibility (RSD < 1.68%), anti‐interference (<10%), and good recovery (94.9%−102.7%) in serum. High stability over 10 days	[149]
MIP/Ti_3_C_2_/NH_2_‐CNTs/GCE	DPV	Fisetin	1.0 nM	0.003 μM–20.0 μM	—	Buffer	Good selectivity due to MIP, NH_2_‐CNTs prevented MXene aggregation	[286]
Ti_3_C_2_/TiO_2_/BiVO_4_	PEC	Soluble CD44	14 fg/mL	0.22 pg/mL–3.2 ng/mL	—	Buffer	Ultra‐sensitive detection with heterojunction and broad spectrum activity	[287]
Au/ Ti_3_C_2_T_*x*_ MXene/PU 3D scaffold	CV, amperometry	H_2_O_2_	3.928 nM	0.1–100 µM	Immediate (live cells)	MDA‐MB‐231 cells in 3D culture	Real‐time monitoring of H_2_O_2_ from cells stimulated with PMA; excellent selectivity (minimal interference from UA, DA, AP, GLU), reproducibility (RSD 3.4%), stability (95% after 16 days), biocompatibility confirmed over 7 days. Strong correlation between signal and cell number	[288]
Pt@SWCNTs‐ Ti_3_C_2_ MXene‐rGO Screen‐printed carbon electrode (SPCE)	Simultaneous and separate	BPA and DM‐BPA	2.5 nM (BPA)2.8 nM (DM‐BPA);	0.006–11.4 µM (BPA)0.006–9.8 µM (DM‐BPA)0.006–7.4 µM (simultaneous)	—	Tap water, plastic, thermal paper	First report of simultaneous detection of BPA and DM‐BPA with < 44 mV potential difference; excellent reproducibility and interference resistance	[289]
AuNPs@Ti_3_C_2_ MXene on printed Ag/PET	CV	5hmC (DNA base)	0.632 pM	0.632 pM–63.2 pM	5 min incubation	Genomic DNA (cells, tissues, tumor models)	Label‐free detection of 5‐hydroxymethylcytosine (5hmC); sensor distinguishes 5hmC from 5mC and cytosine via lower adsorption to AuNPs. Nozzle‐jet printed device shows strong reproducibility, high stability (RSD 4.36% over 50 cycles), and successfully detects 5hmC loss in cancer samples	[290]
PDA@ZnMoO_4_/Ti_3_C_2_T_*x*_ MXene modified GCE	DPV, CV, EIS	*Listeria monocytogenes*	12 CFU/mL	10–10^7 ^CFU/mL	∼1 h	Milk, smoked seafood	High selectivity and stability, real sample testing, synergistic composite with PDA and ZnMoO_4_ enhancing MXene functionality. Repeatable and reproducible performance	[291]

**FIGURE 16:**
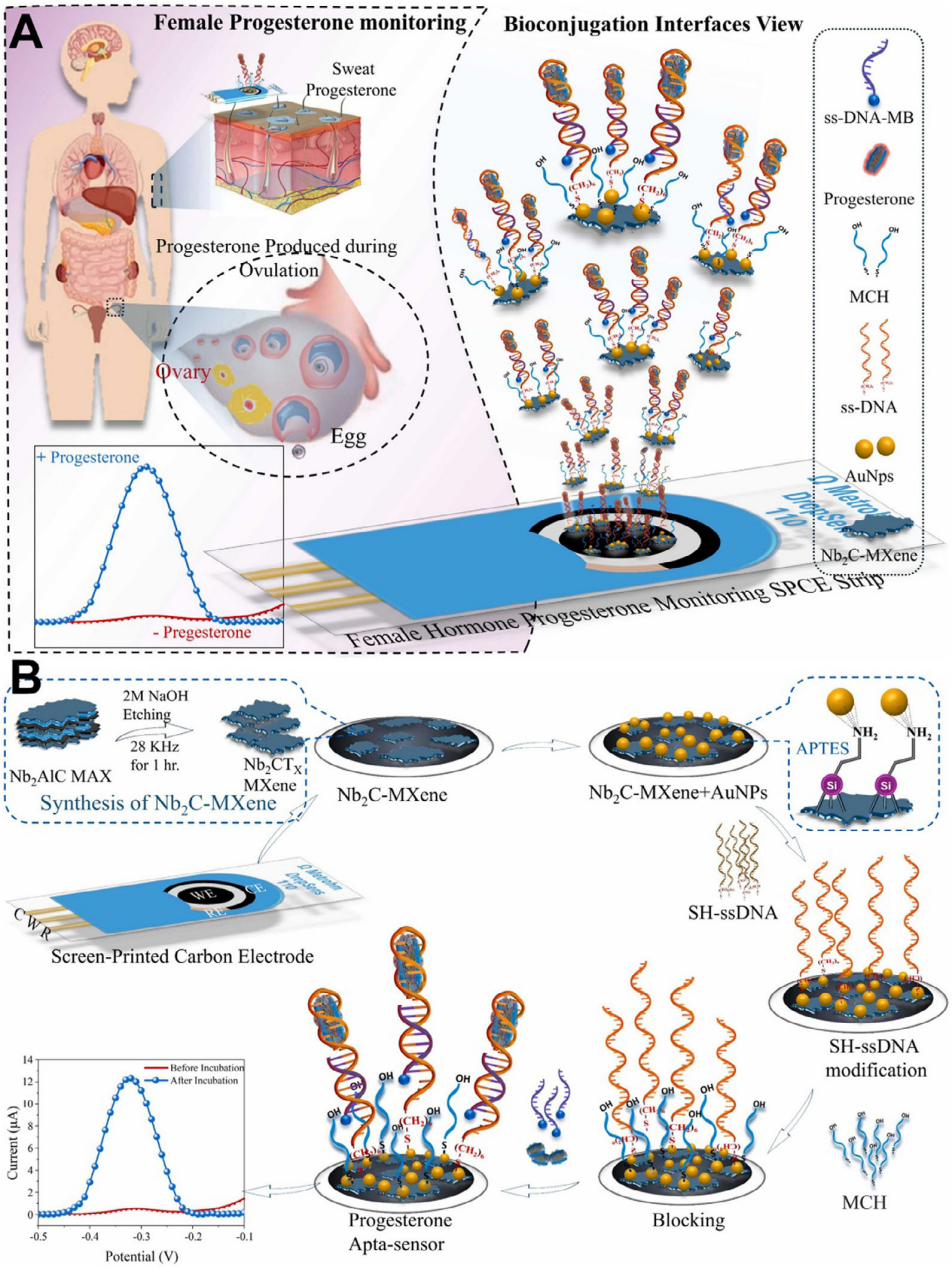
(A) Schematic representation of the electrochemical aptamer sensor mechanism for progesterone detection. The sensor utilizes a target‐induced strand displacement strategy, where progesterone binding to the aptamer triggers release of a complementary redox probe (ssDNA‐MB), generating a measurable electrochemical signal. (B) Stepwise modification of the screen‐printed carbon electrode (SPCE) surface: Nb_2_C MXene nanosheets are functionalized with APTES and decorated with AuNPs, enabling immobilization of the progesterone‐specific ssDNA aptamer [262]. Copyright 2025. Reproduced with permission from Elsevier.

Beyond conventional immunoassays, MXene‐based platforms have also been integrated with aptamer‐based recognition systems, which offer improved stability and easier chemical modification compared to antibodies. Aptamer‐functionalized MXene electrodes have been developed for the detection of thrombin and other clinically relevant proteins, achieving low detection limits and high selectivity driven by the strong affinity and structural specificity of aptamer–target interactions (Figure 16) [262]. In these systems, MXenes provide a conductive interface that transduces target‐induced conformational changes in the aptamer into measurable electrochemical signals. This signal transduction mechanism enables highly sensitive detection while maintaining structural simplicity compared to traditional immunoassays. The inherent flexibility of aptamer engineering further supports multiplexed and programmable sensing strategies, underscoring the adaptability of MXene‐based platforms for complex diagnostic applications. As summarized in Table 8, MXene‐based systems accommodate diverse recognition elements—including antibodies, aptamers, and nucleic acids—enabling a broad spectrum of sensitive and selective biomarker detection strategies.

Nucleic acid sensing represents another important application area, particularly in the context of genetic diagnostics and pathogen detection. MXene‐based DNA sensors typically rely on hybridization‐induced changes in electrochemical signals, where single‐stranded DNA probes are immobilized onto the MXene surface. The high conductivity and surface functionality of MXenes facilitate efficient probe immobilization and rapid signal transduction. For instance, Ti_3_C_2_T_*x*_‐based platforms have been employed for the detection of disease‐associated DNA sequences, including HPV‐18 and SARS‐CoV‐2, achieving high sensitivity and rapid response times [261, 272]. Signal amplification strategies, such as enzyme labels or nanomaterial tags, are frequently integrated to further enhance analytical performance. More advanced biosensing architectures incorporate MXenes into sandwich‐type assays and multistep amplification cascades. For example, MXene‐based composites combined with metal–organic frameworks and enzyme labels enabled ultrasensitive detection of cancer biomarkers, with detection limits reaching the femtomolar range [252, 277]. These systems exploit the hierarchical organization of hybrid materials to maximize active surface area, enhance catalytic activity, and improve electron‐transfer efficiency. In addition, the integration of MXenes into microfluidic and paper‐based formats has enabled portable and point‐of‐care diagnostic devices, highlighting their suitability for practical biomedical applications.

An emerging direction in this field is the use of MXene‐based platforms for real‐time monitoring of biomolecular processes in living systems. For example, MXene‐integrated sensors have been developed for the detection of reactive oxygen species and biomarker release from cells, enabling dynamic tracking of cellular activity [288]. These applications underscore the biocompatibility, responsiveness, and environmental adaptability of MXenes in biologically relevant settings. Despite these advances, several challenges remain. The stability of biological recognition elements such as enzymes and antibodies can deteriorate over time, affecting long‐term sensor performance. Nonspecific adsorption and biofouling in complex biological matrices further reduce selectivity and contribute to signal drift. In addition, the intrinsic susceptibility of MXenes to oxidation under physiological conditions can compromise device stability. Addressing these limitations will require antifouling surface engineering, improved immobilization strategies, and protective architectures that preserve MXene conductivity while maintaining high analytical sensitivity.

### 5.4 Neurotransmitters and Pharmaceuticals

**FIGURE 17:**
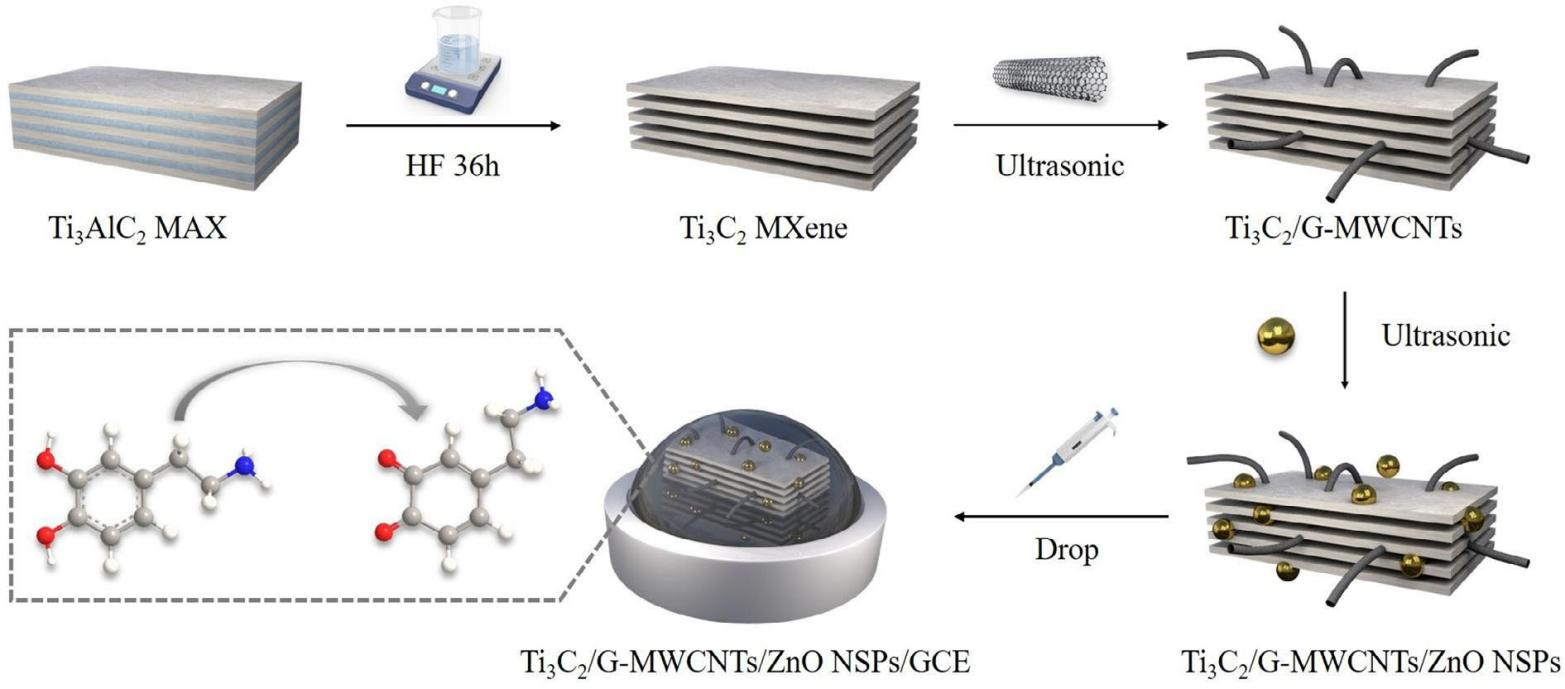
Schematic illustration of the fabrication process of the Ti_3_C_2_/G‐MWCNTs/ZnO‐modified glassy carbon electrode (GCE) and its application for dopamine (DA) detection. The multilayer Ti_3_C_2_ MXene is synthesized via HF etching of Ti_3_AlC_2_ MAX phase, followed by functionalization with acid‐treated graphitized multi‐walled carbon nanotubes (G‐MWCNTs) and ZnO nanospheres [293]. Copyright 2022. Reproduced with permission from Elsevier.

The electrochemical detection of neurotransmitters and pharmaceutical compounds represents a particularly demanding application domain, as these analytes are often present in complex biological environments and exhibit overlapping redox potentials with common interferents. Molecules such as dopamine (DA), uric acid (UA), and ascorbic acid (AA), as well as various drug compounds, frequently coexist, creating significant challenges in selectivity and signal resolution. Within this context, MXene‐based sensors have demonstrated strong potential due to their ability to combine rapid electron‐transfer kinetics with tunable surface chemistry, enabling both enhanced sensitivity and improved discrimination between structurally similar analytes. Detection of DA has emerged as a model system for evaluating MXene‐based electrochemical sensors because of its physiological relevance and strong interference from AA and UA. Pristine Ti_3_C_2_T_*x*_ MXene electrodes already exhibit improved electron‐transfer kinetics compared to conventional carbon‐based materials, resulting in higher peak currents and reduced peak‐to‐peak separation. However, the most significant advances have been achieved through hybrid material design. For instance, Ti_3_C_2_T_*x*_‐modified glassy carbon electrodes have been shown to effectively resolve the overlapping oxidation signals of AA, DA, and UA, enabling simultaneous detection with high sensitivity and reproducibility [292]. The system exhibited wide linear ranges and low detection limits (AA, 4.64 μM; DA, 0.06 μM; UA, 0.075 μM), with voltammetric analysis confirming well‐resolved peaks and adsorption‐controlled redox behavior [292]. This performance arises from the synergistic role of MXene surface chemistry (–OH, –O, –F terminations), high conductivity, and layered architecture, which together promote selective adsorption and differential electron‐transfer kinetics. Further performance improvements have been achieved through hybrid architectures. A hierarchical electrode composed of Ti_3_C_2_T_*x*_ MXene, graphitized multiwalled carbon nanotubes (g‐MWCNTs), and ZnO nanospheres demonstrated exceptional sensitivity for dopamine detection, where MXene provided conductive pathways, CNTs facilitated electron percolation, and ZnO contributed abundant adsorption sites (Figure 17) [293]. In parallel, integration of MXenes with graphene quantum dots (GQDs) has enabled enhanced peak resolution and reduced interference through quantum confinement effects and oxygen‐rich edge functionality, improving dopamine detection in real samples [294]. Collectively, these hybridization strategies highlight the versatility of MXenes: inorganic nanostructures primarily enhance conductivity and structural stability, while carbon‐based quantum materials improve selectivity and signal discrimination, enabling tailored sensor performance for complex analytical environments. The comparative performance of MXene‐based neurotransmitter sensors is summarized in Table 9, where hybrid systems consistently outperform pristine MXene electrodes.

**TABLE 9:** MXene‐based electrochemical sensors for neurotransmitter detection.

Sensor configuration	Measurement type	Target molecule	LOD	Linear range	Response time	Matrix	Observations/remarks	Ref.
Ti_3_C_2_T_*x*_ MXene integrated with Zr/Ni ferrocene‐based MOF (ZrNi‐Fc/MXene composite) on GCE	Electrochemical (DPV, CV, EIS; ratio‐type sensor using Fc as internal reference)	Dopamine (DA)	0.07 μM	0.1–70 μM	Incubation ~1.5 h; electrochemical response immediate	Human serum samples	Ratio‐type design stabilizes signal; Ni doping enhances conductivity and catalytic activity; ferrocene ligand provides internal reference; excellent selectivity against UA, AA, 5‐HT, Glu, urea; recovery rates 98.7%–100.5% in serum; reproducibility RSD 1.8%–4.8%	[295]
TiCT_*x*_ (MXene)/GCE	CV, DPV	Ascorbic acid (AA), dopamine (DA), uric acid (UA)	4.64 µM (AA), 0.06 µM (DA), 0.075 µM (UA)	AA, 100–1000 µM, DA, 0.5–50 µM, UA, 0.5–4 & 100–1500 µM	–	Human urine	Simultaneous detection with distinct oxidation peaks for AA, DA, UA. Good stability (89.6% after 25 days), reproducibility, and selectivity. Recovery in real samples: 100.5%–103%. MXene enhances peak resolution via π–π and electrostatic interactions	[292]
Ti_3_C_2_:GQDs (1:3) nanocomposite on GCE	CV, DPV, EIS	Dopamine	1.8 µM	40–400 µM	–	Human urine, sweat	MXene improves conductivity and GQDs increase surface area. Sensor shows high selectivity (minimal interference from AA, UA, glucose, salts), excellent repeatability (RSD = 0.7%), and high recovery (urine, 98.9%; sweat, 94.1%)	[294]
ABSACG (CeO_2_/Ti_3_C_2_T_*x*_ MXene/amyloid BSA hydrogel) on SPE	DPV, EIS, i‐t	Dopamine (DA)	0.017 µM	0.05–300 µM	–	Artificial + real sweat	Wearable sensor with excellent antifouling (92.6% signal retention after 48 h), antimicrobial (99.5% and 96% against *S. aureus* and *E. coli*), mechanical stability, and real sweat detection validated against ELISA; robust long‐term use	[296]
Ti_3_C_2_ MXene quantum dots on 3D‐printed graphene/PLA	CV, EIS	Dopamine	3 nM	0.01–20 μM	60 s incubation	PBS (pH 7.2), DA injection	Excellent anti‐interference with AA/UA, high stability (RSD < 2.3% in 7 days), dual sensitivity regions (0.01–1.4 μM and 1.4–20 μM), enhanced conductivity and active area due to MQDs. High recoveries (94.24%–102.26%) from pharmaceutical injection samples	[297]
Ti_3_C_2_ MXene + G‐MWCNT + ZnO on GCE	CV, DPV, EIS	Dopamine	3.2 nM	0.01–30 μM	120 s (enrichment)	Human serum (spiked, pretreated)	High stability (7.4% current drop in 25 days), excellent anti‐interference against amino acids and metabolites, reproducibility (RSD < 1.2%). Synergistic enhancement by ZnO and G‐MWCNTs on Ti_3_C_2_. HOMO/LUMO analysis confirms hydroxyl site involvement in DA redox	[293]
FeVO_4_ nanoflakes with Ti_3_C_2_ MXene nanocomposite on SPCE	Electrochemical oxidation	Serotonin (5‐HT)	5.88 nM	25–750 nM	–	Human serum	Synergistic effect between FeVO_4_ and Ti_3_C_2_ improves conductivity and active surface area; excellent selectivity and stability	[298]
Ti_3_C_2_/MXene/GCPE	CV	Adrenaline	9.5 nM	0.02–10 μM, 10–100 μM	–	Pharmaceutical	First use of Ti_2_C MXene in EC sensors; recoveries ~ 99%–101%	[299]

**FIGURE 18:**
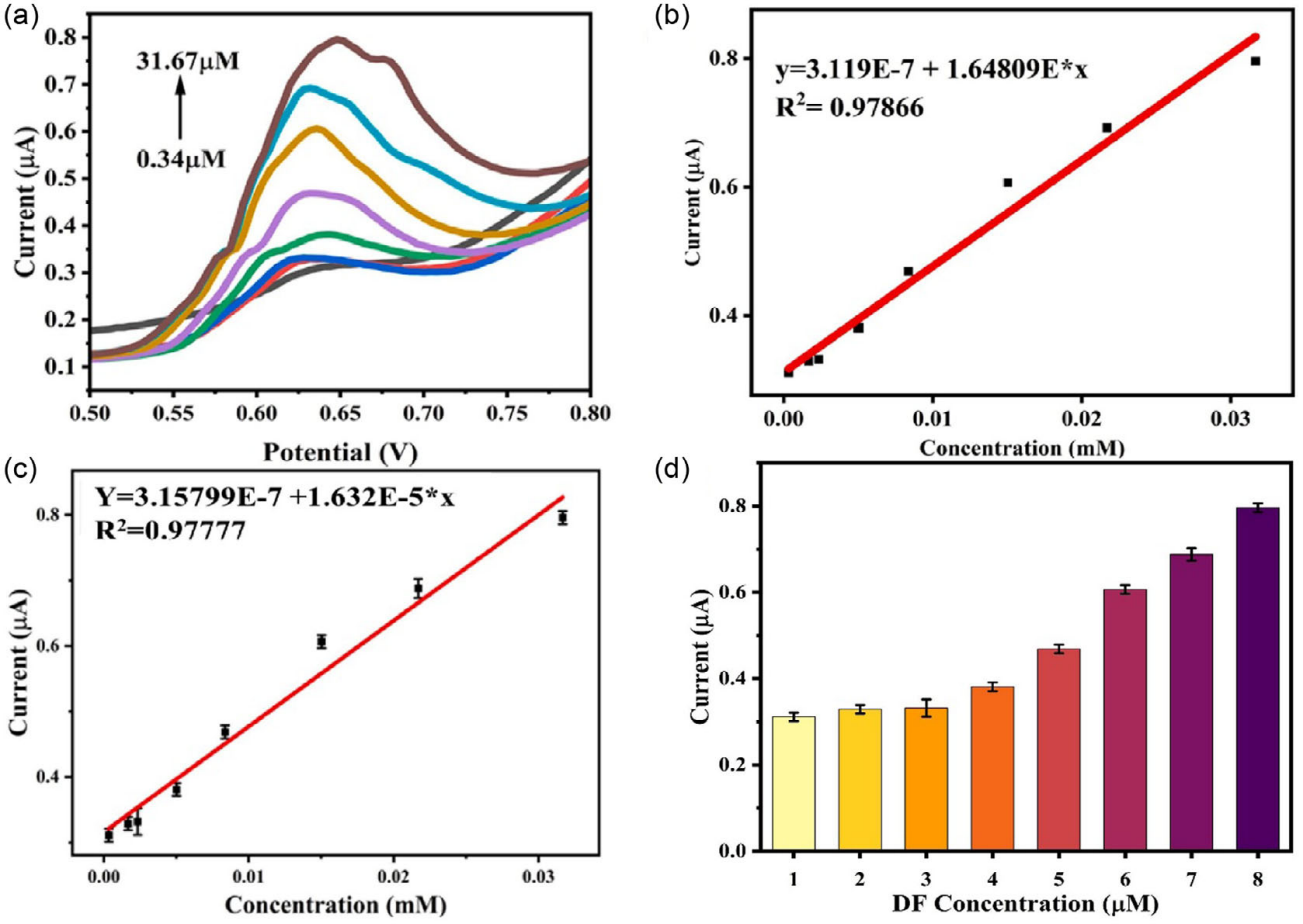
Analytical performance and reproducibility of MXene/CuO/GCE sensor for diclofenac detection. (a) DPV responses at increasing DF concentrations (0.34–31.67 μM), showing progressive oxidation peak enhancement. (b) Calibration plot with *R* ^2^ = 0.9786, confirming linearity and sensitivity. (c) Triplicate DPV measurements using three independently fabricated electrodes, demonstrating high reproducibility. (d) Long‐term stability test over 90 days, showing minimal signal degradation and confirming electrode robustness [300]. Copyright 2024. Reproduced with permission from Elsevier.

**FIGURE 19:**
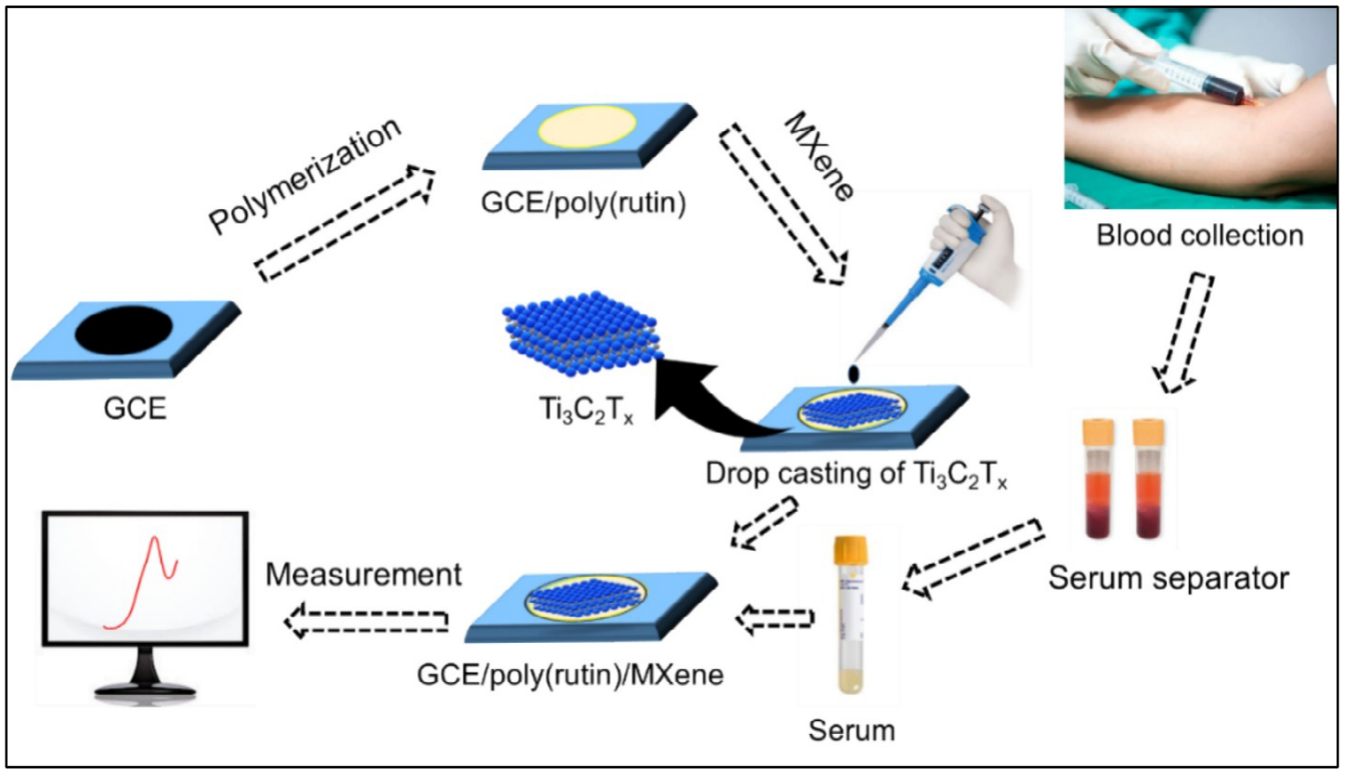
Systematic illustration of the fabrication process for the GCE/poly(rutin)/Ti_3_C_2_T_*x*_ electrochemical sensor used in CIP detection. The GCE is electropolymerized with rutin hydrate to form a conductive poly(rutin) film. Subsequently, Ti_3_C_2_T_*x*_ MXene nanosheets are drop‐cast onto the poly(rutin)‐modified surface, forming a hybrid interface with enhanced electron transfer and adsorption capacity [302]. Copyright 2024. Reproduced with permission from Elsevier.

The detection of pharmaceutical compounds extends electrochemical sensing strategies to structurally diverse molecules, including antibiotics, anti‐inflammatory drugs, and other therapeutic agents. Although many of these compounds are inherently electroactive, their detection is frequently hindered by electrode fouling and matrix interferences. MXene‐based composites have emerged as effective solutions to these challenges due to their enhanced surface stability and antifouling properties. For instance, CuO nanoparticle‐passivated Ti_3_C_2_ MXene electrodes have enabled highly sensitive electrochemical detection of diclofenac, achieving ultralow detection limits (~8.5 nM) [300]. This improved performance arises from the synergistic combination of MXene's high electrical conductivity and CuO's abundant active sites, which together promote adsorption‐controlled electro‐oxidation while minimizing fouling effects. As illustrated in Figure 18, the calibration curves and reproducibility data confirm the robustness and stability of the MXene/CuO sensor platform [300]. In another example, a CuZnS microflower‐anchored MXene nanocomposite was developed for the detection of chloramphenicol (CAP) in biological and dairy samples [301]. Here, MXene serves as a highly conductive backbone facilitating rapid electron transfer, while CuZnS provides catalytically active sites for CAP reduction. This synergistic interaction enables picomolar sensitivity, with limits of detection in the range of 0.15–0.18 nM across broad linear ranges [301]. Additionally, a Ti_3_C_2_T_*x*_ MXene/poly(rutin) composite‐modified glassy carbon electrode has been reported for the electrochemical detection of ciprofloxacin (Figure 19) [302]. The sensor leverages the electron‐rich surface and high conductivity of MXene to promote ciprofloxacin oxidation via electrostatic interactions with its piperazine and carboxyl functional groups. It achieves a wide linear detection range (10^−9^–10^−4 ^M) and a low detection limit of 10^−9 ^M. Notably, this system demonstrates high selectivity and reproducibility in blood serum and represents the first reported MXene/rutin composite for ciprofloxacin sensing via electrochemical oxidation [302]. As summarized in Table 10, MXene‐based sensing platforms exhibit broad applicability across a wide range of pharmaceutical compounds. Their performance is strongly governed by composite design, surface functionalization, and the synergistic interactions between MXene and catalytic components.

**TABLE 10:** MXene‐based electrochemical sensors for pharmaceutical and healthcare applications.

Sensor configuration	Measurement type	Target molecule	LOD	Linear range	Response time	Matrix	Observations/remarks	Ref.
Ti_3_C_2_T_*x*_ MXene/VO_2_@MoO_2_ composite GCEelectrode with anion exchange solid‐phase extraction	Electrochemical (CV, DPV, EIS)	5‐Fluorouracil	2.2 nM	0.01 to 35 μM	Urine, water samples	Pharmaceutical samples	Combination of SPE and MXene‐based composite enhances sensitivity; binary metal oxide (VO_2_@MoO_2_) provides synergistic electrocatalytic effect; suitable for trace‐level detection; recovery 90%–92%	[303]
Ti_3_C_2_T_*x*_ MXene nanosheets (etched and delaminated) on SPE	Electrochemical (DPV, CV, amperometry)	Methamphetamine (METH)	0.18 μM	0.5–100 μM	∼2 s (fast amperometric response)	Human serum, urine, saliva	High sensitivity and selectivity; strong anti‐interference against common drugs and metabolites; reproducibility (RSD < 3%); stability (≈90% signal retention after 20 days); recovery rates 95%–103% in biofluids	[304]
Ag‐ Ti_3_C_2_T_*x*_ MXene (Ti_3_C_2_T_*x*_ decorated with Ag NPs) modified with Apt1 (thiolated), cDNA‐MB, Apt2‐SiO_2_ nanospheres on GCE	Electrochemical (DPV, CV, EIS)	Kanamycin (KANA)	0.12 nM	1–20 000 nM	Not explicitly stated	Cow milk, honey, chicken meat (spiked samples)	Split aptamer design yields signal‐down response; strong anti‐interference capability; excellent selectivity against GEN, CHL, ERY, STR; reproducibility (RSD < 4.2%); stability (95% signal retention after 20 days); recoveries 94%–102%	[305]
Interdigital electrode (IDE) coated with N‐ Ti_3_C_2_T_*x*_/WS_2_ composite; wireless resistance signal monitoring device	Resistive gas sensor (chemiresistive)	Ammonia (NH_3_)	0.28 ppm	3–80 ppm (linear fit, *R* ^2^ = 0.975); also linearity confirmed at 0.5–3 ppm	4.4 s (response), 3.8 s (recovery) at 20°C; in real refrigerated fish tests: 17–51 s response, 8–41 s recovery depending on humidity	Fish fillets stored in refrigerator and cold storage (real‐world spoilage monitoring)	High response (56.57% at 100 ppm NH_3_); excellent selectivity against other gases; stable baseline (low drift); repeatability and long‐term stability (maintains > 52% performance after 13 days); humidity tolerance tested (43%–85% RH); wireless Bluetooth transmission for real‐time monitoring	[306]
Interdigital electrode (IDE) coated with Nb_2_CT_*x*_/SnS_2_ composite	Resistive gas sensor (chemiresistive)	Ammonia (NH_3_)	0.36 ppm	1–100 ppm	∼6 s (response), ~5 s (recovery) at room temperature	Ambient air; simulated food storage environments	Strong selectivity against ethanol, acetone, methanol, and other gases; stable baseline; reproducibility (RSD < 3%); long‐term stability (>90% signal retention after 20 days); enhanced sensitivity due to Nb_2_CT_*x*_/SnS_2_ heterojunction	[307]
Ti_3_C_2_T_*x*_ MXene decorated with CoFeMn‐layered double hydroxide (CoFeMn‐LDH/MXene composite) on GCE	Electrochemical (CV, DPV, EIS)	Ciprofloxacin (CIP)	0.021 μM	0.05–100 μM	∼3 s (fast amperometric response)	Real samples: milk, apple juice, tap water	High sensitivity and selectivity; strong anti‐interference against other antibiotics and metabolites; reproducibility (RSD < 3%); stability (≈90% signal retention after 20 days); recoveries 95%–104% in food and water samples	[308]
Ti_3_C_2_T_*x*_ MXene decorated with Au NPs with aptamer integrating CRISPR/Cas12 amplification	Electrochemical (DPV, CV, EIS)	Aflatoxin B1 (AFB1)	0.00037 ng/mL	0.001–100 ng/mL	∼10 min (including CRISPR reaction; electrochemical readout rapid)	Real food samples: corn, peanuts, milk	Combines MXene conductivity with CRISPR/Cas12 signal amplification; ultrasensitive detection surpassing ELISA; portable format suitable for on‐site testing; strong selectivity against other mycotoxins; recoveries 92%–107% in food matrices	[309]
Ti_3_C_2_T_*x*_ MXene decorated with defect‐engineered NiFe layered double hydroxide (NiFe‐LDH/MXene composite) on GCE	Electrochemical (CV, DPV, EIS)	Nilutamide	0.002 μM	0.01–100 μM	∼3 s (fast amperometric response)	Human serum, pharmaceutical formulations	Defect engineering enhances active sites and electron transfer; high sensitivity and selectivity; reproducibility (RSD < 3%); stability (≈90% signal retention after 20 days); recoveries 95%–104% in serum and drug samples	[310]
Ti_3_C_2_T_*x*_ MXene decorated with Ag NPs and CdTe quantum dots (Ag/MXene‐CdTe composite)	On GCEElectrochemical (DPV, CV, EIS)	Propyl gallate, Vanillin	Propyl gallate: 0.004 μM Vanillin: 0.006 μM	0.01–100 μM (both analytes)	∼3 s (fast amperometric response)	Real food samples: beverages, bakery products	Dual analyte detection with high sensitivity; strong anti‐interference against other phenolic compounds; reproducibility (RSD < 3%); stability (≈90% signal retention after 20 days); recoveries 95%‐105% in food matrices	[311]
Ti_3_C_2_T_*x*_ MXene functionalized with o‐aminophenol (o‐AP@Ti_3_C_2_T_*x*_) integrated with laser‐induced graphene (LIG)	Electrochemical (CV, DPV, EIS)	Fenbendazole	5 nM	10 nM–4 μM (static accumulation); 10 nM–2 μM (stirring)		Real milk samples	o‐AP functionalization enhances hydrophilicity, adsorption, and electron transfer; π‐π stacking and H‐bonding enable specific recognition; excellent stability (>90% signal retention after 7 days); reproducibility RSD < 10%; recoveries 94.8%–99.2% in milk; results consistent with HPLC	[312]
Antibody‐functionalized Ti_3_C_2_T_*x*_ MXene on LIG electrode	DPV, CV, EIS	Vitamin D (25(OH)D_3_)	1 pg/mL	0.1–500 ng/mL	Fast	PBS buffer, human serum	Ultra‐sensitive, enzyme‐free, and portable biosensor; achieved wide clinical range detection; highly selective against interfering molecules; good reproducibility. Future development will address antifouling in complex biological samples	[313]
MXene (Ti_3_C_2_T_*x*_) + CuO on GCE	CV, DPV, SWV, EIS	Diclofenac sodium	0.0083 µM (SWV), 0.0085 µM (DPV)	0.34–31.67 µM	—	Phosphate buffer; pond water	High selectivity and reproducibility; successful real sample validation in pond water (93.6%–111.3% recovery); long‐term stability (90 days); improved sensitivity through CuO synergy; low‐cost, POC applicability for environmental monitoring	[300]
GCE/poly(rutin)/Ti_3_C_2_T_*x*_ MXene	DPV, CV, EIS	CIP	1.0 × 10^−9 ^M	10^−9^–10^−4 ^M	—	Blood serum	First MXene/rutin composite sensor for CIP; high sensitivity (0.49 μA/μM·cm^2^), high selectivity against 100‐fold interferents, stability over 4 weeks, RSD < 0.7%; recoveries 98.96%–99.83%	[302]
Cu‐TAC‐3 (Cu‐intercalated Ti_3_Al_1‐x_C_2_‐OH_*x*_) on GCE	CV, DPV, EIS	5‐Fluorouracil (FLU)	0.54 nM	0.001–83.76 µM	—	Pond, river, tap water, soil sediment	3D network MXene with Cu intercalation showed high surface area, excellent sensitivity (0.7033 µA µM^−1 ^cm^−2^), high selectivity against interferents, and recovery rates > 97% in environmental samples; prepared using eco‐friendly magnetic stirring	[314]
PCN‐222@ Ti_3_C_2_T_*x*_ MXene + aptamer on GC	DPV	Oxytetracycline	0.44 fg/mL	0.01–100 pg/mL (log‐linear)	6 min (detection); 9 min (aptamer loading)	Diluted milk (×50), PBS	Excellent selectivity vs. PCL, KAN, DOX, STR; good reproducibility (ΔI variation negligible); stable over 7 days at –20°C; synergy between MXene conductivity and Zr‐MOF aptamer binding; suitable for real sample testing; recovery: 98%–110% with low RSD values	[315]
ZnMoO_4_/Ti_3_C_2_T_*x*_ MXene nanocomposite on GCE	CV, DPV, EIS	Roxarsone (ROX)	0.0081 µM	0.025–605.65 µM (2 linear ranges)	∼10 min total	Tap water, drinking water, river, pond	High sensitivity (10.413 µA µM^−1 ^cm^−2^), reproducibility (RSD 1.78%), stability (85.25% over 11 days), selectivity against 9 interferents, dual linear range from DPV, adsorption‐controlled redox kinetics, neutral pH optimization, promising for field water analysis	[316]
CuZnS microflowers on Ti_3_C_2_T_*x*_ MXene/SPCE	DPV, i‐t amperometry, CV, EIS	Chloramphenicol (CAP)	0.18 nM (DPV); 0.15 nM (i‐t)	0.002–6.75 µM (DPV); 0.002–100 µM (i‐t)	5 s (i‐t); not stated for DPV	Honey, milk, urine, eyedrops	Excellent selectivity, wide linear range, high sensitivity (up to 90.25 µAµM^−1 ^cm^−2^), good reproducibility and stability. Recovery 96.5%–98.8%, RSD < 2.81%. Superior to many reported CAP sensors. Suitable for real sample applications in food safety and clinical diagnostics	[301]
Nb_2_O_5_/Nb_2_CT_*x*_/MWCNTs‐COOH‐modified GCE	CV, DPV, EIS	Clozapine (CLZ)	8.38 nM	0.01–1.0 µM and 1.0–10.0 µM	90s enrichment time	Human serum, urine (100× diluted)	High sensitivity due to synergy of Nb_2_O_5_ and MXene; excellent anti‐interference; recovery 92.5%–106.5%; repeatability and reproducibility < 2.2% RSD	[317]
rGO‐PSS@ Ti_3_C_2_T_*x*_ MXene heterostructure on GCE	CV, DPV, EIS, chronocoulometry	Rutin	1.8 nM	0.005–10 µM	—	Rutin tablets, Tartary buckwheat tea	Synergistic interaction between MXene and PSS‐functionalized rGO improved electron transfer and surface area. High selectivity and stability; anti‐interference verified with flavonoids (quercetin, baicalin). Validated with HPLC; suitable for drug and food quality control	[318]
Ag_2_Te/ Ti_3_C_2_T_*x*_ MXene composite on GCE	CV, DPV, LSV, EIS	Roxarsone	0.32 nM	0.03–2310 μM	—	Chicken, pork (real meat samples)	High electrocatalytic activity from MXene‐Ag_2_Te synergy; wide detection range; high reproducibility and selectivity even in complex matrices like meat	[319]
Nb_2_CT_*x*_ MXene + NRGO on GCE	SWV, CV, EIS	Fluoxetine (FLX)	0.34 µM	1.0–100 µM (two linear regions: 1–10 µM and 10–100 µM)	—	Human serum and urine (diluted 100× with PBS)	High selectivity, reproducibility, stability, and performance in biological matrices; better than many prior FLX sensors	[320]
Ti_3_C_2_T_*x*_ MXene‐Au NPs and MB/AuPd NPs/UiO‐66 with catalytic hairpin assembly (CHA)	SWV, EIS, CV	Tetracycline	0.789 pg/mL	0.001–500 ng/mL	—	Piped water, milk, honey, turtle feed	Dual signal amplification using MXene‐Au NPs and CHA strategy; aptamer binds three ssDNA strands to enhance amplification; high sensitivity and specificity; good reproducibility (RSD 2.7%) and stability (>90% signal retention over 15 days);demonstrated application in real food matrices with recoveries 92.0%–112.2%	[321]
SiO_2_‐ Ti_3_C_2_ MXene‐SnO_2_ on Ni foam	CV, DPV, EIS, LSV	CIP	0.5 µM	0.5–0.45 µM	—	PBS (pH 7.0), tablets	Ternary nanocomposite showed enhanced conductivity and large surface area; fabricated via self‐assembly; high selectivity vs. common interferents; stable over 20 days with < 5% signal loss; reproducible and suitable for pharmaceutical analysis	[322]
Ti_3_C_2_T_*x*_ MXene/MWCNTs composite on GCE	CV, LSV, EIS	Synephrine	0.167 µM	0.5–70 µM	120 s accumulation	*Fructus aurantii* extract	High conductivity and electrocatalytic activity from MXene/MWCNTs; excellent selectivity vs. interferents (UA, DA, Glu, VC, ions); stable (96.4% signal after 1 month); comparable to HPLC; good real sample recovery from herbal extracts	[323]
Fe_3_O_4_/Ti_3_C_2_ MXene on GCE	CV, DPV, EIS	Paracetamol	0.63 nM	0.0–110 µM	—	Distilled water, sea water, drug tablets	Excellent selectivity and ultra‐low LOD; reproducible over 7 days (RSD 3%–4%); applied to pharmaceutical formulations and seawater samples with > 98% precision; sensor shows better performance than comparable methods	[324]
N, S‐doped 3D V_2_C MXene nanoflower/holey MWCNT	CV, EIS, amperometry	Nicotine	0.058 µM	10–500 µM	—	PBS, artificial urine, tobacco extract	Inkjet‐printed flexible sensor with excellent conductivity and high stability; real sample analysis with artificial urine and tobacco extract confirmed performance	[325]
Ti_3_C_2_T_*x*_ MXene/SPE	CV	Acetaminophen, isoniazid	ACOP 0.048 μM, INZ 0.064 mM	ACOP 0.25–2000 μM, INZ 0.1–4.6 mM	—	Buffer	Simultaneous detection with separated peak potentials	[326]
Ru/NiFe‐LDH‐ Ti_3_C_2_T_*x*_ MXene‐modified SPCE	LSV, CV, EIS	Chloramphenicol	2.2 nM	0.01–275 µM	—	Milk, honey	Ru NPs enhance conductivity and surface area; shows excellent sensitivity (152.44 μA/μM·cm^2^), reproducibility (RSD 0.69%), and recovery (97.01%–106.97%). Stable for 25 days (87.45% signal retained)	[327]
Nb_2_C MXene/MnFe_2_O_4_ (N‐MFO4) on GCE	CV, DPV, EIS	Acetaminophen (AP), dopamine (DA)	AP: 0.079 µM, DA: 0.070 µM	AP: 0.1–1000 µM, DA: 0.1–60 µM	—	AP: Tablet solution, DA: Urine	N‐MFO4 forms Schottky junction via self‐assembly; enhanced charge transfer, low resistance, wide linear range; reproducibility RSD < 2.3%, good selectivity and stability	[328]
HOF/ Ti_3_C_2_T_*x*_ MXene + aptamer on GCE	DPV, CV, EIS	Ampicillin	0.21 fg/mL	0.01–100 pg/mL (log‐linear)	3 min aptamer, ~6 min detection	Tap water, milk (spiked)	Excellent selectivity vs. other antibiotics; high reproducibility (RSD 1.8%–4.5%); good stability over 8 days at 4°C. Superior to prior AMP sensors. BET surface area of 711 m^2^/g; aptamer binds via π‐π and Ti‐O interactions; HOF improves aptamer loading, MXene improves conductivity	[329]
MIP/Fe‐MOF‐NH_2_/CNTs‐NH_2_/ Ti_3_C_2_T_*x*_ MXene on GCE	DPV, CV, EIS	Ofloxacin	13.2 nM	0.1–100 µM	12 min incubation;3 min elution	Milk (four brands, spiked)	Dual‐signal (DA as internal reference), high specificity (IF > 15 for OFL vs. others), excellent reproducibility (RSD = 2.5%), and long‐term stability (93.6% signal after 3 weeks). Real sample recoveries: 96.6%–104.8%. Verified via HPLC. MIP‐RECS offers improved robustness and accuracy	[330]
Ti_3_C_2_T_*x*_ MXene + CuFe_2_O_4_ + poly‐L‐cysteine on MCPE	CV, SWV	Quercetin	1.6 nM	0.005–0.7 μM, 0.7–10 μM	20 min incubation	Quercetin capsules (ethanol extracted, citrate buffer pH 5)	First report using MXene/CuFe_2_O_4_/MIP for Que; synergy enhances conductivity, stability, and active surface area. Strong selectivity and anti‐interference. Real sample recoveries with RSD < 3.1%. Excellent repeatability, reproducibility, and 1‐month stability (95.4%). Response controlled by adsorption kinetics (2e^−^/2H^+^ redox)	[331]
MnO_2_‐Pi/ Ti_3_C_2_T_*x*_ MXene on carbon fiber paper (CFP)	DPV, CV, EIS	Vitamin B6	0.021 µM	0.06–650 µM	—	Pharmaceutical tablets (spiked)	Brush‐coated MXene improves conductivity and adhesion of MnO_2_‐Pi; coral‐like MnO_2_‐Pi structure enhances active surface area; excellent reproducibility (RSD ∼1%), recovery ∼100%, high stability (1.9% decrease over 30 days), strong anti‐interference from common vitamins and salts	[332]
Ti_3_C_2_T_*x*_ MXene fibers + molecularly imprinted polymer (MIP)	DPV, CV, EIS	Hydrocortisone	0.17 nM	0.5 nM–10 µM	15 min	Human serum (spiked)	Wet‐spun MXene fibers provided excellent conductivity and stability, while MIPs added target specificity. The sensor demonstrated high selectivity against analogs, strong anti‐interference, reproducibility (RSD < 5%), and long‐term stability (97.5% signal after 1 month). First report combining wet spinning with MIP sensing for hydrocortisone detection	[333]
MOF‐71/vanadium carbide V_2_CT_*x*_ MXene hydrogel	DPV, CV, EIS	Levothyroxine (LT4) Carbamazepine (CBZ)	LT4: 5.6 nM CBZ, 6.7 nM	LT4: 10 nM–100 µM CBZ, 10 nM–500 µM	∼6 min detection; 2 h prep	Simulated blood serum	Dual redox peaks (LT4 at ∼0.3 V; CBZ at ∼0.5 V); highly porous hydrogel improved ion transport; excellent reproducibility (RSD < 2.81%), high stability (3.13% current drop after 1000 cycles), accurate concurrent detection, applicable for wearable/flexible sensing platforms	[334]
Ti_3_C_2_T_*x*_ MXene + Titania + Nafion on GC	LS‐AdSV, CV, EIS	Capsaicin	2.5 × 10^−8 ^M	5.0 × 10^−8^–2.5 × 10^−5 ^M	∼8 min incubation	BR buffer (pH 1.0); pepper extract	Good selectivity and stability. Enhanced signal via electrostatic adsorption and high conductivity of Ti_3_C_2_T_*x*_ MXene. Comparable or better performance than graphene/CNT‐based sensors. Suitable for food and pharmaceutical matrices. Capable of detecting dihydrocapsaicin and nordihydrocapsaicin	[335]
Ti_3_C_2_ MXene + SnOx + PANI on GCE	CV, DPV, EIS	Luteolin	0.12 μM	0.2–6.5 μM	40 s (enrichment)	PBS (pH 5.5); Perilla, Chrysanthemum extracts	Synergistic effect of MXene, SnOx, and PANI improved conductivity, surface area, and redox performance. Excellent selectivity and reproducibility. Real sample recovery rates were 97%–103%. Sensor demonstrates good stability over 17 days	[336]
Co@VOGO/dsDNA/Mo_2_V_2_C_3_T_*x*_ MXene@rGO on PGE	SWV, EIS	Levetiracetam	3.64 × 10^−8 ^M	5 × 10^−7^–6 × 10^−5 ^M	90s accumulation	B‐R buffer (pH 3.0), Pharmaceutical tablet, Spiked wastewater	High sensitivity due to multi‐layer synergy (MXene, rGO, DNA, Co@VOGO). Stable, reproducible. Good recovery (98%–100%) in wastewater and tablets. Excellent anti‐interference against drugs and biological species. SWV peak shifted to lower potential indicating better electrocatalysis	[337]
CoNi ZIF‐ Ti_3_C_2_T_*x*_ MXene@MWCNT on carbon cloth (CC)	DPV, CV, EIS	Salidroside	0.0958 µg/mL	0.5–500 µg/mL	∼15 min sample prep + 6 min detection	Rhodiola extract (methanol), PBS	Bacillus‐shaped composite enhanced electrocatalysis, high reproducibility and long‐term stability (3.57% signal drop in 30 days), excellent interference resistance. Detected SAL content in Rhodiola crenulata vs. Rhodiola rosea for quality comparison. Results comparable to HPLC	[338]
Ti_3_C_2_ MXene‐AuNPs + TGA + Aptamer on SPCE	DPV, CV, EIS	Digoxin	0.03 pM	0.1 pM–10 µM	30 min (incubation)	Human blood sample (spiked)	Low‐cost fabrication (<$100/sample), high selectivity (vs. codeine, caffeine, etc.), long‐term stability (>30 days), no signal loss after 3 days of use. Good electrochemical amplification from MXene and AuNPs. Better sensitivity and range than HPLC and ELISA‐based alternatives	[339]
FeCu‐LDH@ Ti_3_C_2_ MXene on GCE	DPV, CV, CC, CA, EIS	Clonazepam	90 nM	0.66–418 μM	Rapid	Human plasma, pharmaceutical tablet	Excellent sensitivity and selectivity; recovery in plasma and tablets: 98.3%–101%. Stable (94% signal retained after 30 days), highly reproducible (RSD < 2.6%). MXene and FeCu‐LDH synergy enhances conductivity and active surface area. Strong anti‐interference with common ions and molecules	[340]
MXene (Ti_3_C_2_) + MOF (UiO‐66) on carbon cloth	DPV, CV, EIS	Tanshinol	0.034 µM	0.08–8 µM	—	Salvia miltiorrhiza extract	Self‐assembled MXene/MOF composite annealed at 300°C increased surface area and electron transfer. Exhibited high selectivity and reproducibility (RSD ∼3.6%), recovery ∼99%–101%, and agreement with HPLC. Interference‐resistant, stable (95.3% after 25 days)	[341]
Ti_3_C_2_T_*x*_ MXene‐NH_2_/PPC nanofibers on Au SPCE	DPV, CV, EIS	JWH‐018 (synthetic cannabinoid)	0.68 ng/mL	0.6–2000 ng/mL	—	Saliva, PBS	Electrospun MXene‐PPC nanofibers improve surface area and sensitivity. Immunosensor showed excellent selectivity even in complex matrices. Optimized for 60 s electrospinning and 100 µg/mL antibody. Stable under vacuum for extended storage	[342]
PEI@Ti‐MOF@Ti_3_C_2_T_*x*_ MXene/AuE	Electrochemical	Zearalenone (ZEN)	1.64 fg/mL	10^−11^ to 10^−5^ ng/mL	—	Cornmeal, Beer	MXene and Ti‐MOF synergize for enhanced conductivity and aptamer loading. High recovery in real samples (87.7%–105.2%)	[343]
Thionine‐functionalized Ti_3_C_2_T_*x*_ MXene/PtAuNPs on GCE with Fc‐labeled aptamer and hDNA	ACV (ratiometric), EIS	Aflatoxin B1 (AFB1)	6.2 pg/mL (EC), 0.8 pg/mL (EIS)	0.01–30 ng/mL (ACV), 0.003–30 ng/mL (EIS)	40 min (incubation)	Corn extract	Dual signal from I_Thi_/I_Fc_ and Ret enhances reliability; selective against common mycotoxins; RSD < 4%, stable over 7 days; recoveries ∼95%–105% in corn	[344]
Ti_3_C_2_T_*x*_ MXene + aptamers for AFB1, OTA, and ZEN	CV	Aflatoxin B1 (AFB1) Ochratoxin A (OTA) Zearalenone (ZEN)	AFB1: 41.2 pg/mL OTA, 27.6 pg/mL ZEN, 33.0 pg/mL	0.1–10.0 ng/mL (for all)	—	Corn samples (spiked)	Developed a portable multichannel electrochemical device (PESD) paired with a MXene‐based electrode array (MBEAA) for simultaneous detection. Demonstrated excellent selectivity, stability, and recoveries (98.6%–105.3%) comparable to HPLC. Low‐cost (~10 CNY), highly repeatable and reproducible system	[345]
N‐doped Ti_3_C_2_T_*x*_ MXene + Mannose (NMXene‐Man)	EIS, CV	*Escherichia coli*	∼10 CFU/mL	10^1^–10^8^ CFU/mL	∼45 min	PBS, tap water	Multivalent mannose interaction with FimH lectin on *E. coli* enabled high selectivity and dual sensing and filtering; N‐doping improved surface interaction and electrochemical signal	[346]

An important trend across both neurotransmitter and pharmaceutical sensing is the increasing use of surface functionalization strategies to enhance selectivity. Among these, MIPs have been widely integrated with MXenes to create highly specific recognition sites for target analytes, enabling selective detection even in the presence of structurally similar interferents. For example, MXene‐supported MIP systems have been developed for hydrocortisone detection, achieving high selectivity and low detection limits by combining precise molecular recognition with efficient signal transduction [333]. Similarly, a ratiometric electrochemical sensor for ofloxacin was constructed using a Fe‐MOF‐NH_2_/CNTs‐NH_2_/MXene composite as the conductive matrix, coupled with a MIP layer for selective binding [330]. In this system, MXene sheets—spaced by Fe‐MOF and carbon nanotubes to prevent restacking—provide high conductivity and large accessible surface area, facilitating rapid electron transfer during ofloxacin oxidation. The incorporation of dopamine as an internal reference enables a signal‐on/off ratiometric mechanism, resulting in a wide linear range (0.1–100 μM) and a low detection limit of 13.2 nM. The sensor further demonstrates strong selectivity, reproducibility, and applicability in complex matrices such as milk samples [330].

From a mechanistic perspective, the performance of MXene‐based sensors in this domain is governed by a combination of adsorption affinity, electron transfer kinetics, and interfacial charge distribution. Negatively charged surface terminations on MXenes play a critical role in modulating analyte adsorption through electrostatic interactions, thereby influencing selectivity. At the same time, the layered architecture of MXenes supports rapid charge transport, enabling clear resolution of closely spaced redox peaks. Hybridization with complementary materials further enhances performance by introducing catalytic activity, structural stability, or selective binding functionalities. Despite these advances, several challenges remain. The simultaneous detection of multiple analytes with overlapping redox potentials still requires careful tuning of material composition and surface chemistry. Electrode fouling—particularly in biological environments—can progressively degrade sensor response. In addition, the long‐term stability of MXenes under physiological conditions remains a concern, as oxidation and structural degradation may compromise performance over time. Addressing these limitations will require the development of robust surface modification strategies, along with systematic investigations into MXene interactions with complex biological matrices. Overall, MXene‐based sensors for neurotransmitters and pharmaceutical compounds exhibit strong potential for high‐sensitivity and selective detection, particularly when engineered as hybrid systems. Future progress will depend on improving analyte discrimination, enhancing material stability, and ensuring reliable operation in real‐world applications.

### 5.5 Emerging and Advanced Platforms

Beyond conventional electrochemical configurations, MXene‐based sensing has increasingly evolved toward integrated platforms that combine material functionality with device‐level innovation. Rather than relying solely on electrode modification, these systems exploit the unique physicochemical properties of MXenes to enable new sensing modalities, enhanced portability, and real‐time analysis. This shift reflects a broader trend in electrochemical sensing, where performance is defined not only by sensitivity and detection limits, but also by system integration, operational stability, and applicability in complex or resource‐limited environments. One prominent direction is the development of wearable and flexible sensors. The intrinsic mechanical flexibility, high conductivity, and solution processability of MXenes make them well‐suited for incorporation into substrates such as textiles, polymer films, and paper. MXene‐based flexible electrodes have been applied to the detection of biomolecules and small analytes in sweat and other biofluids, maintaining reliable performance under mechanical deformation [218, 296]. Their layered structure enables conformal coating onto flexible substrates while preserving efficient electron transport pathways, a critical requirement for maintaining signal stability under bending and stretching conditions.

Another important advancement is the development of paper‐based and microfluidic platforms represent, particularly for point‐of‐care and low‐cost diagnostics. MXene inks can be readily deposited onto paper substrates to create disposable sensing devices with minimal fabrication complexity. For example, a transient paper‐based electrochemical biosensor integrating Ti_3_C_2_ MXene with Cu‐TCPP(Fe) nanosheets demonstrated a superadditive enhancement in electrocatalytic activity through strong interfacial interactions [185]. This hybrid system enabled simultaneous, non‐invasive detection of glucose and uric acid via enzyme‐mediated oxidation, with the resulting H_2_O_2_ efficiently decomposed at the Cu/MXene interface to amplify current signals. The device achieved ultralow detection limits (1.88 aM for glucose and 5.80 pM for uric acid), wide linear ranges (1.0 pM–5.0 mM and 25 pM–5.0 mM), and strong long‐term stability [185]. The hydrophilic nature of MXenes facilitates uniform coating and effective analyte interaction, while compatibility with portable electronics supports on‐site analysis. Integration with microfluidic systems further improves sample handling, reproducibility, and reagent efficiency [1, 2, 233].

Electrochemical transistor‐based sensors have also emerged as a powerful platform for MXene integration. In these devices, MXenes act as the active channel material, where interfacial reactions modulate channel conductivity. For instance, an ultrathin Ti_3_C_2_T_*x*_‐based electrochemical transistor for nitrite detection achieved nanomolar detection limits and high sensitivity through efficient coupling between the sensing interface and the transistor channel [199]. The high carrier mobility and large surface area of MXenes enable rapid transduction of interfacial charge variations, supporting real‐time monitoring without the need for conventional redox probes. Another emerging direction involves dual‐mode sensing systems that integrate electrochemical and optical readouts. By combining techniques such as colorimetry or fluorescence with electrochemical detection, these platforms enhance reliability through cross‐validation while improving usability in portable formats. MXene‐based composites incorporating plasmonic or fluorescent components have enabled simultaneous electrochemical and optical sensing, offering particular promise for point‐of‐care diagnostics [216, 233].

Across these platforms, a unifying theme is the transition from material‐centric optimization to system‐level design. MXenes serve as a versatile foundation adaptable to diverse device architectures, from flexible and wearable formats to transistor‐based and hybrid sensing systems. Their combination of conductivity, surface functionality, and processability supports the development of sensors that are not only highly sensitive but also practical and user‐oriented. However, this transition introduces new challenges. Device integration adds complexity through factors such as substrate compatibility, mechanical durability, and interface engineering. Scalable fabrication—especially for flexible and microfluidic devices—remains a significant barrier to commercialization. In addition, long‐term stability and reproducibility under real‐world conditions are critical concerns, particularly given the susceptibility of MXenes to oxidation and structural degradation. Addressing these issues will require coordinated advances in materials design, device engineering, and system‐level optimization. Overall, emerging MXene‐based sensing platforms demonstrate a clear trajectory toward integrated, versatile, and application‐driven technologies. By leveraging MXenes within advanced device architectures, these systems are well positioned to bridge the gap between laboratory‐scale demonstrations and practical sensing solutions across biomedical, environmental, and industrial domains.

## 6 Challenges in MXene‐Based Electrochemical Sensing

Despite significant progress in MXene‐based electrochemical sensors, several critical challenges continue to limit their practical implementation and large‐scale adoption. These limitations arise from both the intrinsic properties of MXenes and the variability introduced during synthesis, functionalization, and device fabrication. Addressing these issues is essential for translating MXene‐based systems from laboratory demonstrations to reliable, scalable sensing technologies [87, 347]. A primary limitation is the susceptibility of MXenes to oxidation and surface‐state evolution, particularly in aqueous and ambient environments. For Ti_3_C_2_T_*x*_ and related systems, such degradation can alter electrical conductivity and surface chemistry, directly impacting sensing performance and long‐term reliability [348, 349]. The extent of degradation is highly sensitive to environmental and processing conditions, complicating efforts to achieve consistent behavior across studies. While surface engineering strategies can improve stability, uncontrolled oxidation remains a major barrier to durable sensor operation [349].

Another key issue is the tendency of MXene nanosheets to restack due to interlayer interactions. As discussed in Section 2.3, restacking reduces accessible surface area and limits the exposure of active sites [73, 74], thereby diminishing sensitivity and hindering mass transport. This effect is particularly pronounced in film‐based devices, where aggregation during fabrication can compromise performance [347, 348]. Although intercalation, hybridization, and composite design can mitigate restacking, maintaining a stable and reproducible architecture over time remains challenging [348]. Reproducibility is further complicated by variability in MXene synthesis and post‐processing. Differences in etching chemistry, precursor composition, and delamination protocols can lead to significant variations in flake size, defect density, oxidation state, and surface terminations—all of which directly influence electrochemical behavior [349, 350]. Because these parameters are not consistently characterized or reported, sensors based on nominally similar MXenes often exhibit markedly different performance, obscuring structure–property–performance relationships [350]. Surface chemistry adds another layer of complexity. MXenes typically possess heterogeneous termination groups whose composition depends strongly on synthesis and processing history. These terminations govern adsorption behavior, interfacial charge transfer, catalytic activity, and interactions with recognition elements, often leading to variability in sensor response. Achieving precise and reproducible control over surface terminations therefore remains a key objective for application‐specific sensor design [349, 350].

In biosensing applications, additional constraints arise from interactions with complex biological environments. Surface fouling and nonspecific adsorption can degrade signal quality, while maintaining the activity and stability of immobilized biomolecular recognition elements over extended operation remains difficult. Furthermore, issues related to biocompatibility, nano–bio interfacial interactions, and long‐term functional stability require careful evaluation before practical deployment in wearable or implantable systems [87, 351]. Selectivity also remains a limiting factor, particularly in complex sample matrices containing multiple interfering species. While MXenes provide excellent conductivity and high surface area for signal transduction, selective recognition typically relies on external functionalization strategies, such as MIPs, antibodies, or aptamers. As a result, selectivity is not intrinsic to MXenes and depends strongly on interface engineering and the robustness of the recognition layer [347].

From a practical standpoint, scalability and device integration pose additional challenges. Although MXenes are solution‐processable and compatible with flexible substrates, achieving uniform, large‐scale production with consistent quality is still difficult. Variations in dispersion stability, flake dimensions, and processing conditions can affect electrode fabrication and device performance. Moreover, wearable and portable systems require further optimization of mechanical robustness, interfacial adhesion, and operational durability under real‐world conditions [87, 348]. Finally, the lack of standardized testing and reporting protocols continues to hinder meaningful comparison across studies. Variations in sample matrices, electrode preparation methods, measurement configurations, and reporting of analytical metrics complicate benchmarking, both within MXene research and across the broader electrochemical sensing field. This lack of harmonization slows the development of reliable design principles for practical applications [347, 352]. Collectively, these challenges underscore the need for a more systematic and integrated approach to MXene‐based sensor development. Progress will depend on improving material stability, ensuring reproducibility, achieving precise surface control, understanding biointerfaces, and establishing standardized evaluation protocols. Addressing these interconnected issues is essential for unlocking the full potential of MXenes in real‐world sensing applications [349, 350].

## 7 Opportunities and Future Directions

While MXene‐based electrochemical sensors have demonstrated remarkable performance across a wide range of applications, the field remains in a phase of rapid evolution, with substantial opportunities for further advancement. Building on the challenges outlined in the previous section, future research should prioritize the development of more controlled, robust, and application‐oriented MXene systems capable of bridging the gap between laboratory studies and practical deployment. Achieving this goal will require coordinated progress in material synthesis, interfacial engineering, device integration, and data‐driven design [87, 347].

A particularly promising direction is the exploration of advanced MXene chemistries, including multi‐metal and compositionally complex systems. These materials offer expanded tunability of electronic structure, catalytic activity, and surface interactions. Incorporating multiple transition metals can introduce synergistic effects that enhance electrochemical performance and extend applicability beyond conventional Ti_3_C_2_T_*x*_‐based platforms [349, 350]. Equally important is achieving precise control over surface terminations and interfacial chemistry. Surface functional groups play a central role in governing adsorption, catalytic behavior, and biomolecular interactions. The development of synthesis and post‐treatment strategies that enable controlled and reproducible surface termination profiles could significantly improve both performance and consistency. In this context, alternative synthesis routes and targeted surface modification approaches are increasingly being explored to enhance stability and biocompatibility [349, 351].

The rational integration of MXenes with complementary functional materials represents another major opportunity. Hybrid systems incorporating NPs, polymers, or biomolecular components have already demonstrated enhanced sensing performance through synergistic interactions. Moving forward, the design of such composites should be guided by a deeper mechanistic understanding of interfacial processes. In particular, MIPs and related recognition elements offer effective strategies for improving selectivity in complex sample matrices [347]. Advances in device architecture will also play a crucial role. The incorporation of MXenes into flexible, wearable, and microfluidic platforms enables portable and real‐time sensing capabilities. However, further progress will depend on improving mechanical durability, long‐term operational stability, and scalable fabrication strategies. Integration with wireless communication systems and miniaturized electronics is expected to be a key step toward practical deployment [87].

From a methodological perspective, data‐driven and computational approaches are emerging as powerful tools for accelerating MXene research. Theoretical modeling and machine learning can facilitate the prediction of structure–property relationships, guide material selection, and optimize device design. These approaches offer a pathway to reduce experimental trial‐and‐error and enable more efficient development of high‐performance sensing systems [350]. Standardization of experimental protocols and reporting practices is another critical requirement. Establishing consistent methodologies for MXene characterization and sensor evaluation will enable more reliable comparison across studies and support the development of generalizable design principles. This need for reproducibility and benchmarking is increasingly emphasized across recent MXene and electrochemical sensing literature [347, 352].

Sustainability considerations are also expected to gain importance as the field progresses. The development of safer synthesis routes, reduction of hazardous reagents, and evaluation of lifecycle impacts will be essential for large‐scale production and practical deployment, particularly in environmental and biomedical applications [348, 351]. Ultimately, translating MXene‐based electrochemical sensors into real‐world applications will require a shift from proof‐of‐concept demonstrations to system‐level validation. This includes rigorous testing in real sample matrices, assessment of long‐term stability, and integration into user‐friendly device platforms. Achieving these goals will depend on interdisciplinary collaboration spanning materials science, engineering, and application domains [87]. Overall, the future of MXene‐based electrochemical sensing lies in the convergence of advanced materials design, mechanistic insight, and practical engineering. By addressing current limitations while leveraging emerging opportunities, MXenes are well positioned to play a central role in the development of next‐generation sensing technologies [349, 350].

## 8 Conclusion

MXenes have emerged as a highly versatile class of 2D materials for electrochemical sensing, distinguished by their combination of metallic conductivity, tunable surface chemistry, and layered architecture. These properties enable MXenes to function as multifunctional platforms that facilitate rapid electron transfer, promote analyte adsorption, and support the immobilization of recognition elements. As demonstrated across diverse applications—including small molecule detection, environmental monitoring, biomolecular sensing, and pharmaceutical analysis—MXene‐based systems consistently deliver enhanced sensitivity and improved analytical performance.

A central theme of this review is the critical role of structure–property relationships in governing sensing behavior. The performance of MXene‐based sensors arises from the interplay of composition, surface terminations, interlayer structure, and functionalization strategies, rather than any single parameter. Establishing a mechanistic framework that links these features to electron transfer kinetics, catalytic activity, and mass transport provides a basis for rationalizing performance variations reported in the literature and identifying key design principles for future development. Despite these advances, several challenges continue to hinder practical implementation. Oxidation‐induced degradation, structural instability, variability in surface chemistry, and the lack of standardized evaluation protocols all contribute to limited reproducibility and long‐term reliability. Addressing these issues will require improved control over synthesis and surface engineering, along with the adoption of consistent characterization and benchmarking methodologies.

Looking ahead, continued progress in MXene chemistry—particularly the development of multi‐metal and compositionally complex systems—combined with advances in hybrid material design and device integration, is expected to further expand the capabilities of these materials. The incorporation of MXenes into flexible, wearable, and microfluidic platforms, alongside the integration of data‐driven approaches for materials optimization, will be pivotal in advancing the field toward real‐world deployment. Overall, MXenes represent a rapidly evolving and highly promising platform for electrochemical sensing. Realizing their full potential will depend on the convergence of precise materials control, mechanistic understanding, and scalable engineering. With these advances, MXene‐based systems are well positioned to play a significant role in next‐generation analytical technologies across environmental, biomedical, and industrial domains.

## Author Contributions

**Hichem Moulahoum:** data curation (lead); formal analysis (lead); investigation (lead); software (lead); validation (lead); writing – original draft (lead); writing review and editing (lead). **Faezeh Ghorbanizamani**: data curation (lead); formal analysis (lead); investigation (lead); methodology (lead); writing – original draft (lead); writing review and editing (lead). **Tomáš Kovářík**: funding acquisition (lead); project administration (lead); resources (lead); writing – review and editing (lead). **Kalim Deshmukh**: conceptualization (lead); visualization (lead); funding acquisition (lead); project administration (lead); resources (lead); supervision (lead); validation (lead); writing review and editing (lead).

## Funding

This work was supported by the Operational Programme Johannes Amos Comenius (Grant SENDISO ‐ CZ.02.01.01/00/22_008/0004596).

## Conflicts of Interest

The authors declare no conflicts of interest.

## Data Availability

The preprint copy of this article is available at: https://doi.org/10.5281/zenodo.18337960, and the dataset is available at: https://doi.org/10.5281/zenodo.18338584.
